# Screening of Bread Wheat Genotypes for Drought Tolerance Using Phenotypic and Proline Analyses

**DOI:** 10.3389/fpls.2016.01276

**Published:** 2016-08-25

**Authors:** Learnmore Mwadzingeni, Hussein Shimelis, Samson Tesfay, Toi J. Tsilo

**Affiliations:** ^1^School of Agricultural, Earth and Environmental Sciences, University of KwaZulu-NatalPietermaritzburg, South Africa; ^2^Agricultural Research Council-Small Grain InstituteBethlehem, South Africa; ^3^Department of Life and Consumer Sciences, University of South AfricaPretoria, South Africa

**Keywords:** agronomic traits, drought tolerance, proline accumulation, water stress, wheat

## Abstract

Drought stress is one of the leading constraints to wheat (*Triticum aestivum* L.) production globally. Breeding for drought tolerance using novel genetic resources is an important mitigation strategy. This study aimed to determine the level of drought tolerance among diverse bread wheat genotypes using agronomic traits and proline analyses and to establish correlation of proline content and agronomic traits under drought-stress conditions in order to select promising wheat lines for breeding. Ninety-six diverse genotypes including 88 lines from the International Maize and Wheat Improvement Center (CIMMYT)'s heat and drought nurseries, and eight local checks were evaluated under greenhouse and field conditions during 2014/15 and 2015/16 making four testing environments. The following phenotypic traits were collected after stress imposed during the heading to anthesis period: the number of days to heading (DTH), days to maturity (DTM), productive tiller number (TN), plant height (PH), spike length (SL), spikelet per spike (SPS), kernels per spike (KPS), thousand kernel weight (TKW) and grain yield (GY) and proline content (PC). Analysis of variance, Pearson's correlation coefficient, principal component and stress tolerance index were calculated. Genotypes with high yield performance under stressed and optimum conditions maintained high values for yield components. Proline content significantly increased under stress, but weakly correlated with agronomic traits under both optimal and water limited conditions. The positive correlation observed between grain yield and proline content under-drought stress conditions provides evidence that proline accumulation might ultimately be considered as a tool for effective selection of drought tolerant genotypes. The study selected 12 genotypes with high grain yields under drought stressed conditions and favorable adaptive traits useful for breeding.

## Introduction

Wheat (*Triticum aestivum* L.) is among the major staple crops, with about 720 million tons being produced globally. In sub-Saharan Africa (SSA), the crop is grown by millions of resource poor smallholder farmers predominantly under rain-fed conditions. Wheat consumption in SSA is increasing by approximately 650,000,000 tons per year (Mason et al., 2012). However, its production is projected to decrease across the continent due to recurring droughts that are associated with climate change (Knox et al., 2012). Thus, the wheat yields need to be increased in order to meet the food demands of growing populations (Ray et al., 2013). Therefore, breeding drought tolerant wheat genotypes with relevant agronomic and adaptive traits is key to enhance productivity and food security among wheat growing communities. Adoption of drought tolerant genotypes is one of the most sustainable ways to reduce the impacts of marginal rainfall and prolonged dry spells on wheat production and productivity. The International Maize and Wheat Improvement Center (CIMMYT), and other national and international breeding programs are developing drought tolerant and agronomically superior wheat lines for evaluation and utilization in breeding programs (Lantican et al., 2001; Manes et al., 2012).

Phenotyping remains a key criterion for screening breeding materials based on drought adaptive and constitutive morpho-physiological characteristics including yield and its components (Monneveux et al., 2012; Passioura, 2012). Selection for such traits through the conventional plant breeding technique has significantly improved wheat productivity under both optimum and marginal rainfall conditions. Among important agronomic traits, reduced plant height (PH) is strongly related to harvest index in dry land cereal crops especially in water limited environments (Blum, 2010). Yield components of wheat that are relevant for drought screening include the following: spikelet per spike (SPS), kennels per spike (KPS), productive tiller number (TN), and thousand seed weight (TSW). Reduced number of days to heading (DTH) and days to maturity (DTM) are also important when breeding for terminal drought stress tolerance since they allow for drought escape (Lopes et al., 2012). Typically, selection should target genotypes with relatively high yields under both stressed and optimum conditions for their improved adaption to changing climatic conditions, hence the need to determine stress tolerance index (STI) of test genotypes. Thus, there is a need to select genotypes with a good combination of agronomically important traits, cumulatively contributing to improved yields under target drought conditions (Tardieu, 2012). Selection using controlled water application with the aid of various drought indices offers effective yield based germplasm screening, allowing for selection of high yielding genotypes under both stressed and optimum conditions.

Biochemical analysis including mannitol, glycine betaine, trehalose and proline contents, have long been proposed to be useful as a complementary strategy for selection of drought tolerant genotypes in plant breeding (Abebe et al., 2003; Bowne et al., 2012; Mwadzingeni et al., 2016). However, this approach still requires validation for its usefulness in screening germplasm for improved yield under stressed conditions. Previous studies indicated that proline is among key biochemicals that accumulate in significant proportions in plants that are exposed to various kinds of stress, including dehydration (Hong-Boa et al., 2006; Khamssi, 2014). Proline, which is an α-amino acid, has been associated with several osmoprotection roles, including; osmotic adjustment (Marek et al., 2009; Zadehbagheri et al., 2014), membrane stabilization (Hayat et al., 2012), and gene signaling to activate anti-oxidizing enzymes that scavenge reactive oxygen species (ROS) (de Carvalho et al., 2013). Other studies have reported the regulation mechanisms of proline biosynthesis and degradation by enzymes such as Δ^1^- pyrroline-5-carboxylate synthetase (P5CS) and proline dehydrogenase (PDH) respectively (Kishor et al., 2005; Szabados and Savouré, 2010). Saeedipour (2013) reported that proline content accumlated faster and in higher proportions in drought tolerant genotypes than sensitive counterparts under drought-stress conditions suggesting its value in breeding for drought tolerance. Proline content has been reported to be controlled by genes with additive effects by Maleki et al. (2010).

Information on the correlation between proline accumulations at critical growth stages of wheat with drought stressed yield and other agronomic traits is limited. Most previous studies quantified proline at the seedling stages without considering the ultimate grain yield. Also, some of the studies used too few genotypes to make conclusions that are relevant to plant breeding. Exploration of proline content under severe stress in a pool of diverse genotypes at critical growth stages and description of its correlation with the yield and its component traits will provide useful information for rapid germplasm screening when breeding for drought tolerance. There is therefore a need to intensively screen a large pool of wheat breeding lines for drought tolerance using yield, yield related traits and proline analyses. The objectives of the study were to determine the genotypic variation for drought tolerance among diverse bread wheat genotypes based on agronomic traits and proline analysis, and to identify promising lines for breeding. The study was conducted with the hypotheses that proline content at a critical drought-stress stage tends to be highly correlated with agronomic traits, particularly with grain yield, hence it can be considered as a useful and complementary selection marker. Further, candidate CIMMYT wheat lines evaluated will have higher yield potential under drought-stressed and non-stressed conditions than the local checks for drought tolerance breeding.

## Materials and methods

### Plant materials and study site

The study evaluated 96 diverse bread wheat genotypes consisting of 88 lines from CIMMYT's heat and drought nurseries; and 8 local checks. The CIMMYT lines were selected based on their differential pedigrees. Supplementary Table S1 lists the details of the germplasm used in the study. The lines were evaluated under greenhouse and field conditions during 2014/15 and 2015/16 making four testing environments, hereafter referred to as E1 (greenhouse 2014/15), E2 (field 2014/15), E3 (greenhouse 2015/16), and E4 (field 205/16) at the University of KwaZulu-Natal (UKZN). The greenhouse's day/night temperatures were 30°C/20°C, while the humidity ranged between 45 and 55%. The field experiment was conducted using soil covered with a custom-made plastic mulch to exclude rainfall and soil water evaporation at UKZN's Ukulinga Research Farm (29° 40′ S, 30° 24′ E; 806 m above sea level) from mid-December to May during the 2014/15 and 2015/16 growing seasons. Based on annual averages of long term climatic data, Ukulinga has a mean annual temperature and rainfall of 18°C and 738 mm, respectively. Weather data for the periods of the field trials that is presented in Table 1.

**Table 1:** **Monthly weather data during the field trial at Ukulinga, Pietermaritzburg (2014 /15 and 2015/2016)**.

**Year**	**Month**	**Tmax (°C)**	**Tmin (°C)**	**RHmax (%)**	**RHmin (%)**	**Rs (MJ/m^2^)**	**ET0 (mm)**
2014/15	December	26.04	15.96	99.63	53.74	17.63	109.81
	January	27.76	17.1	98.3	52.28	19.69	123.21
	February	26.22	16.55	99.87	55.42	19.44	105.66
	March	27.08	16.76	96.18	48.65	17.83	108.9
	April	23.86	13.51	97.21	46.88	14.58	81.15
2015/16	December	29.29	17.42	60.36	41.76	19.57	140.68
	January	28.38	17.41	99.85	63.85	17.47	109.47
	February	29.40	17.16	99.33	62.68	18.84	108.76
	March	28.95	17.00	98.92	61.17	16.29	102.44
	April	27.47	14.72	95.96	54.08	13.22	80.70

Tmax, average maximum temperature; Tmin, average minimum temperature; RHmax, average maximum relative humidity; RHmin, average minimum relative humidity; Rs, average total radiation; ET0, average total relative evapo-transpiration.

### Experimental design and crop establishment

The 96 genotypes were evaluated using a lattice design with two replications containing six incomplete blocks with 16 genotypes each and two water regimes (under stressed and non-stressed conditions). The stressed treatment involved withholding irrigation to 35% field capacity (FC) before re-watering. Stressed treatment was induced from 50% heading to physiological maturity in order to simulate terminal drought stress. The field plots were 1.5 m long rows with inter- and in-row spacing of 45 and 15 cm respectively. Concurrent drought tolerance studies were conducted in an environmentally controlled greenhouse using pots as experimental units. Plastic pots of 5L capacity filled with composted pine bark growing media were used, with seven plants of one genotype established in each pot. Other agronomic practices were carried out following standard guidelines for wheat production in South Africa (DAFF, 2010).

### Data collection

Data on the following phenotypic traits was collected. Days to heading (DTH) were calculated as the number of days between the sowing date and the date when 50% of all the shoots in a plot had fully emerged spikes. The number of productive tillers (TN) was recorded at physiological maturity and plant height (PH) was measured in centimeters (cm) from the ground to the tip of the spike from five randomly sampled and tagged plants in each plot before harvesting. Days to maturity (DTM) were calculated from sowing date to 50% senescence of the spikes. The spike length (SL) [measured in cm], the number of spikelets per spike (SPS) and the numbers of kennels per spike (KPS) were recorded after harvesting from the main tillers of five randomly selected plants. Thousand seed weight (TSW) was determined using a sensitive balance measured from randomly sampled 1000 seeds after harvest and expressed in g/1000 seed. Finally grain yield per plot (GY) was determined as the weight (grams) of the grain from a plot; where the plot sizes were 1.5 m rows with 30 plants, and seven plants per pot for the field and the greenhouse experiments respectively. From the pot experiment grain yield was extrapolated based on 30 plants to agree with field data.

### Determination of proline content

Proline analysis was carried out at the University of KwaZulu Natal's Crop Science laboratories. Samples of the second top leaves from the flag leaf were harvested from the stressed and none stressed plots of the two greenhouse experiments. The leaf samples were temporarily stored at ultra-low temperature (−74°C) then freeze dried. The dry leaf tissue was ground and 0.1 g samples were homogenized in 10 mls of 3% aqueous sulfosalicylic acid. Proline extraction was done following the acid-ninhydrin method according to Bates et al. (1973). This was followed by UV-visible spectrophotometer analysis of the absorbance of the proline extract in toluene at a wavelength of 520 nm, using a model UV-1800 spectrophotometer, Shimadzu Corporation, Kyoto, Japan. The proline concentration was calculated using the following formula:

$$\begin{array}{l} \text{Proline content}(\mu \text{g per gram of dry leaf tissue})\\ =[(\mu gproline/ml)\times mltoluene)/115.5\mu \text{g}/\mu mole]/\\ {}[(gsample)/5]. \end{array}$$

Where, 115.5 is the molecular weight of proline (Bates et al., 1973).

### Data analysis

Phenotypic and proline data were analyzed separately following the lattice procedure of SAS 9.3 (SAS, 2011) and GenStat® version 17, VSN, International (Payne, 2014). Combined analysis of variance was performed following a test of homogeneity of variances. To describe the magnitude of the relationships among agronomic traits and proline content, Pearson's correlation coefficients (*r*) were calculated separately for the stress and control treatments using the SPSS version 23 (Spss, 2012). Principle component analysis (PCA) based on the correlation matrix was performed using SPSS to identify influential traits for selection. PCA biplots were plotted separately for the stressed and optimum conditions using GenStat to show the relationships among studied genotypes based on recorded traits. To select for high yielding genotypes under stressed and non-stressed conditions stress tolerance index (STI) was calculated using the following formula according to (Fernandez, 1992):

$$\begin{array}{l} \text{STI}=\left(\text{Yp}*\text{Ys}\right)/{\left(\text{Xp}\right)}^{2}; \end{array}$$

where Ys = grain yield of a test genotype under drought-stressed condition; Yp = grain yield of a test genotype under non-stressed condition, and Xp = mean yield of test genotypes under non-stressed condition.

## Results

### Effect of genotypes, water regimes, and testing environments on agronomic performance and proline content

Separate analysis of variance showed significant (*P* < 0.05) effects of the genotype, water regime, environments and their interactions for the studied traits, hence, combined analysis of variance was carried out. Table 2 summarizes the results from the combined analysis of variance for agronomic traits and proline content. Highly significant differences were observed among the main effects of genotypes, water regimes, environments, and their interactions for most traits. DTH, DTM, SL, and SPS were non-significantly affected by the interaction of the genotype by water regime and environment by genotype by water regime, while TN showed non-significant effects of the genotype by water regime by environment interaction only.

**Table 2:** **Mean squares and significant tests after combined analysis of variance for nine phenotypic traits and proline content of 96 wheat genotypes evaluated across the four test environments and two water regimes**.

**Agronomic traits**											**Proline content**	
**Sources of variation**	**DF**	**DTH**	**DTM**	**TN**	**PH**	**SL**	**SPS**	**KPS**	**TSW**	**GY**	**DF**	**PC**
Gen	95	346.83^**^	199.35^**^	3.15^**^	729.28^**^	18.76^**^	31.33^**^	340.26^**^	193.22^**^	9229.26^**^	95	10392.18^**^
WR	1	47.83^*^	6651.85^**^	455.43^**^	7791.86^**^	7.01^**^	83.45^**^	5128.34^**^	6804.68^**^	1978219^**^	1	3330364^**^
Env	3	6324.15^**^	44781.15^**^	118.90^**^	7888.87^**^	289.99^**^	2215.79^**^	30244.35^**^	2664.44^*^	1252898^**^	1	985417.73^**^
Gen.WR	95	9.201 ns	18.27 ns	1.64^**^	40.47^**^	0.25 ns	1.64 ns	43.79^**^	27.91^*^	4287.05^**^	95	10395.28^**^
Gen.Env	285	43.72^**^	53.12^**^	0.86^**^	54.85^**^	0.58^**^	3.06^**^	34.09^*^	44.19^**^	2666.05^**^	95	8014.72^**^
Env.WR	3	45.07^*^	450.59^**^	2.19^*^	1064.72^**^	4.28^**^	44.10^**^	387.08^**^	264.56^**^	22525.67^**^	1	1730641.89^**^
Env.Gen.WR	285	9.47 ns	18.77 ns	0.65 ns	29.14^*^	0.29 ns	1.74 ns	30.94^*^	23.17^*^	2171.13^*^	95	7710.46^**^
Residual	765	9.14	21.43	0.64	24.77	0.28	1.68	26.11	19.65	1736.83	382	45.67

DTH, days to 50% heading; Env, testing environment; Gen, genotype; PH, plant height; TN, number of productive tillers; DTM, days to maturity; SL, spike length; SPS, number of spikelets per spike; KPS, number of kennels per plant; TSW, thousand seed weight; GY, grain yield per plot; PC, proline content; WR, water regime;

* P < 0.05;

** P < 0.01;

ns, non-significant difference.

Table 3 summarizes the mean values; standard error of differences (SED), least significant differences (LSD) at 5% significant levels, and coefficients of variation (CVs) obtained for all traits recorded in the two water regimes. The table shows the best fifteen and bottom five genotypes in terms of grain yield under stressed conditions. Supplementary Table S2 presents the mean values obtained for all traits recorded across all testing environments and water regimes. Pooled means for all the studied traits on all genotypes under the two contrasting water regimes are presented in Supplementary Table S3. The table highlights in bold the best 20 genotypes in terms of grain yield under stressed conditions. Significant differences were noted in the overall means of the different variables recorded. Significant differences were noted in the overall means of the different variables recorded. The mean DTH was 53.62 with the earliest genotypes being the local checks LM66 and LM67 which took 43 and 43.63 days to heading respectively, and the latest genotype was LM100 from the heat nursery which took 61.88 days. The mean plant heights under stressed and optimum conditions were 73.52 and 78.03 cm, respectively. Under stressed conditions, the shortest genotype was the local cultivar LM67 (58.51 cm), while the tallest was LM77 (89.88 cm) from the drought nursery. The lines LM90, LM84, and LM100 were the tallest under optimum conditions with average height of 90.68, 90.53, and 90.06 respectively, while genotype LM53 was the shortest (61.18 cm).

**Table 3:** **Means for nine agronomic traits and proline content of 96 wheat genotypes and the top 15 best and five bottom performing genotypes when evaluated under stressed and non-stressed across the test environments, ranked according to their performance under stressed conditions**.

**Entry**	**DTH**		**DTM**		**TN**		**PH**		**SL**		**SPS**		**KPS**		**TSW**		**GY1**		**PC**	
	**WR1**	**WR2**	**WR1**	**WR2**	**WR1**	**WR2**	**WR1**	**WR2**	**WR1**	**WR2**	**WR1**	**WR2**	**WR1**	**WR2**	**WR1**	**WR2**	**WR1**	**WR2**	**WR1**	**WR2**
**TOP FIFTEEN GENOTYPES**																				
LM29	55.63	58.13	103.12	107.25	3.80	4.66	76.28	79.20	8.39	8.77	15.03	15.53	37.37	38.85	34.70	37.20	149.70	205.00	67.89	46.72
LM22	56.24	57.50	101.12	104.12	4.09	4.73	74.61	79.06	8.59	8.72	14.68	15.35	32.20	38.72	32.49	36.37	143.30	201.10	214.89	26.00
LM04	56.25	56.88	101.87	105.00	3.48	4.19	76.93	77.68	10.41	10.04	16.85	16.33	39.40	41.05	33.43	36.51	138.90	192.20	93.77	13.08
LM77	55.00	55.00	99.75	103.00	3.70	4.14	87.90	89.71	9.10	9.10	14.45	14.43	31.72	33.50	36.79	45.43	136.20	194.00	86.52	24.12
LM15	57.50	56.88	103.12	107.75	3.59	4.61	75.95	85.81	9.61	9.92	15.58	16.98	37.62	45.90	30.33	36.47	135.00	231.70	307.70	24.92
LM71	53.88	53.88	94.87	101.87	4.05	4.86	75.31	80.01	9.29	9.46	14.43	15.05	34.10	36.87	29.44	32.86	131.50	181.90	97.37	42.91
LM23	56.25	57.50	98.87	103.50	3.95	6.08	80.68	88.64	9.54	10.15	14.78	16.23	34.52	36.12	32.16	38.50	128.80	258.40	217.09	19.58
LM100	61.88	61.88	106.00	108.00	3.48	4.03	79.49	90.06	9.77	10.04	14.73	15.80	33.57	41.07	36.50	38.77	127.20	198.10	204.71	23.08
LM27	54.38	56.25	95.50	98.25	3.41	3.97	76.60	75.03	8.37	8.20	15.78	15.60	37.75	39.27	31.96	33.56	126.90	162.30	82.92	25.84
LM85	56.88	56.25	100.25	104.00	3.74	4.86	72.83	79.82	8.60	8.92	15.03	15.67	35.47	35.59	30.75	35.09	126.50	225.50	78.56	28.96
LM96	55.63	55.63	102.75	106.25	4.08	4.65	83.28	88.27	7.89	7.99	15.23	15.13	35.47	36.05	27.99	35.69	126.30	179.20	322.91	23.36
LM03	51.50	51.88	91.75	100.00	3.79	4.53	83.16	86.58	9.50	9.54	15.88	15.88	36.07	38.15	28.07	39.98	126.00	210.30	159.30	20.22
LM31	52.00	54.38	96.50	101.87	3.52	4.24	78.32	78.34	9.67	9.24	14.13	13.30	32.72	30.79	33.11	42.82	125.50	175.00	192.59	28.92
LM35	52.00	50.38	97.87	101.62	3.52	5.30	76.85	82.63	8.35	8.71	15.30	15.85	37.45	39.67	30.96	34.31	125.30	226.20	176.90	20.44
LM44	52.63	54.38	94.75	98.12	3.43	3.95	83.02	85.77	9.82	10.00	15.73	16.00	42.02	45.47	26.87	28.93	123.80	162.50	97.96	19.56
**BOTTOM FIVE GENOTYPES**																				
LM20	61.25	60.00	103.72	106.00	2.86	4.32	69.93	72.73	9.14	8.95	15.75	15.68	22.47	29.82	37.10	38.34	69.90	151.30	190.68	25.39
LM95	45.50	48.00	94.50	97.37	2.57	2.81	60.90	62.54	7.08	6.97	12.33	12.60	29.30	30.35	28.68	31.45	64.30	79.90	75.44	16.02
LM68	48.13	50.63	96.25	101.75	3.39	4.28	59.05	64.64	6.67	7.06	13.07	14.68	30.23	36.42	21.05	24.89	60.90	130.80	234.88	15.06
LM62	60.63	58.13	104.87	106.87	1.99	3.33	64.06	65.64	9.78	10.21	15.78	17.14	32.20	39.75	27.15	30.71	58.70	124.40	165.07	26.65
LM61	45.38	44.75	93.12	96.62	3.32	3.52	59.92	62.26	5.97	5.96	10.98	10.63	19.72	20.80	29.09	30.18	50.30	75.00	85.99	16.53
Mean	53.45	53.80	98.97	103.13	3.36	4.45	73.52	78.03	8.65	8.79	14.71	15.18	32.87	36.52	30.72	34.93	104.83	176.60	156.20	24.50
SED	1.07	1.07	1.64	1.64	0.28	0.28	1.76	1.76	0.19	0.19	0.94	0.94	1.81	1.81	3.20	3.20	29.79	29.79	3.38	3.38
LSD (5%)	4.41	4.41	6.77	6.77	0.55	0.55	7.08	7.08	0.74	0.74	0.90	0.90	3.55	3.55	6.28	6.28	28.93	28.93	6.64	6.64
CV (%)	5.60	5.60	4.60	4.60	20.50	20.50	6.60	6.60	6.00	6.00	8.70	8.70	14.70	14.70	13.50	13.50	29.60	29.60	7.50	7.50

DTH, days to 50% heading; CV, coefficient of variation; DTM, days to maturity; E1, test environment 1 (greenhouse 2014/15); E2, test environment 2 (field 2014/15); E3, test environment 3 (greenhouse 2015/16); E4, test environment 4 (field 2015/16); GY, grain yield per plot; LSD, list significant difference; KPS, number of kennels per plant; PH, plant height; SED; standard error of differences; SPS, number of spikelets per spike; SL, spike length; TN, number of productive tillers; TKW, thousand kernel weight; WR, water regime (water-stressed), WR2, water regime 2 (control).

A reduction in average tiller numbers was observed from 4.45 to 3.36 due to severe drought stress. Genotypes LM64 and LM84 developed the highest number of productive tillers, 4.74 and 6.33, under stressed and optimum conditions, respectively; while LM62 and LM95 had the least number of tillers, 1.99 and 2.81, under stressed and optimum conditions, respectively. A slight decrease in average spike length from 8.79 cm under optimum growing conditions to 8.65 cm under stress was observed. Average DTM were slightly lower under stress (98.97 days) than under optimum conditions (103.13 days). Lines LM89 which took 106 days to mature was the latest under stress while lines LM84 (109.62 days) and LM49 (109.87 days) were among the latest genotypes under optimum conditions. LM03 which took 91 days to maturity and LM08 which matured after 94.37 days were the earliest under stressed and optimum conditions, respectively. Means of SPS, KPS, and TSW under stress were slightly lower than the values under optimum conditions (Table 3). The average grain yield per plot was reduced by 40.64% under stress as compared to the control. The minimum and maximum stress tolerance index were 0.12 and 1.0 observed on the genotypes LM61 and LM23 respectively. Mean STI was 0.60 with 75% of the genotypes having above average STI.

Proline content varied significantly among genotypes, water regimes and the genotype by water regime interactions. Water regime accounted for much of the variation observed, explaining 54.75% of the variation in proline content. The genotype explained only 0.17% while testing environments, genotype by water regime, genotype by environment and genotype by water regime by environment interactions accounted for 16.2, 0.17, 0.13, and 0.13% respectively (Table 2). The mean PC was 24.5 μg and 156.2 μg per gram of dry leaf tissue under optimum and stressed conditions, respectively. The highest PC contents were 381.18 and 46.72 μg/gram of dry leaf sample, obtained from lines LM41 and LM29 under stressed and optimum conditions, respectively (Table 3).

### Correlations of phenotypic traits and proline content

Table 4 summarizes correlation coefficients (r) describing the degree of correlations among measured agronomic traits and proline content. The number of days to heading showed strong significant and positive correlation (*r* > 0.5, *P* < 0.05) with most of the variables recorded under well-watered conditions except for TSW and PC. Under stress, the number of days to heading highly and significantly correlated with DTM, PH, SL, and SPS, and exhibited a weak negative correlation with TN. Plant height significantly correlated with all traits except proline content under both stressed and well-watered conditions, as well as with the number of days to maturity under stressed conditions. Notably, productive tiller numbers showed strong positive correlations with GY under both stressed and optimum conditions. Days to maturity had strong positive correlations with DTH under both stressed and optimum conditions, but with weak negative and insignificant correlations with TN and PC. Further, spike length had strong positive and significant correlations with DTH, PH, SPS, and KPS under both stressed and optimum conditions, as well as with grain yield under well-watered conditions. Grain yield under stress was highly correlated with TN, with moderately high correlations with PH, KPS, and TSW under stress. On the other hand, under optimum conditions, grain yield was highly and significantly correlated with all yield components except TSW which showed moderate correlation. Proline content had weak positive and non-significant correlations (*r* < 0.3, *P* > 0.05) with all traits under both stressed and optimum conditions, except for DTM and TSW which were weak and negatively correlated with PC under stress.

**Table 4:** **Pearson's correlation coefficients (*r*) describing association of nine phenotypic traits and proline content of 96 wheat genotypes evaluated under two greenhouse and two field experiments of stressed (lower diagonal) and optimal (upper diagonal) conditions**.

**Optimum conditions**											
		**DTH**	**DTM**	**TN**	**PH**	**SL**	**SPS**	**KPS**	**TSW**	**GY**	**PC**
Stressed conditions	DTH	1	0.776^**^	0.178 ns	0.689^**^	0.648^**^	0.749^**^	0.491^**^	0.269^**^	0.498^**^	0.178 ns
	DTM	0.723^**^	1	0.092 ns	0.472^**^	0.473^**^	0.581^**^	0.365^**^	0.216^*^	0.349^**^	0.126 ns
	TN	−0.299^**^	−0.135 ns	1	0.251^*^	0.047 ns	0.059 ns	0.033 ns	0.04	0.653^**^	0.199 ns
	PH	0.557^**^	0.191 ns	−0.106	1	0.687^**^	0.611^**^	0.461^**^	0.473^**^	0.634^**^	0.161 ns
	SL	0.630^**^	0.300^**^	−0.370^**^	0.619^**^	1	0.727^**^	0.603^**^	0.292^**^	0.510^**^	0.069 ns
	SPS	0.709^**^	0.386^**^	−0.394^**^	0.618^**^	0.725^**^	1	0.773^**^	0.141 ns	0.547^**^	0.122 ns
	KPS	0.297^**^	0.034 ns	−0.238^*^	0.500^**^	0.530^**^	0.668^**^	1	−0.104	0.593^**^	0.081 ns
	TSW	0.308^**^	0.398^**^	−0.062	0.254^*^	0.215^*^	0.136 ns	−0.209^*^	1	0.414^**^	0.078 ns
	GY	0.141 ns	0.115 ns	0.543^**^	0.443^**^	0.244^*^	0.270^**^	0.466^**^	0.336^**^	1	0.197 ns
	PC	0.002 ns	−0.043 ns	0.118 ns	0.030 ns	0.170 ns	0.057 ns	0.138 ns	−0.218^*^	0.080 ns	1

DTH, days to 50% heading; DTM, days to maturity; GY, grain yield per plot; KPS, number of kennels per plant; PC, proline content; PH, plant height; TN, number of productive tillers; SL, spike length; SPS, number of spikelets per spike; TSW, thousand seed weight;

* P < 0.05 (2-tailed);

** P < 0.01 level (2-tailed);

ns, non-significant.

### Principal component analysis (PCA)

The rotated component matrix (Table 5) shows the proportion of total variance explained by different principal components and their correlations with variable traits. From the stress treatment, three principal components were important, contributing 72.44% of the total variation observed. The first two principal components were the most influential with a cumulative contribution to the total variation of 56.44%. Variables SPS, SL, KPS, PH, and DTH had high positive loading into the first principle component while DTH, TSW, and DTM had high positive loading into the second principal component. These were followed by GY and PC which had high positive loading into the third principal components respectively. Similarly, three principal components were important under optimum conditions, accounting for 73.38% of the total variation of which 61.92% was accounted for by the first two components. All traits except TN, PC, and TSW had high positive loading into the first principal component while TN had high positive loading into the second principal component.

**Table 5:** **Rotated component matrix of nine phenotypic traits and proline content of 98 wheat genotypes evaluated in four test environments under stressed and optimum conditions**.

**Stress**				**Control**			
**Trait**	**PC-1**	**PC-2**	**PC-3**	**Trait**	**PC-1**	**PC-2**	**PC-3**
SPS	0.908	0.097	−0.097	SPS	0.864	−0.315	0.145
SL	0.854	0.095	−0.071	DTH	0.862	−0.127	−0.100
KPS	0.791	−0.397	0.119	PH	0.835	0.130	−0.222
PH	0.764	0.119	0.251	SL	0.817	−0.222	−0.092
DTH	0.725	0.513	−0.13	GY	0.778	0.470	0.181
TSW	0.104	0.802	0.246	KPS	0.715	−0.337	0.455
DTM	0.361	0.712	−0.058	DTM	0.701	−0.184	−0.150
PC	0.196	−0.442	0.141	TN	0.296	0.782	0.388
GY	0.375	0.058	0.89	PC	0.224	0.383	0.188
TN	−0.372	−0.078	0.838	TSW	0.378	0.376	−0.780
Explained variance (eigenvalue)	3.934	1.71	1.6	Explained variance (eigenvalue)	4.743	1.449	1.146
Proportion of total variance (%)	39.34	17.1	16.004	Proportion of total variance (%)	47.432	14.487	11.458
Cumulative variance (%)	39.34	56.44	72.444	Cumulative variance (%)	47.432	61.919	73.377

DTH, days to 50% heading; DTM, days to maturity; GY, grain yield per plot; KPS, number of kennels per plant; PC, proline content; PC-1, principal component 1; PC-2, principal component 2; PC-3, principal component 3; PH, plant height; TN, number of productive tillers; SL, spike length; SPS, number of spikelets per spike; TSW, thousand seed weight.

### Principal component biplot analysis

**Figure 1:**
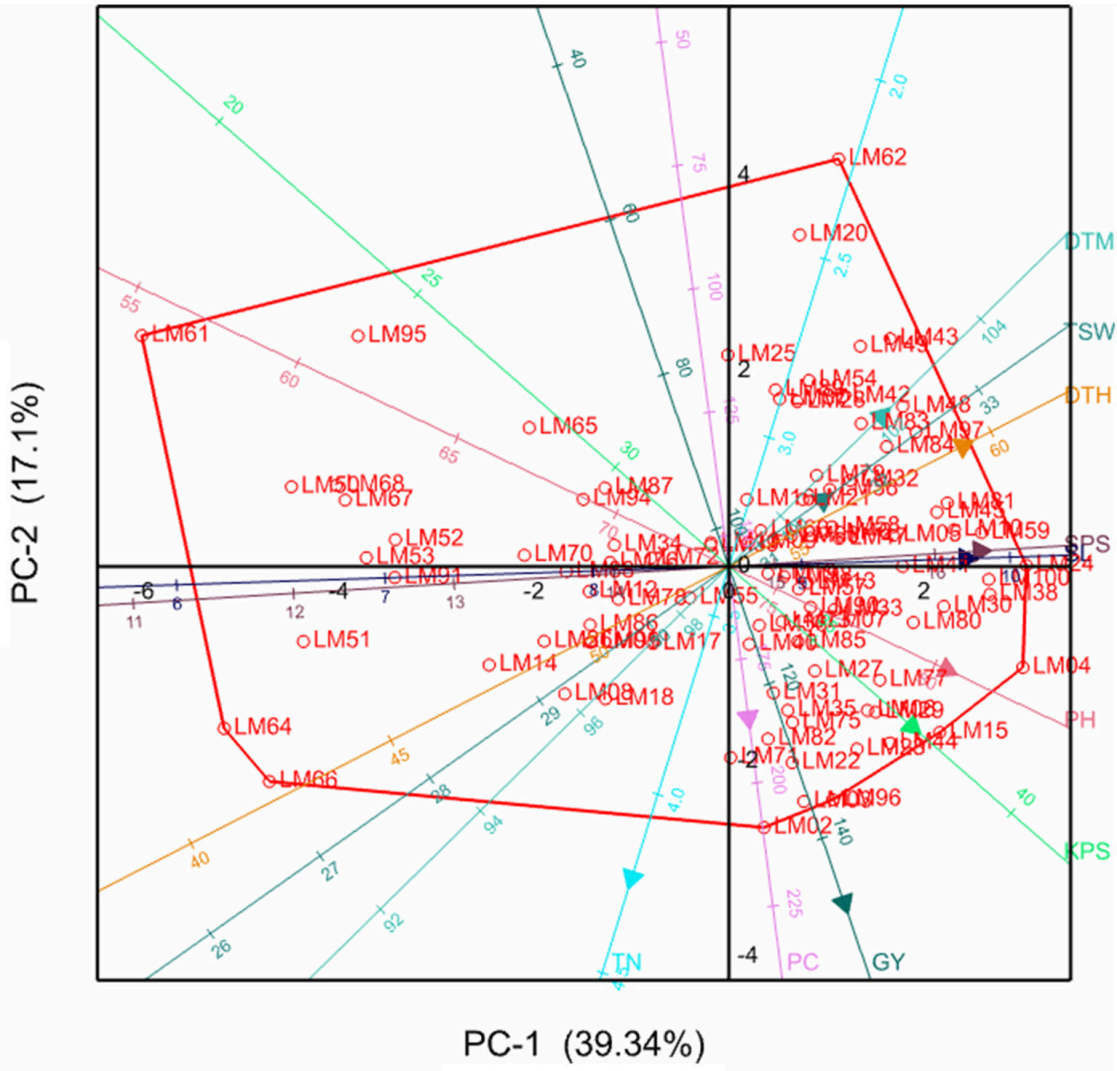
**Principal component biplot showing genotypic grouping under stress**. DTH, days to 50% heading; DTM, days to maturity; GY, grain yield per plot; SL, spike length; KPS, number of kennels per plant; PC, proline content; PH, plant height; TN, number of productive tillers; SPS, number of spikelets per spike; TSW, thousand seed weight.

**Figure 2:**
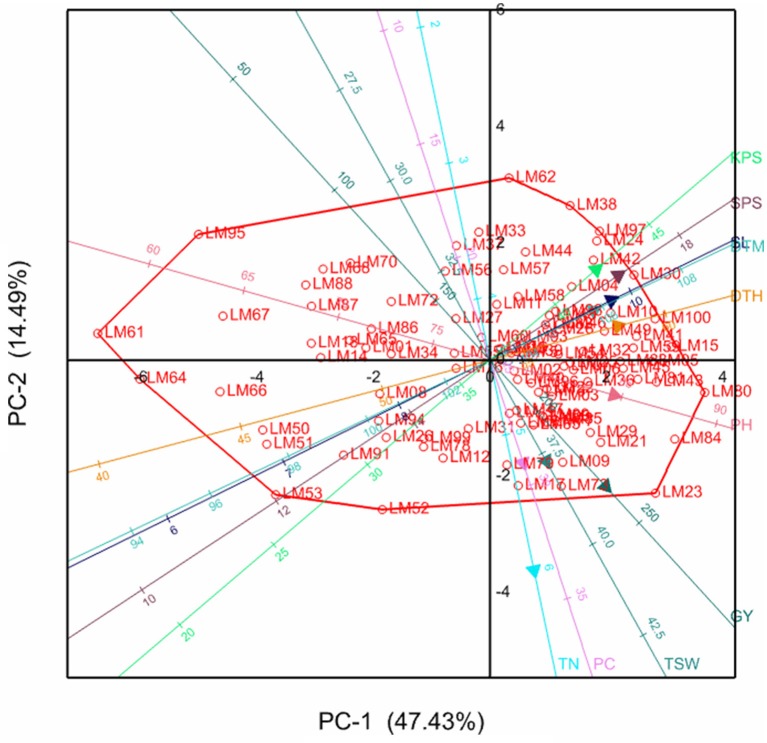
**Principal component biplot showing genotypic grouping under optimum conditions**. DTH, days to 50% heading; DTM, days to maturity; GY, grain yield per plot; SL, spike length; KPS, number of kennels per plant; PC, proline content; PH, plant height; TN, number of productive tillers; SPS, number of spikelets per spike; TSW, thousand seed weight.

The relationships between the different variables and genotypes with respective principal components are further illustrated by the principal component biplots in Figures 1, 2 for the stressed and optimum conditions respectively. Smaller angles between dimension vectors in the same direction indicated high correlation of the variable traits in terms of discriminating genotypes. Genotypes excelling in a particular trait were plotted closer to the vector line and further in the direction of that particular vector, often on the vertices of the convex hull. Under stress, most of the genotypes were scattered in the positive side of the first principal component, with genotypes such as LM22, LM96, LM02, and LM15 excelling in yield which was contributed mostly by high tiller numbers and KPS, as well as optimum values for other yield components (Figure 1). Under optimum conditions, the genotypes were also more concentrated on the positive side of the first principal component with genotype LM09, LM17, LM80, LM84, and LM23 being more inclined in the direction of GY, PC, PH, TSW, and TN (Figure 2). The local checks LM61, LM64, LM66, and LM67, and line LM95 clustered together in the direction of early heading and short stem height.

## Discussion

Development of drought tolerant wheat genotypes is the goal of wheat breeders. Effective germplasm screening for drought tolerance particularly under managed drought conditions is an effective way of selecting materials for advanced breeding programs. The highly significant genotypic differences observed among all the traits recorded indicate that the germplasm pool used in this study could be a rich source of genetic diversity for breeding purposes (Table 2). Thus, the germplasm pool can be used to identify genotypes with high levels of tolerance to water stress, as indicated by differential genotypic responses to the two water regimes. The observed significant effects of the wheat genotypes, water regimes and genotype by water regime interaction were expected since all the genotypes utilized in the study were selected from diverse pedigrees and most of the traits recorded are quantitatively inherited, hence differentially respond to the environment.

### Effect of genotypes and water regime on grain yield

Selecting for improved grain yield under both stressed and optimum conditions allow genotypes to maintain ranks for high yields since the same genotypes will be expected to perform well in either situation. The observed maintenance of high yields under stressed and optimum conditions in some genotypes; such as LM03, LM23, and LM85 supports the findings of Foulkes et al. (2007) that genotypes performing well under optimum conditions retain high yield under stress. However, the high cross-over interactions observed in this study was due to severe stress imposed on the genotypes resulting in average yield losses of about 40.64% compared to 26% observed under mild stress imposed by Foulkes et al. (2007). Interestingly, twenty-two genotypes from the heat and drought nurseries including LM15, LM22, LM29, LM27, LM77, and LM96 yielded better than all local checks under stress. Generally, most of the materials from the heat and drought nurseries were better adapted to the summer planting than the local checks, therefore, can provide useful diversity for spring cultivation.

### Association of agronomic traits under different water regimes and testing environments

Moderate to high positive and significant correlations (*r* > 0.3) of GY with TN, KPS, and TSW under both stressed and optimum conditions, shown in Table 4, imply the direct contribution of these yield components to yield and should be considered as important target traits during selection, as is supported by the findings of Dodig et al. (2012) and Sareen et al. (2014). This resulted in high stressed GY in lines such as LM22, LM29, LM77, LM15, LM24, and LM100. From Figure 1, it can be confirmed that maintenance of a high number of productive tillers and kernels per spike contributes more (acute angles) to the grain yield when compared to the other yield components under stress because the number of grains produced per plant will compensate better for the reduction in seed weight (Slafer et al., 2014). However, under optimum conditions (Figure 2), all the yield components have considerable contribution to grain yield implying that selection for any of the yield components could significantly improve the yields. Late maturing and tall genotypes have enough time and capacity to accumulate photo-assimilates resulting in higher grain yields, which explains the moderate to high correlation of DTH, DTM, PH, and SL with GY under optimum conditions. However, under stress, genotypes excelling in the former traits succumbed to drought stress due to high evapo-transpiration losses and ultimately suffered much yield loses. This resulted in the moderate to low correlations of DTH, DTM, PH, and SL with GY under water stress. This could be the reason for the fall in ranks under stress of most genotypes including LM23 and LM80 which excelled under optimum conditions. However, plant height could also be associated with dipper and extensive rooting systems since some tall genotypes such as LM23 and LM03 maintained high yield under both stressed and well-watered conditions. Genotypes with high yield under both stressed and non-stressed conditions exhibited high STI which further confirm the reliability of this index in selecting for high productivity under either condition (Fernandez, 1992).

Early heading and maturity have an advantage of allowing drought escape, enabling the genotype to efficiently utilize irrigation or rainfall during critical growth stages (Blum, 2010). However, the plant cycle should not be too short and the plant size should not be too small since such traits will compromise yields in either situation as evidenced by a yield penalty in earliest and shortest genotypes like LM70, LM95 and the local checks LM61 and 68. This is in agreement with the findings of Butler et al. (2005) where short wheat genotypes with two alleles for dwarfness; *Rht-B1b* and *Rht-D1b*, yielded lower than those with one or none of the dwarfing alleles, under both stressed and optimum conditions. The findings may be attributed to low capacity to accumulate sufficient stem reserves for subsequent partitioning to the grain (Borrell et al., 1993). The local check LM66 was among early and short genotypes and excelled in stressed yield by its ability to maintain a high number of productive tillers and a relatively high TSW. This could have resulted from a lengthy grain filling period (Dodig et al., 2012). However, the small plant stature compromises other yield components of such genotypes, thereby reducing the rank in yield potential under optimum conditions.

The principal component analysis indicated that under stress; SPS, SL, DTH, PH, and KPS have much influence during selection and can be selected together followed by TSW and DTM respectively (Table 5). This further emphasizes the importance of selecting genotypes based on yield components which could result in simultaneous selection for complementary genes adding up to yield. Putting much concentration on few major genes may result in increased survival rate at the expense of grain yield (Passioura, 2012). Under optimum conditions, high positive loading of SPS, DTH, PH, SL, GY, KPS, and DTM into the first principal component indicate that they have much influence and can be simultaneously selected for because of their direct influence on each other (Table 5). This could be explained by the fact that genotypes with longer life cycles and increased plant height have more time for photo-assimilate production and have the capacity to accumulate more biomass, hence they will have high grain yield.

### Effect of water regime on proline accumulation

The variation in proline content observed among different genotypes under both stressed and well-watered conditions and its accumulation under stress was in accordance with previous findings. Rampino et al. (2006), Vendruscolo et al. (2007), Bowne et al. (2012), and Qayyum et al. (2013) reported genotypic differences in proline concentration, and in proline accumulation in wheat genotypes exposed to water stress. Nio et al. (2011) reported of increased proline content in wheat exposed to stress, implying some levels of osmotic adjustment. Similar effects of water stress and increased PC were observed in other crops, including sugar beet (*Beta vulgaris*) (Gzik, 1996), alfalfa (*Medicago sativa*) (Irigoyen et al., 1992), and pea (*Pisum sativum*) (Sánchez et al., 1998).

The weak positive and non-significant correlation observed between the proline content and stressed yield under controlled environment could suggest that, although proline plays an important role of osmoprotection, it may not be a good reflection of stressed yield levels. These findings are supported by Tardieu (2005) who argued that genes encoding desiccation tolerance may not enhance yield under agricultural drought. The findings from this study are also reported by Marek et al. (2009) who observed low and non-significant correlations of proline content and grain yield (*r* = 0.32; *p* = 0.188) under drought stress. The observations do not support our hypothesis that proline can serve as an important biochemical marker or selection indices for indirect selection for stressed yield, which is of breeders' interests. However, the presence of positive correlation between proline content and grain yield suggests that PC remains an important trait in enhancing the capacity of genotypes to optimize grain yields under drought-stress. Despite the poor correlation of proline content with stressed yield, Figure 1 suggests that some genotypes take advantage of the capacity to accumulate more proline under stress as was noted in the drought tolerant wheat cultivar, Chinese Spring, when compared to the susceptible cultivar, SQ1 (Marcińska et al., 2013). There is therefore a need to take advantage of such genotypes. These results provide a good practical insight and add on to previous studies that used external osmotica such as polyethylene glycol (PEG), which may need to be confirmed by actual soil water deficit. Some of the studies evaluated a small number of genotypes which needed to be increased to make meaningful conclusions and recommendations for breeding. Others determined the proline accumulation at seedling stage which needed to be confirmed by genotypic responses when exposed to water stress at critical growth stages. The positive correlation of grain yield with proline content under drought stressed conditions observed in the present study supported these previous studies in that proline accumulation is a good indicator of drought tolerance in wheat which could be useful during genotype selection.

## Conclusion

Proline accumulates under stress, but proline, when measured at a single time point, may not serve as a good predictor or marker for indirect selection for drought stressed yield under agricultural conditions. However, the positive correlation of grain yield and proline content found under-drought stress conditions provides already evidence that proline accumulation might ultimately be considered as a tool for effective selection. Further studies are required to quantify proline content of diverse genotypes at different stress levels to explore the rate of proline accumulation in different genotypes during time of stress exposure and yield potential of genotypes. This could be done using a pool of well characterized drought tolerant and a contrasting set of drought susceptible genotypes. The current study also deduced that the material evaluated contain useful genetic diversity for drought tolerance. Promising lines that are highlighted bold in Supplementary Table S3 have been selected for use in breeding for drought tolerance based on their diverse and complementary agronomic traits that could further enhance drought stressed yield. The currently selected lines showed higher mean grain yields under drought-stress and higher stress tolerance indices than the local checks (LM61 to LM70) (Supplementary Table S3). The lines are part of CIMMYT nursery distributed worldwide. In South Africa, they will add to the germplasm pool identified by Dube et al. (2016).

## Author contributions

All authors listed, have made substantial, direct and intellectual contribution to the work, and approved it for publication.

### Conflict of interest statement

The authors declare that the research was conducted in the absence of any commercial or financial relationships that could be construed as a potential conflict of interest.
