# Supplementary material for: Screening of Bread Wheat Genotypes for Drought Tolerance Using Phenotypic and Proline Analyses
Source: Front Plant Sci. 2016 Aug 25;7:1276. doi: 10.3389/fpls.2016.01276 (PMC4997044; doi:10.3389/fpls.2016.01276)
Supplement: Supplementary file 1 [file Table1.DOCX]

**Electronic Supplementary Material**

**Screening of bread wheat genotypes for drought tolerance using phenotypic and proline analyses**

### Journal: Frontiers in Plant Science, Section [Crop Science and Horticulture](https://www.google.co.za/url?sa=t&rct=j&q=&esrc=s&source=web&cd=3&ved=0ahUKEwiM1a6b3InNAhWqLMAKHVkSBvcQjBAIJTAC&url=http%3A%2F%2Fjournal.frontiersin.org%2Fjournal%2Fplant-science%2Fsection%2Fcrop-science-and-horticulture&usg=AFQjCNFL6ZfNpGZrpeNZIQOIXQjeuAs09A&sig2=07OSB27EQk2j7p_KkHTRVQ&bvm=bv.123664746,bs.2,d.d2s&cad=rja)

**Learnmore Mwadzingeni^1*^, Hussein Shimelis^1^, Toi J. Tsilo^2^ and Samson Tesfay^1^**

^1^School of Agricultural, Earth and Environmental Sciences, University of KwaZulu-Natal, P/Bag X01, Scottsville 3209, Pietermaritzburg, South Africa, ^2^Agricultural Research Council-Small Grain Institute, Bethlehem 9700, South Africa.

^*^Corresponding author ([mwadzingenil@yahoo.com](mailto:mwadzingenil@yahoo.com)).

| **Table S1 \|List of wheat genotypes used in the study.** | | | |
| --- | --- | --- | --- |
| **Entry code** | **Pedigree/Name** | **Entry Code** | **Pedigree/Name** |
|  | **Genotypes from CIMMYT’s heat nursery** |  | **Genotypes from CIMMYT’s heat nursery** |
| LM01 | ACHTAR*3//KANZ/KS85-8-5/4/MILAN/KAUZ//PRINIA/3/BAV92/5/MILAN/KAUZ//PRINIA/3/BAV92 | LM40 | WBLL1*2/VIVITSI/6/CNDO/R143//ENTE/MEXI_2/3/AEGILOPS SQUARROSA (TAUS)/4/WEAVER/5/2*JANZ |
| LM02 | MILAN/KAUZ//PRINIA/3/BAV92/5/TRAP#1/BOW//VEE#5/SARA/3/ZHE JIANG 4/4/DUCULA | LM41 | C80.1/3*BATAVIA//2*WBLL1/5/REH/HARE//2*BCN/3/CROC_1/AE.SQUARROSA (213)//PGO/4/HUITES |
| LM03 | FRET2*2/4/SNI/TRAP#1/3/KAUZ*2/TRAP//KAUZ/5/ONIX | LM42 | TRCH/5/REH/HARE//2*BCN/3/CROC_1/AE.SQUARROSA (213)//PGO/4/HUITES |
| LM04 | ONIX/4/MILAN/KAUZ//PRINIA/3/BAV92 | LM43 | ROLF07*2/6/PVN//CAR422/ANA/5/BOW/CROW//BUC/PVN/3/YR/4/TRAP#1 |
| LM05 | ACHTAR/4/MILAN/KAUZ//PRINIA/3/BAV92 | LM44 | ROLF07/TUKURU/5/WBLL1*2/4/YACO/PBW65/3/KAUZ*2/TRAP//KAUZ |
| LM06 | CNO79//PF70354/MUS/3/PASTOR/4/BAV92/5/FRET2/KUKUNA//FRET2/6/MILAN/KAUZ//PRINIA/3/BAV92 | LM45 | ROLF07/YANAC//TACUPETO F2001/BRAMBLING |
| LM07 | CMSA04M00297S-040ZTP0Y-040ZTM-040SY-23ZTM-03Y-0B | LM46 | FRET2/KUKUNA//FRET2/3/PARUS/5/FRET2*2/4/SNI/TRAP#1/3/KAUZ*2/TRAP//KAUZ |
| LM08 | SOKOLL*2/TROST | LM47 | FRET2/KUKUNA//FRET2/3/YANAC/4/FRET2/KIRITATI |
| LM09 | SOKOLL*2/ROLF07 | LM48 | FRET2/KUKUNA//FRET2/3/PASTOR//HXL7573/2*BAU/5/FRET2*2/4/SNI/TRAP#1/3/KAUZ*2/TRAP//KAUZ |
| LM10 | GK ARON/AG SECO 7846//2180/4/2*MILAN/KAUZ//PRINIA/3/BAV92 | LM49 | TRCH/SRTU//KACHU |
| LM11 | SW89-5124*2/FASAN/3/ALTAR 84/AE.SQ//2*OPATA | LM50 | HUW234+LR34/PRINIA*2//SNLG |
| LM12 | SOKOLL/ROLF07 | LM51 | HUW234+LR34/PRINIA*2//YANAC |
| LM13 | ROLF07/3/T.DICOCCON PI94625/AE.SQUARROSA (372)//3*PASTOR | LM52 | HUW234+LR34/PRINIA*2//WHEAR |
| LM14 | MILAN/KAUZ//PRINIA/3/BAV92/4/WBLL1*2/KUKUNA | LM53 | HUW234+LR34/PRINIA*2//KIRITATI |
| LM15 | RL6043/4*NAC//PASTOR/3/BAV92/4/ATTILA/BAV92//PASTOR | LM54 | PBW343*2/KUKUNA*2//KITE |
| LM16 | PASTOR*2/BAV92/3/FRET2/KUKUNA//FRET2 | LM55 | PBW343*2/KUKUNA//PARUS/3/PBW343*2/KUKUNA |
| LM17 | ESDA/KKTS | LM56 | PBW343*2/KUKUNA*2//YANAC |
| LM18 | GOUBARA-1/2*SOKOLL | LM57 | PBW343*2/KUKUNA//SRTU/3/PBW343*2/KHVAKI |
| LM19 | SOKOLL*2/4/CHEN/AEGILOPS SQUARROSA (TAUS)//FCT/3/STAR | LM58 | ATTILA*2/PBW65/6/PVN//CAR422/ANA/5/BOW/CROW//BUC/PVN/3/YR/4/TRAP#1/7/ATTILA/2*PASTOR |
| LM20 | PBW343 | LM59 | FRET2/KUKUNA//FRET2/3/WHEAR/4/FRET2/TUKURU//FRET2 |
| LM21 | PRL/2*PASTOR | LM60 | ALD/CEP75630//CEP75234/PT7219/3/BUC/BJY/4/CBRD/5/TNMU/PF85487/6/PBW343*2/KUKUNA/7/CNO79//PF70354/MUS/3/PASTOR/4/BAV92 |
| LM22 | MUNAL #1 |  |  |
| LM23 | QUAIU |  | **Local Checks** |
| LM24 | WBLL1*2/BRAMBLING | LM61 | Check |
| LM25 | WHEAR//2*PRL/2*PASTOR | LM62 | Check |
| LM26 | ATTILA*2/PBW65//TAM200/TUI | LM64 | Check |
| LM27 | YUNMAI 48//2*WBLL1*2/KURUKU | LM65 | Check |
| LM28 | ATTILA/3*BCN//BAV92/3/TILHI/4/SHA7/VEE#5//ARIV92 | LM66 | Check |
| LM29 | PRL/2*PASTOR*2//SKAUZ/BAV92 | LM67 | Check |
| LM30 | C80.1/3*BATAVIA//2*WBLL1/3/ATTILA/3*BCN*2//BAV92/4/WBLL1*2/KURUKU | LM68 | Check |
| LM31 | ATTILA*2/HUITES//FINSI/3/ATTILA*2/PBW65 | LM70 | Check |
| LM32 | ATTILA*2//CHIL/BUC*2/3/KUKUNA |  | **Genotypes from CIMMYT’s drought nursery** |
| LM33 | ATTILA*2/PBW65//KACHU | LM71 | BABAX/3/PRL/SARA//TSI/VEE#5/4/CROC_1/AE.SQUARROSA (224)//2*OPATA |
| LM34 | WBLL1/KUKUNA//TACUPETO F2001/5/WAXWING/4/SNI/TRAP#1/3/KAUZ*2/TRAP//KAUZ | LM72 | BABAX/3/PRL/SARA//TSI/VEE#5/4/WBLL1 |
| LM35 | WBLL1//UP2338*2/VIVITSI | LM73 | BAU/KAUZ//PASTOR |
| LM36 | WBLL1*2/4/SNI/TRAP#1/3/KAUZ*2/TRAP//KAUZ/5/KACHU | LM75 | BUC/MN72253//PASTOR |
| LM37 | KACHU/SAUAL | LM76 | MILAN/KAUZ//PRINIA/3/BABAX |
| LM38 | SAUAL/3/MILAN/S87230//BAV92 | LM77 | CNDO/R143//ENTE/MEXI_2/3/AEGILOPS SQUARROSA (TAUS)/4/WEAVER/5/2*FRAME |
| LM39 | ATTILA/3*BCN//BAV92/3/TILHI/5/BAV92/3/PRL/SARA//TSI/VEE#5/4/CROC_1/AE.SQUARROSA (224)//2*OPATA | LM78 | SW89.5277/BORL95//SKAUZ |

| **Table S1 \| (Continued)** | | | |
| --- | --- | --- | --- |
| **Entry code** | **Pedigree/Name** | **Entry Code** | **Pedigree/Name** |
|  | **Genotypes from CIMMYT’s heat nursery** |  | **Genotypes from CIMMYT’s heat nursery** |
| LM79 | CROC_1/AE.SQUARROSA (205)//BORL95/3/KENNEDY | LM90 | CROC_1/AE.SQUARROSA (205)//BORL95/3/KENNEDY-2 |
| LM80 | CROC_1/AE.SQUARROSA (205)//KAUZ/3/SLVS | LM91 | FRTL/CMH83.2517 |
| LM81 | CROC_1/AE.SQUARROSA (224)//2*OPATA/3/2*RAC655 | LM93 | PASTOR/FLORKWA.1//PASTOR |
| LM82 | HD30/5/CNDO/R143//ENTE/MEXI75/3/AE.SQ/4/2*OCI | LM94 | CROC_1/AE.SQUARROSA (224)//OPATA/3/PASTOR/4/PASTOR*2/OPATA |
| LM83 | PASTOR/3/VEE#5//DOVE/BUC | LM95 | D67.2/P66.270//AE.SQUARROSA (320)/3/CUNNINGHAM |
| LM84 | SRN/AE.SQUARROSA (358)//MILAN/SHA7 | LM96 | ALTAR 84/AE.SQ//2*OPATA/3/PIFED |
| LM85 | SW94.60002/4/KAUZ*2//DOVE/BUC/3/KAUZ/5/SW91-12331 | LM97 | KRICHAUFF/2*PASTOR |
| LM86 | CHAM 6 | LM98 | KABY//2*ALUBUC/BAYA |
| LM87 | KLEIN CHAMACO | LM99 | ALTAR 84/AEGILOPS SQUARROSA (TAUS)//OCI/3/VEE/MJI//2*TUI |
| LM88 | HIDHAB | LM100 | SW89.5277/BORL95//SKAUZ |
| LM89 | DHARWAR DRY |  |  |
| LM1-LM60, genotypes sourced from CIMMYT’s heat nursery; LM61-LM70, local checks; M71-LM100, genotypes sourced from CIMMYT’s drought nursery. | | | |

| **Table S2 \| Means for the number of days to heading, number of days to maturity, number of tillers per plant, plant height (cm), spike length (cm), number of spikelets per spike, number of kernels per spike, thousand seed weight (grams/1000 seeds), grain yield per 30 plant plot (grams) and proline content (µm/g) of 96 wheat genotypes when evaluated under stressed and non-stressed treatments the in four testing environments.** | | | | | | | | | | | | | | | | |
| --- | --- | --- | --- | --- | --- | --- | --- | --- | --- | --- | --- | --- | --- | --- | --- | --- |
| **Entry** | **Traits, water regime and environments** | | | | | | | | | | | | | | | |
|  | **Number of days to 50% heading** | | | | | | | | **Number of days to maturity** | | | | | | | |
|  | **E1** | | **E2** | | **E3** | | **E4** | | **E1** | | **E2** | | **E3** | | **E4** | |
|  | **WR1** | **WR2** | **WR1** | **WR2** | **WR1** | **WR2** | **WR1** | **WR2** | **WR1** | **WR2** | **WR1** | **WR2** | **WR1** | **WR2** | **WR1** | **WR2** |
| LM01 | 41.00 | 41.00 | 46.00 | 46.00 | 42.00 | 42.00 | 50.00 | 48.00 | 95.00 | 89.00 | 110.50 | 106.00 | 82.50 | 90.00 | 97.00 | 104.50 |
| LM02 | 46.00 | 43.50 | 58.50 | 53.50 | 47.00 | 47.00 | 50.00 | 52.00 | 91.00 | 93.00 | 108.00 | 112.50 | 75.00 | 90.00 | 97.00 | 107.00 |
| LM03 | 46.00 | 43.50 | 56.00 | 53.50 | 52.00 | 52.00 | 52.00 | 58.50 | 80.00 | 93.00 | 110.00 | 115.00 | 80.00 | 87.50 | 97.00 | 104.50 |
| LM04 | 48.50 | 51.00 | 63.50 | 61.00 | 52.00 | 52.00 | 61.00 | 63.50 | 93.00 | 93.00 | 115.00 | 115.00 | 95.00 | 97.50 | 104.50 | 114.50 |
| LM05 | 51.00 | 51.00 | 66.00 | 61.00 | 62.00 | 62.00 | 56.50 | 66.00 | 95.00 | 99.00 | 117.00 | 117.00 | 97.50 | 100.00 | 117.00 | 119.50 |
| LM06 | 43.50 | 46.00 | 56.00 | 56.00 | 52.00 | 52.00 | 59.00 | 61.00 | 93.00 | 97.00 | 110.00 | 115.00 | 87.50 | 92.50 | 104.50 | 114.50 |
| LM07 | 51.00 | 48.50 | 61.00 | 58.50 | 57.00 | 57.00 | 58.50 | 68.50 | 91.00 | 95.00 | 115.00 | 114.50 | 97.50 | 97.50 | 109.50 | 117.00 |
| LM08 | 46.00 | 43.50 | 51.00 | 51.00 | 52.00 | 52.00 | 52.00 | 52.00 | 84.50 | 87.00 | 108.00 | 106.00 | 80.00 | 82.50 | 102.00 | 102.00 |
| LM09 | 53.50 | 51.00 | 66.00 | 56.00 | 57.00 | 57.00 | 61.00 | 63.50 | 95.00 | 93.00 | 117.00 | 115.00 | 90.00 | 100.00 | 104.50 | 119.50 |
| LM10 | 51.00 | 51.00 | 58.50 | 61.00 | 57.00 | 57.00 | 63.50 | 66.00 | 93.00 | 95.00 | 115.00 | 117.00 | 97.50 | 97.50 | 104.50 | 117.00 |
| LM11 | 46.00 | 46.00 | 53.50 | 53.50 | 52.00 | 52.00 | 58.50 | 54.00 | 89.00 | 87.00 | 110.00 | 112.50 | 87.50 | 85.00 | 104.50 | 107.00 |
| LM12 | 46.00 | 46.00 | 56.00 | 56.00 | 52.00 | 52.00 | 54.00 | 52.00 | 87.00 | 93.00 | 110.50 | 110.50 | 85.00 | 80.00 | 97.00 | 102.00 |
| LM13 | 48.50 | 46.00 | 61.00 | 58.50 | 52.00 | 52.00 | 61.00 | 56.00 | 91.00 | 90.00 | 112.50 | 112.50 | 87.50 | 87.50 | 104.50 | 109.50 |
| LM14 | 41.00 | 41.00 | 46.00 | 46.00 | 42.00 | 42.00 | 48.00 | 48.00 | 93.00 | 93.00 | 115.00 | 110.50 | 90.00 | 87.50 | 97.00 | 104.50 |
| LM15 | 51.00 | 51.00 | 61.00 | 56.00 | 54.50 | 54.50 | 63.50 | 66.00 | 93.00 | 95.00 | 115.00 | 119.00 | 92.50 | 100.00 | 112.00 | 117.00 |
| LM16 | 46.00 | 48.50 | 58.50 | 56.00 | 54.50 | 52.00 | 54.00 | 58.50 | 87.00 | 89.00 | 115.00 | 112.50 | 85.00 | 87.50 | 99.50 | 104.50 |
| LM17 | 43.50 | 46.00 | 53.50 | 51.00 | 52.00 | 52.00 | 52.00 | 52.00 | 88.50 | 89.00 | 112.50 | 115.00 | 80.00 | 92.50 | 99.50 | 104.50 |
| LM18 | 43.50 | 46.00 | 51.00 | 48.50 | 47.00 | 47.00 | 50.00 | 48.00 | 89.00 | 97.00 | 108.00 | 112.50 | 85.00 | 90.00 | 107.00 | 102.00 |
| LM19 | 51.00 | 51.00 | 53.50 | 56.00 | 57.00 | 57.00 | 61.00 | 61.00 | 95.00 | 97.00 | 115.00 | 119.00 | 95.00 | 100.00 | 112.00 | 119.50 |
| LM20 | 53.50 | 51.00 | 66.00 | 61.00 | 62.00 | 62.00 | 63.50 | 66.00 | 97.00 | 95.00 | 112.50 | 114.50 | 97.50 | 97.50 | 107.89 | 117.00 |
| LM21 | 51.00 | 48.50 | 56.00 | 61.00 | 54.50 | 54.50 | 63.50 | 63.50 | 91.00 | 95.00 | 115.00 | 119.00 | 97.50 | 97.50 | 104.50 | 119.50 |
| LM22 | 51.00 | 51.00 | 55.96 | 61.00 | 57.00 | 57.00 | 61.00 | 61.00 | 93.00 | 95.00 | 114.50 | 114.50 | 95.00 | 100.00 | 102.00 | 107.00 |
| LM23 | 48.50 | 56.00 | 63.50 | 58.50 | 54.50 | 52.00 | 58.50 | 63.50 | 88.50 | 97.00 | 115.00 | 112.50 | 90.00 | 87.50 | 102.00 | 117.00 |
| LM24 | 51.00 | 51.00 | 66.00 | 63.50 | 54.50 | 57.00 | 63.50 | 61.00 | 84.50 | 95.00 | 115.00 | 115.00 | 97.50 | 97.50 | 107.00 | 112.00 |
| LM25 | 51.00 | 46.00 | 61.00 | 61.00 | 54.50 | 57.00 | 58.50 | 63.50 | 91.00 | 97.00 | 110.00 | 112.50 | 92.50 | 95.00 | 107.00 | 117.00 |
| LM26 | 43.50 | 38.50 | 48.50 | 46.00 | 42.00 | 42.00 | 48.00 | 48.00 | 89.00 | 91.00 | 108.00 | 110.00 | 77.50 | 92.50 | 97.00 | 99.50 |
| LM27 | 48.50 | 46.00 | 56.00 | 58.50 | 52.00 | 57.00 | 61.00 | 63.50 | 80.00 | 85.00 | 110.00 | 106.00 | 90.00 | 90.00 | 102.00 | 112.00 |
| LM28 | 48.50 | 48.50 | 61.00 | 63.50 | 54.50 | 52.00 | 63.50 | 58.50 | 87.00 | 89.00 | 112.50 | 117.00 | 95.00 | 97.50 | 107.00 | 117.00 |
| LM29 | 51.00 | 51.00 | 58.50 | 58.50 | 54.50 | 57.00 | 58.50 | 66.00 | 93.00 | 97.00 | 112.50 | 115.00 | 97.50 | 97.50 | 109.50 | 119.50 |
| LM30 | 48.50 | 51.00 | 63.50 | 58.50 | 52.00 | 57.00 | 58.50 | 58.50 | 91.00 | 95.00 | 115.00 | 115.00 | 87.50 | 100.00 | 102.00 | 114.50 |
| LM31 | 43.50 | 48.50 | 56.00 | 56.00 | 52.00 | 52.00 | 56.50 | 61.00 | 89.00 | 93.00 | 112.50 | 112.50 | 77.50 | 85.00 | 107.00 | 117.00 |
| LM32 | 51.00 | 51.00 | 58.50 | 58.50 | 52.00 | 52.00 | 56.50 | 56.00 | 95.00 | 99.00 | 114.50 | 115.00 | 85.00 | 97.50 | 102.00 | 114.50 |
| LM33 | 43.50 | 51.00 | 58.50 | 56.00 | 54.50 | 52.00 | 58.50 | 63.50 | 95.00 | 97.00 | 115.00 | 119.00 | 92.50 | 100.00 | 107.00 | 117.00 |
| LM34 | 41.00 | 46.00 | 51.00 | 51.00 | 47.00 | 47.00 | 54.00 | 54.00 | 89.00 | 87.00 | 108.00 | 110.00 | 80.00 | 92.50 | 102.00 | 104.50 |
| LM35 | 48.50 | 48.50 | 53.50 | 51.00 | 52.00 | 52.00 | 54.00 | 50.00 | 89.00 | 97.00 | 110.50 | 115.00 | 87.50 | 90.00 | 104.50 | 104.50 |
| LM36 | 51.00 | 51.00 | 63.50 | 63.50 | 52.00 | 57.00 | 56.00 | 58.50 | 91.00 | 95.00 | 115.00 | 115.00 | 97.50 | 100.00 | 102.00 | 114.50 |
| LM37 | 51.00 | 43.50 | 56.00 | 56.00 | 52.00 | 52.00 | 54.00 | 58.50 | 91.00 | 89.00 | 115.00 | 115.00 | 87.50 | 97.50 | 104.50 | 119.50 |
| LM38 | 48.50 | 46.00 | 56.00 | 56.00 | 52.00 | 52.00 | 63.50 | 58.50 | 93.00 | 99.00 | 110.00 | 115.00 | 90.00 | 100.00 | 107.00 | 117.00 |
| LM39 | 43.50 | 43.50 | 53.50 | 53.50 | 52.00 | 54.50 | 54.00 | 52.00 | 82.50 | 93.00 | 115.00 | 117.00 | 92.50 | 95.00 | 102.00 | 107.00 |
| LM40 | 43.50 | 46.00 | 53.50 | 53.50 | 52.00 | 52.00 | 54.00 | 54.00 | 80.00 | 87.00 | 108.00 | 110.00 | 82.50 | 80.00 | 102.00 | 104.50 |
| LM41 | 48.50 | 51.00 | 58.50 | 58.50 | 52.00 | 54.50 | 58.50 | 59.00 | 91.00 | 97.00 | 112.50 | 115.00 | 90.00 | 97.50 | 107.00 | 119.50 |
| LM42 | 48.50 | 51.00 | 58.50 | 61.04 | 57.00 | 57.00 | 66.00 | 68.50 | 91.00 | 101.00 | 115.00 | 115.11 | 90.00 | 100.00 | 112.00 | 114.50 |
| LM43 | 53.50 | 51.00 | 63.50 | 61.00 | 62.00 | 62.00 | 63.50 | 63.50 | 95.00 | 97.00 | 112.50 | 119.00 | 100.00 | 100.00 | 107.00 | 119.50 |
| LM44 | 46.00 | 46.00 | 56.00 | 53.50 | 52.00 | 52.00 | 56.50 | 66.00 | 84.50 | 85.00 | 110.00 | 108.00 | 80.00 | 82.50 | 104.50 | 117.00 |
| LM45 | 56.00 | 51.00 | 58.50 | 58.50 | 54.50 | 52.00 | 68.50 | 63.50 | 95.00 | 97.00 | 115.00 | 117.00 | 95.00 | 100.00 | 109.50 | 117.00 |

| **Table S2 \| (Continued)** | | | | | | | | | | | | | | | | |
| --- | --- | --- | --- | --- | --- | --- | --- | --- | --- | --- | --- | --- | --- | --- | --- | --- |
| **Entry** | **Traits, water regime and environments** | | | | | | | | | | | | | | | |
|  | **Number of days to 50% heading** | | | | | | | | **Number of days to maturity** | | | | | | | |
|  | **E1** | | **E2** | | **E3** | | **E4** | | **E1** | | **E2** | | **E3** | | **E4** | |
|  | **WR1** | **WR2** | **WR1** | **WR2** | **WR1** | **WR2** | **WR1** | **WR2** | **WR1** | **WR2** | **WR1** | **WR2** | **WR1** | **WR2** | **WR1** | **WR2** |
| LM46 | 46.00 | 48.50 | 58.50 | 61.00 | 54.50 | 52.00 | 54.00 | 61.00 | 93.00 | 99.00 | 112.50 | 117.00 | 92.50 | 95.00 | 102.00 | 109.50 |
| LM47 | 46.00 | 48.50 | 61.00 | 61.00 | 52.00 | 52.00 | 61.00 | 63.50 | 88.50 | 93.00 | 115.00 | 115.00 | 90.00 | 95.00 | 107.00 | 117.00 |
| LM48 | 48.50 | 51.00 | 61.00 | 63.50 | 52.00 | 57.00 | 61.00 | 61.00 | 87.00 | 93.00 | 112.50 | 117.00 | 87.50 | 95.00 | 104.50 | 109.50 |
| LM49 | 53.50 | 53.50 | 56.00 | 66.00 | 54.50 | 57.00 | 66.00 | 66.00 | 91.00 | 101.00 | 115.00 | 119.00 | 95.00 | 100.00 | 112.00 | 119.50 |
| LM50 | 41.00 | 43.50 | 51.00 | 46.00 | 42.00 | 42.00 | 48.00 | 48.00 | 82.50 | 95.00 | 112.50 | 108.00 | 85.00 | 85.00 | 97.00 | 104.50 |
| LM51 | 38.50 | 41.00 | 48.50 | 46.00 | 42.00 | 42.00 | 50.00 | 52.00 | 85.00 | 89.00 | 106.00 | 108.00 | 82.50 | 87.50 | 99.50 | 104.50 |
| LM52 | 41.00 | 41.00 | 53.50 | 51.00 | 52.00 | 52.00 | 56.50 | 58.50 | 82.50 | 91.00 | 106.00 | 106.00 | 90.00 | 87.50 | 109.50 | 112.00 |
| LM53 | 38.50 | 36.00 | 46.00 | 46.00 | 47.00 | 47.00 | 48.00 | 48.00 | 84.50 | 87.00 | 106.00 | 106.00 | 90.00 | 87.50 | 104.50 | 104.50 |
| LM54 | 48.50 | 48.50 | 63.50 | 63.50 | 52.00 | 57.00 | 71.00 | 66.00 | 87.00 | 93.00 | 115.00 | 115.00 | 92.50 | 100.00 | 114.50 | 114.50 |
| LM55 | 48.50 | 46.00 | 56.00 | 51.00 | 52.00 | 52.00 | 52.00 | 54.00 | 91.00 | 91.00 | 110.00 | 110.00 | 87.50 | 92.50 | 99.50 | 112.00 |
| LM56 | 48.50 | 46.00 | 53.50 | 56.00 | 52.00 | 52.00 | 61.00 | 61.00 | 86.50 | 87.00 | 112.50 | 112.50 | 90.00 | 92.50 | 107.00 | 117.00 |
| LM57 | 46.00 | 46.00 | 56.00 | 56.00 | 52.00 | 54.50 | 56.00 | 66.00 | 85.00 | 89.00 | 108.00 | 110.00 | 90.00 | 90.00 | 104.50 | 114.50 |
| LM58 | 51.00 | 53.50 | 56.00 | 61.00 | 52.00 | 57.00 | 61.00 | 58.50 | 93.00 | 95.00 | 115.00 | 117.00 | 92.50 | 100.00 | 107.00 | 114.50 |
| LM59 | 51.00 | 53.50 | 68.50 | 68.50 | 54.50 | 57.00 | 66.00 | 66.00 | 93.00 | 99.00 | 117.00 | 114.50 | 95.00 | 97.50 | 112.00 | 119.50 |
| LM60 | 48.50 | 48.50 | 58.50 | 58.50 | 52.00 | 52.00 | 58.50 | 66.00 | 80.00 | 87.00 | 115.00 | 115.00 | 97.50 | 95.00 | 112.00 | 119.50 |
| LM61 | 38.50 | 36.00 | 46.00 | 46.00 | 47.00 | 47.00 | 50.00 | 50.00 | 87.00 | 91.00 | 106.00 | 106.00 | 80.00 | 90.00 | 99.50 | 99.50 |
| LM62 | 51.00 | 43.50 | 58.50 | 56.00 | 62.00 | 62.00 | 71.00 | 71.00 | 85.00 | 93.00 | 115.00 | 115.00 | 100.00 | 100.00 | 119.50 | 119.50 |
| LM64 | 33.50 | 33.50 | 46.00 | 46.00 | 47.00 | 47.00 | 52.00 | 48.00 | 93.00 | 93.00 | 110.00 | 110.00 | 87.50 | 90.00 | 107.00 | 102.00 |
| LM65 | 41.00 | 38.50 | 56.00 | 53.50 | 57.00 | 57.00 | 66.00 | 63.50 | 91.00 | 89.00 | 115.00 | 115.00 | 95.00 | 100.00 | 119.50 | 117.00 |
| LM66 | 33.50 | 36.00 | 46.00 | 46.00 | 42.00 | 44.50 | 48.00 | 48.00 | 93.00 | 85.00 | 108.00 | 110.50 | 82.50 | 97.50 | 107.00 | 109.50 |
| LM67 | 33.50 | 33.50 | 46.00 | 46.00 | 47.00 | 47.00 | 48.00 | 48.00 | 89.00 | 91.00 | 108.00 | 112.50 | 90.00 | 92.50 | 99.50 | 104.50 |
| LM68 | 31.00 | 38.50 | 46.00 | 48.50 | 52.00 | 52.00 | 63.50 | 63.50 | 80.00 | 85.00 | 110.50 | 112.50 | 82.50 | 90.00 | 112.00 | 119.50 |
| LM70 | 43.50 | 38.50 | 46.00 | 48.50 | 52.00 | 52.00 | 63.50 | 58.50 | 82.50 | 89.00 | 110.50 | 110.00 | 82.50 | 87.50 | 102.89 | 112.00 |
| LM71 | 53.50 | 51.00 | 56.00 | 56.00 | 52.00 | 52.00 | 54.00 | 56.50 | 80.00 | 93.00 | 115.00 | 112.50 | 82.50 | 92.50 | 102.00 | 109.50 |
| LM72 | 53.50 | 51.00 | 48.50 | 48.50 | 47.00 | 47.00 | 48.00 | 50.00 | 93.00 | 99.00 | 106.00 | 115.00 | 77.50 | 90.00 | 99.50 | 107.00 |
| LM73 | 48.50 | 58.50 | 56.00 | 58.50 | 52.00 | 52.00 | 52.00 | 54.00 | 101.00 | 101.00 | 115.00 | 115.00 | 77.50 | 92.50 | 102.00 | 107.00 |
| LM75 | 61.00 | 63.50 | 56.00 | 56.00 | 52.00 | 52.00 | 52.00 | 54.00 | 97.00 | 105.00 | 115.00 | 112.50 | 80.00 | 85.00 | 97.00 | 112.00 |
| LM76 | 48.50 | 51.00 | 51.00 | 51.00 | 47.00 | 47.00 | 48.00 | 48.00 | 97.00 | 99.00 | 110.50 | 115.00 | 87.50 | 90.00 | 97.00 | 102.00 |
| LM77 | 51.00 | 51.00 | 56.00 | 58.50 | 52.00 | 52.00 | 61.00 | 58.50 | 87.00 | 95.00 | 115.00 | 115.00 | 87.50 | 95.00 | 109.50 | 107.00 |
| LM78 | 53.50 | 56.00 | 56.00 | 56.00 | 52.00 | 52.00 | 54.00 | 56.50 | 99.00 | 101.00 | 110.00 | 115.00 | 90.00 | 92.50 | 102.00 | 102.00 |
| LM79 | 51.00 | 51.00 | 58.50 | 56.00 | 52.00 | 52.00 | 56.50 | 54.00 | 90.50 | 101.00 | 112.50 | 110.50 | 92.50 | 90.00 | 102.00 | 102.00 |
| LM80 | 58.50 | 66.94 | 61.00 | 66.00 | 52.00 | 57.00 | 66.00 | 66.00 | 97.00 | 105.00 | 115.00 | 115.00 | 85.00 | 90.00 | 106.11 | 117.00 |
| LM81 | 56.00 | 56.00 | 61.00 | 63.50 | 57.00 | 52.00 | 61.00 | 63.50 | 97.00 | 99.00 | 115.00 | 115.00 | 92.50 | 100.00 | 104.50 | 112.00 |
| LM82 | 58.50 | 56.00 | 58.50 | 56.00 | 52.00 | 52.00 | 56.50 | 63.50 | 99.00 | 101.00 | 110.50 | 115.00 | 80.00 | 100.00 | 99.50 | 109.50 |
| LM83 | 61.00 | 53.50 | 63.50 | 61.00 | 57.00 | 57.00 | 58.50 | 63.50 | 99.00 | 101.00 | 112.50 | 115.00 | 95.00 | 100.00 | 102.00 | 114.50 |
| LM84 | 61.00 | 63.50 | 63.50 | 66.00 | 54.50 | 57.00 | 61.00 | 63.50 | 103.00 | 105.00 | 115.00 | 119.00 | 95.00 | 100.00 | 107.00 | 114.50 |
| LM85 | 58.50 | 53.50 | 56.00 | 58.50 | 52.00 | 54.50 | 61.00 | 58.50 | 99.00 | 99.00 | 112.50 | 115.00 | 85.00 | 92.50 | 104.50 | 109.50 |
| LM86 | 51.00 | 48.50 | 51.00 | 51.00 | 47.00 | 47.00 | 52.00 | 56.00 | 97.00 | 99.00 | 112.50 | 110.50 | 85.00 | 87.50 | 102.00 | 109.50 |
| LM87 | 48.50 | 46.00 | 48.50 | 46.00 | 47.00 | 47.00 | 48.00 | 50.00 | 91.00 | 103.00 | 108.00 | 108.00 | 80.00 | 92.50 | 99.50 | 107.00 |
| LM88 | 51.00 | 51.00 | 51.00 | 51.00 | 47.00 | 47.00 | 48.00 | 50.00 | 97.00 | 99.00 | 106.00 | 112.50 | 75.00 | 87.50 | 97.00 | 104.50 |
| LM89 | 63.50 | 51.00 | 58.50 | 58.50 | 52.00 | 52.00 | 56.00 | 61.00 | 101.00 | 105.00 | 117.00 | 117.00 | 100.00 | 90.00 | 107.00 | 109.50 |
| LM90 | 53.50 | 51.00 | 56.00 | 61.00 | 52.00 | 52.00 | 52.00 | 56.00 | 95.00 | 95.00 | 112.50 | 115.00 | 80.00 | 90.00 | 99.50 | 107.00 |
| LM91 | 41.00 | 41.00 | 48.50 | 51.00 | 47.00 | 47.00 | 48.00 | 48.00 | 95.00 | 97.00 | 108.00 | 110.50 | 92.50 | 90.00 | 97.00 | 104.50 |
| LM93 | 58.50 | 58.50 | 56.00 | 51.00 | 52.00 | 52.00 | 54.00 | 58.50 | 99.00 | 99.00 | 106.00 | 108.00 | 80.00 | 95.00 | 97.00 | 109.50 |
| LM94 | 51.00 | 48.50 | 58.50 | 56.00 | 52.00 | 52.00 | 58.50 | 58.50 | 90.50 | 101.00 | 110.50 | 115.00 | 92.50 | 95.00 | 99.50 | 104.50 |
| LM95 | 43.50 | 53.50 | 48.50 | 48.50 | 42.00 | 42.00 | 48.00 | 48.00 | 93.00 | 99.00 | 108.00 | 106.00 | 80.00 | 82.50 | 97.00 | 102.00 |
| LM96 | 53.50 | 53.50 | 61.00 | 58.50 | 52.00 | 52.00 | 56.00 | 58.50 | 99.00 | 101.00 | 115.00 | 117.00 | 87.50 | 92.50 | 109.50 | 114.50 |

| **Table S2 \| (Continued)** | | | | | | | | | | | | | | | | | | |
| --- | --- | --- | --- | --- | --- | --- | --- | --- | --- | --- | --- | --- | --- | --- | --- | --- | --- | --- |
| **Entry** | **Traits, water regime and environments** | | | | | | | | | | | | | | | | | |
|  | **Number of days to 50% heading** | | | | | | | | | **Number of days to maturity** | | | | | | | | |
|  | **E1** | | **E2** | | **E3** | | **E4** | | | **E1** | | **E2** | | **E3** | | **E4** | | |
| LM97 | 56.00 | 66.00 | 58.50 | 63.50 | 52.00 | 52.00 | 58.50 | 56.00 | | 101.00 | 105.00 | 112.50 | 119.00 | 90.00 | 95.00 | 112.00 | | 104.50 |
| LM98 | 53.50 | 51.00 | 56.00 | 56.00 | 52.00 | 52.00 | 61.00 | 66.00 | | 93.50 | 93.00 | 106.00 | 115.00 | 82.50 | 87.50 | 102.00 | | 109.50 |
| LM99 | 48.50 | 51.00 | 56.00 | 56.00 | 52.00 | 52.00 | 52.00 | 54.00 | | 84.50 | 93.00 | 106.00 | 112.50 | 87.50 | 90.00 | 97.00 | | 102.00 |
| LM100 | 63.50 | 63.50 | 66.00 | 66.00 | 54.50 | 52.00 | 63.50 | 66.00 | | 105.00 | 103.00 | 117.00 | 117.00 | 97.50 | 97.50 | 104.50 | | 114.50 |
| Mean | 48.63 | 48.69 | 56.26 | 55.92 | 51.87 | 52.29 | 57.03 | 58.30 | | 91.14 | 94.89 | 112.01 | 113.49 | 88.44 | 93.18 | 104.30 | | 110.98 |
| **Entry** | **Number of tillers per plant** | | | | | | | | | **Plant height (cm)** | | | | | | | | |
|  | **E1** | | **E2** | | **E3** | | **E4** | | | **E1** | | **E2** | | **E3** | | **E4** | | |
|  | **WR1** | **WR2** | **WR1** | **WR2** | **WR1** | **WR2** | **WR1** | | **WR2** | **WR1** | **WR2** | **WR1** | **WR2** | **WR1** | **WR2** | **WR1** | **WR2** | |
| LM01 | 3.10 | 3.40 | 3.77 | 4.07 | 2.70 | 3.90 | 3.80 | | 5.42 | 57.38 | 61.56 | 71.75 | 72.50 | 70.90 | 78.15 | 75.00 | 81.95 | |
| LM02 | 3.65 | 3.60 | 4.36 | 4.11 | 2.50 | 3.40 | 3.53 | | 4.47 | 70.75 | 73.62 | 80.44 | 83.50 | 82.99 | 81.24 | 84.25 | 89.55 | |
| LM03 | 3.30 | 4.00 | 4.00 | 4.77 | 3.20 | 4.10 | 4.66 | | 5.27 | 75.50 | 76.26 | 87.75 | 89.94 | 84.40 | 90.97 | 85.00 | 89.15 | |
| LM04 | 3.40 | 4.10 | 4.07 | 4.87 | 3.50 | 3.10 | 2.93 | | 4.71 | 67.50 | 65.87 | 81.81 | 83.75 | 79.51 | 76.73 | 78.90 | 84.35 | |
| LM05 | 3.00 | 5.30 | 3.63 | 6.37 | 4.10 | 4.00 | 2.93 | | 4.57 | 66.34 | 71.11 | 85.44 | 88.75 | 65.81 | 83.12 | 69.55 | 80.55 | |
| LM06 | 3.00 | 4.30 | 3.71 | 5.13 | 2.70 | 3.30 | 4.00 | | 5.15 | 68.75 | 73.04 | 79.12 | 85.25 | 84.23 | 91.37 | 78.55 | 80.50 | |
| LM07 | 3.30 | 4.10 | 4.28 | 4.90 | 3.10 | 4.70 | 4.10 | | 5.70 | 75.56 | 74.50 | 89.88 | 90.50 | 74.25 | 84.19 | 77.85 | 80.95 | |
| LM08 | 3.40 | 3.70 | 4.10 | 4.43 | 3.50 | 4.10 | 3.83 | | 5.13 | 60.73 | 61.88 | 66.00 | 70.69 | 76.34 | 82.76 | 70.81 | 81.20 | |
| LM09 | 3.70 | 6.10 | 4.64 | 7.47 | 2.80 | 4.30 | 4.20 | | 5.43 | 69.62 | 75.06 | 76.37 | 85.37 | 57.39 | 93.56 | 74.00 | 80.20 | |
| LM10 | 3.00 | 4.50 | 3.63 | 5.47 | 3.00 | 3.30 | 2.71 | | 4.38 | 71.12 | 69.87 | 82.00 | 87.96 | 64.55 | 88.09 | 72.70 | 78.95 | |
| LM11 | 3.30 | 3.30 | 4.00 | 4.07 | 2.40 | 4.10 | 3.13 | | 5.51 | 71.88 | 75.75 | 73.12 | 76.25 | 76.72 | 86.62 | 73.65 | 81.10 | |
| LM12 | 3.40 | 3.70 | 4.13 | 4.43 | 3.00 | 4.70 | 4.67 | | 5.86 | 69.12 | 70.86 | 80.25 | 72.79 | 75.49 | 88.51 | 75.85 | 87.55 | |
| LM13 | 3.30 | 5.10 | 4.00 | 6.17 | 3.20 | 4.00 | 3.53 | | 4.93 | 69.50 | 78.31 | 88.50 | 83.25 | 81.17 | 88.75 | 81.10 | 85.60 | |
| LM14 | 3.50 | 3.70 | 4.27 | 4.53 | 3.10 | 3.50 | 4.49 | | 3.60 | 54.56 | 67.19 | 80.25 | 67.87 | 67.03 | 79.69 | 71.03 | 73.25 | |
| LM15 | 3.30 | 4.30 | 4.00 | 5.13 | 2.90 | 3.60 | 4.15 | | 5.42 | 71.38 | 82.37 | 84.69 | 85.00 | 70.12 | 89.91 | 77.60 | 85.95 | |
| LM16 | 2.20 | 3.80 | 2.67 | 4.53 | 2.80 | 4.70 | 4.03 | | 4.73 | 64.88 | 76.06 | 77.21 | 81.81 | 79.66 | 86.92 | 86.55 | 86.10 | |
| LM17 | 3.30 | 4.90 | 3.97 | 6.17 | 2.40 | 3.80 | 3.50 | | 6.40 | 64.72 | 69.00 | 74.69 | 77.44 | 77.69 | 85.10 | 70.40 | 80.90 | |
| LM18 | 3.30 | 3.00 | 3.97 | 3.41 | 4.00 | 3.50 | 3.93 | | 4.90 | 64.00 | 57.25 | 68.25 | 66.25 | 73.92 | 72.28 | 73.44 | 77.70 | |
| LM19 | 3.50 | 4.50 | 4.20 | 5.40 | 3.60 | 3.60 | 5.08 | | 4.57 | 71.12 | 76.87 | 82.62 | 88.75 | 73.76 | 85.44 | 80.00 | 75.30 | |
| LM20 | 2.60 | 4.20 | 3.17 | 5.38 | 2.80 | 2.57 | 2.86 | | 5.14 | 67.50 | 66.50 | 76.50 | 73.50 | 65.27 | 73.22 | 70.46 | 77.70 | |
| LM21 | 3.10 | 4.60 | 3.73 | 5.72 | 2.60 | 3.70 | 3.18 | | 4.91 | 63.66 | 68.67 | 75.44 | 83.12 | 70.42 | 80.11 | 75.80 | 75.50 | |
| LM22 | 4.00 | 4.05 | 5.17 | 5.29 | 2.90 | 4.80 | 4.30 | | 4.77 | 67.31 | 72.25 | 73.87 | 75.81 | 75.00 | 84.81 | 82.25 | 83.35 | |
| LM23 | 4.00 | 7.00 | 4.90 | 8.47 | 3.10 | 4.30 | 3.80 | | 4.57 | 68.62 | 81.50 | 85.50 | 95.44 | 79.99 | 90.82 | 88.59 | 86.80 | |
| LM24 | 3.10 | 3.40 | 3.73 | 4.10 | 3.10 | 3.10 | 3.27 | | 4.13 | 70.50 | 71.44 | 84.25 | 85.12 | 77.44 | 84.64 | 75.70 | 81.35 | |
| LM25 | 2.50 | 4.60 | 3.10 | 5.53 | 2.80 | 4.10 | 3.40 | | 4.53 | 65.38 | 69.94 | 75.00 | 76.44 | 71.62 | 81.69 | 67.85 | 76.80 | |
| LM26 | 3.20 | 4.70 | 3.87 | 5.70 | 2.80 | 3.80 | 3.83 | | 5.27 | 61.94 | 63.69 | 70.00 | 74.12 | 73.88 | 63.94 | 69.15 | 73.95 | |
| LM27 | 3.40 | 3.00 | 4.10 | 3.63 | 2.40 | 4.00 | 3.75 | | 5.23 | 67.19 | 68.25 | 84.12 | 73.75 | 76.72 | 81.73 | 78.35 | 76.40 | |
| LM28 | 2.20 | 3.60 | 2.70 | 4.27 | 3.10 | 3.90 | 4.04 | | 5.04 | 66.31 | 68.69 | 80.12 | 84.99 | 70.63 | 86.64 | 74.96 | 74.95 | |
| LM29 | 3.80 | 4.20 | 4.57 | 5.00 | 3.10 | 3.90 | 3.73 | | 5.53 | 70.81 | 73.06 | 83.19 | 85.19 | 77.28 | 80.25 | 73.85 | 78.30 | |
| LM30 | 2.90 | 3.50 | 3.43 | 4.20 | 2.60 | 3.20 | 2.70 | | 4.70 | 72.60 | 75.20 | 75.87 | 82.81 | 67.29 | 86.97 | 79.70 | 83.25 | |
| LM31 | 3.50 | 4.00 | 4.23 | 4.83 | 2.60 | 2.30 | 3.73 | | 5.84 | 72.44 | 71.75 | 81.50 | 82.31 | 83.36 | 82.48 | 76.00 | 76.80 | |
| LM32 | 2.50 | 4.40 | 2.82 | 6.13 | 2.20 | 3.80 | 3.33 | | 4.13 | 71.54 | 80.44 | 76.62 | 83.25 | 86.81 | 95.49 | 85.10 | 89.50 | |
| LM33 | 3.30 | 3.30 | 4.04 | 3.97 | 3.20 | 2.60 | 3.17 | | 3.73 | 68.70 | 69.50 | 78.62 | 82.25 | 80.36 | 76.12 | 68.18 | 79.55 | |
| LM34 | 2.70 | 3.30 | 3.30 | 3.93 | 2.80 | 4.30 | 3.87 | | 4.40 | 59.88 | 62.75 | 74.87 | 72.12 | 80.66 | 89.31 | 77.21 | 86.15 | |
| LM35 | 3.00 | 5.40 | 3.63 | 6.47 | 3.60 | 3.50 | 3.84 | | 5.83 | 68.84 | 68.50 | 78.50 | 80.06 | 76.37 | 93.61 | 83.70 | 88.35 | |

| **Table S2 \| (Continued)** | | | | | | | | | | | | | | | | |
| --- | --- | --- | --- | --- | --- | --- | --- | --- | --- | --- | --- | --- | --- | --- | --- | --- |
| **Entry** | **Traits, water regime and environments** | | | | | | | | | | | | | | | |
|  | **Number of tillers per plant** | | | | | | | | **Plant height (cm)** | | | | | | | |
|  | **E1** | | **E2** | | **E3** | | **E4** | | **E1** | | **E2** | | **E3** | | **E4** | |
| LM36 | 2.50 | 4.40 | 3.05 | 5.27 | 2.30 | 4.20 | 4.23 | 5.33 | 67.19 | 70.81 | 81.25 | 88.44 | 77.57 | 90.69 | 80.25 | 81.80 |
| LM37 | 1.90 | 3.10 | 2.58 | 3.73 | 2.40 | 3.00 | 2.90 | 3.83 | 63.31 | 66.87 | 67.25 | 69.37 | 75.06 | 83.77 | 74.30 | 70.85 |
| LM38 | 2.60 | 2.20 | 3.28 | 2.67 | 2.40 | 3.20 | 2.84 | 4.23 | 72.19 | 63.25 | 79.25 | 81.00 | 75.66 | 85.64 | 78.20 | 85.70 |
| LM39 | 3.20 | 3.90 | 3.80 | 4.70 | 2.50 | 5.10 | 2.93 | 4.63 | 69.94 | 74.62 | 76.25 | 75.00 | 82.02 | 94.06 | 70.40 | 81.95 |
| LM40 | 2.80 | 3.90 | 3.33 | 4.73 | 3.20 | 3.10 | 3.87 | 6.97 | 69.30 | 73.19 | 75.50 | 72.62 | 80.55 | 91.62 | 75.95 | 76.90 |
| LM41 | 2.60 | 4.10 | 3.10 | 4.90 | 2.10 | 3.80 | 2.87 | 5.01 | 70.99 | 77.50 | 76.12 | 82.06 | 76.87 | 84.85 | 76.40 | 77.72 |
| LM42 | 3.10 | 3.90 | 3.73 | 5.59 | 2.80 | 3.80 | 2.43 | 3.67 | 73.81 | 71.94 | 89.74 | 85.88 | 73.16 | 82.25 | 73.80 | 76.30 |
| LM43 | 3.10 | 4.40 | 3.77 | 5.27 | 2.30 | 5.10 | 2.81 | 5.23 | 71.44 | 78.62 | 86.50 | 94.25 | 73.38 | 92.61 | 76.75 | 85.10 |
| LM44 | 3.00 | 3.00 | 3.60 | 3.60 | 2.80 | 4.10 | 4.30 | 5.12 | 75.37 | 75.00 | 88.96 | 85.31 | 80.44 | 92.97 | 87.30 | 89.80 |
| LM45 | 3.40 | 4.10 | 4.03 | 4.93 | 2.40 | 3.60 | 3.42 | 5.62 | 69.06 | 66.62 | 80.69 | 95.00 | 69.94 | 81.12 | 75.75 | 83.17 |
| LM46 | 3.00 | 4.20 | 3.74 | 5.00 | 2.80 | 4.60 | 3.30 | 4.07 | 62.44 | 66.81 | 78.50 | 84.12 | 72.53 | 82.76 | 74.63 | 82.48 |
| LM47 | 2.90 | 4.30 | 3.50 | 5.20 | 2.80 | 2.90 | 3.50 | 5.37 | 66.28 | 70.12 | 81.19 | 85.25 | 77.03 | 71.00 | 79.37 | 85.85 |
| LM48 | 2.50 | 4.60 | 3.00 | 5.50 | 1.90 | 2.90 | 2.97 | 3.80 | 75.25 | 77.56 | 86.62 | 82.25 | 75.40 | 91.89 | 79.94 | 85.50 |
| LM49 | 2.80 | 4.30 | 3.33 | 5.17 | 2.20 | 3.40 | 2.71 | 3.90 | 72.62 | 82.94 | 82.75 | 89.00 | 75.72 | 89.47 | 79.80 | 83.80 |
| LM50 | 3.00 | 5.10 | 3.60 | 6.13 | 3.20 | 2.60 | 3.40 | 4.34 | 60.94 | 54.19 | 64.15 | 67.00 | 62.87 | 70.91 | 66.40 | 68.30 |
| LM51 | 3.80 | 4.20 | 4.60 | 5.10 | 2.90 | 4.10 | 4.13 | 4.68 | 56.94 | 58.31 | 59.17 | 61.12 | 60.56 | 73.15 | 65.80 | 68.90 |
| LM52 | 3.90 | 4.40 | 4.67 | 5.43 | 2.70 | 4.80 | 3.65 | 6.84 | 63.88 | 60.37 | 65.50 | 65.50 | 69.72 | 79.56 | 62.10 | 72.85 |
| LM53 | 3.70 | 5.60 | 4.47 | 6.57 | 2.90 | 5.30 | 3.65 | 5.98 | 52.31 | 49.19 | 63.69 | 51.00 | 62.69 | 79.34 | 63.00 | 65.20 |
| LM54 | 2.60 | 4.30 | 3.13 | 5.13 | 2.70 | 3.90 | 2.43 | 4.27 | 69.63 | 66.81 | 81.31 | 77.38 | 73.62 | 82.94 | 69.50 | 78.45 |
| LM55 | 3.10 | 4.00 | 3.73 | 4.83 | 2.10 | 3.80 | 4.13 | 5.94 | 62.94 | 62.81 | 70.75 | 66.00 | 75.29 | 85.92 | 77.60 | 79.45 |
| LM56 | 3.30 | 2.80 | 4.20 | 3.33 | 3.40 | 3.60 | 3.17 | 5.13 | 68.56 | 64.50 | 72.50 | 81.69 | 73.06 | 83.22 | 75.70 | 80.25 |
| LM57 | 2.93 | 3.50 | 3.57 | 4.20 | 3.30 | 3.60 | 2.83 | 5.20 | 68.99 | 68.44 | 73.44 | 73.37 | 76.19 | 82.54 | 74.20 | 77.96 |
| LM58 | 2.70 | 4.20 | 3.30 | 4.97 | 2.90 | 2.80 | 3.28 | 4.17 | 74.51 | 79.37 | 78.12 | 84.87 | 77.56 | 81.80 | 74.56 | 85.23 |
| LM59 | 2.80 | 4.40 | 3.78 | 5.50 | 2.90 | 4.10 | 4.46 | 5.37 | 70.25 | 69.06 | 90.08 | 85.87 | 63.82 | 83.31 | 74.25 | 83.10 |
| LM60 | 3.20 | 3.60 | 3.87 | 4.27 | 2.90 | 3.70 | 2.67 | 4.17 | 72.56 | 70.25 | 84.56 | 83.25 | 80.41 | 86.37 | 74.60 | 80.55 |
| LM61 | 2.70 | 2.43 | 3.20 | 3.00 | 2.80 | 3.90 | 4.58 | 4.77 | 47.51 | 47.87 | 63.25 | 61.88 | 67.27 | 74.59 | 61.63 | 64.70 |
| LM62 | 2.10 | 2.80 | 2.50 | 3.40 | 1.91 | 3.50 | 1.47 | 3.60 | 60.75 | 59.87 | 69.75 | 65.75 | 65.33 | 76.65 | 60.40 | 60.30 |
| LM64 | 5.00 | 3.20 | 6.10 | 3.87 | 3.60 | 3.10 | 4.25 | 5.84 | 53.62 | 45.31 | 68.12 | 66.44 | 58.74 | 69.28 | 60.30 | 70.15 |
| LM65 | 2.80 | 2.90 | 3.33 | 4.34 | 3.50 | 4.40 | 4.53 | 7.03 | 52.88 | 48.79 | 69.19 | 60.00 | 68.62 | 74.22 | 58.40 | 72.20 |
| LM66 | 4.50 | 3.90 | 5.47 | 4.73 | 3.60 | 3.60 | 5.37 | 6.08 | 53.27 | 53.75 | 65.06 | 67.75 | 66.08 | 61.02 | 65.04 | 70.40 |
| LM67 | 3.00 | 3.10 | 3.87 | 3.70 | 3.10 | 2.50 | 3.13 | 5.26 | 51.27 | 53.25 | 57.79 | 60.25 | 57.56 | 63.89 | 67.40 | 74.15 |
| LM68 | 2.80 | 2.90 | 3.24 | 3.50 | 3.50 | 3.60 | 4.03 | 7.10 | 49.25 | 53.06 | 57.12 | 64.44 | 69.77 | 69.54 | 60.05 | 71.50 |
| LM70 | 2.70 | 3.20 | 3.27 | 3.87 | 3.20 | 3.00 | 4.01 | 4.70 | 57.50 | 55.06 | 67.50 | 67.37 | 68.07 | 85.65 | 68.00 | 76.05 |
| LM71 | 3.30 | 4.00 | 3.97 | 4.80 | 2.90 | 4.10 | 6.03 | 6.54 | 73.69 | 80.31 | 76.94 | 77.12 | 75.23 | 81.25 | 75.40 | 81.34 |
| LM72 | 2.40 | 2.80 | 3.04 | 3.68 | 3.10 | 3.00 | 3.84 | 4.26 | 75.25 | 82.44 | 73.19 | 81.81 | 82.56 | 82.08 | 79.98 | 77.05 |
| LM73 | 2.67 | 5.00 | 3.27 | 6.60 | 2.40 | 5.00 | 4.40 | 5.77 | 75.31 | 74.62 | 83.00 | 89.12 | 77.48 | 90.83 | 84.93 | 77.40 |
| LM75 | 3.00 | 3.00 | 3.90 | 3.63 | 3.10 | 4.10 | 5.33 | 5.81 | 71.44 | 76.94 | 79.00 | 71.00 | 77.29 | 94.47 | 82.85 | 78.60 |
| LM76 | 2.60 | 3.50 | 3.10 | 4.23 | 2.70 | 4.70 | 4.77 | 5.52 | 68.33 | 70.06 | 75.06 | 73.44 | 66.94 | 74.36 | 72.80 | 78.80 |
| LM77 | 3.00 | 4.00 | 3.67 | 4.87 | 3.00 | 3.00 | 5.12 | 4.68 | 86.50 | 90.37 | 93.06 | 89.37 | 89.56 | 86.89 | 82.50 | 92.20 |
| LM78 | 3.10 | 4.50 | 3.77 | 5.13 | 3.40 | 3.70 | 5.67 | 6.93 | 68.31 | 74.31 | 76.81 | 82.94 | 75.31 | 83.45 | 69.40 | 80.27 |
| LM79 | 2.70 | 4.70 | 3.23 | 5.63 | 2.90 | 3.50 | 3.67 | 5.10 | 83.12 | 83.06 | 89.75 | 83.94 | 82.56 | 87.78 | 88.19 | 90.55 |
| LM80 | 2.70 | 4.33 | 3.72 | 5.62 | 3.70 | 3.30 | 3.23 | 5.03 | 80.39 | 79.50 | 93.25 | 88.06 | 89.62 | 96.00 | 80.65 | 85.20 |
| LM81 | 2.33 | 4.35 | 2.96 | 5.47 | 2.40 | 3.90 | 4.40 | 5.21 | 68.21 | 87.19 | 88.06 | 87.91 | 65.99 | 92.64 | 78.93 | 88.55 |
| LM82 | 3.00 | 4.80 | 3.60 | 5.80 | 3.10 | 4.10 | 5.39 | 4.27 | 68.13 | 75.37 | 73.63 | 73.88 | 71.79 | 79.37 | 71.10 | 74.25 |
| LM83 | 2.20 | 3.90 | 2.63 | 5.34 | 2.70 | 5.80 | 4.59 | 3.98 | 71.23 | 78.19 | 75.38 | 77.62 | 74.54 | 86.91 | 75.45 | 77.90 |
| LM84 | 3.00 | 5.90 | 3.80 | 7.10 | 2.40 | 6.30 | 3.67 | 6.00 | 75.25 | 82.81 | 83.25 | 94.50 | 73.14 | 95.42 | 84.75 | 89.45 |
| LM85 | 3.50 | 4.40 | 4.20 | 5.27 | 2.40 | 4.60 | 4.87 | 5.17 | 68.25 | 73.12 | 77.38 | 83.94 | 74.79 | 81.66 | 70.90 | 80.55 |

| **Table S2 \| (Continued)** | | | | | | | | | | | | | | | | | | | | | |
| --- | --- | --- | --- | --- | --- | --- | --- | --- | --- | --- | --- | --- | --- | --- | --- | --- | --- | --- | --- | --- | --- |
| **Entry** | **Traits, water regime and environments** | | | | | | | | | | | | | | | | | | | | |
|  | **Number of tillers per plant** | | | | | | | | | | **Plant height (cm)** | | | | | | | | | | |
|  | **E1** | | | **E2** | | **E3** | | **E4** | | | **E1** | | | **E2** | | | **E3** | | | **E4** | |
| LM86 | 3.20 | 4.10 | 3.90 | | 4.93 | 3.50 | 5.20 | 4.47 | 5.04 | 63.19 | | | 59.94 | 71.12 | | 67.75 | 64.44 | 61.12 | | 67.70 | 68.50 |
| LM87 | 2.30 | 4.20 | 2.73 | | 5.07 | 3.10 | 2.80 | 3.60 | 4.03 | 66.19 | | | 67.37 | 70.75 | | 71.50 | 78.21 | 65.50 | | 72.25 | 74.00 |
| LM88 | 2.40 | 3.43 | 2.90 | | 4.18 | 2.00 | 1.76 | 5.03 | 5.34 | 63.65 | | | 63.42 | 75.75 | | 74.62 | 71.86 | 70.46 | | 74.50 | 74.90 |
| LM89 | 2.67 | 6.00 | 3.17 | | 7.60 | 2.80 | 3.90 | 3.99 | 5.37 | 69.50 | | | 73.44 | 91.12 | | 86.44 | 72.35 | 89.33 | | 74.40 | 78.15 |
| LM90 | 3.10 | 3.70 | 3.73 | | 4.50 | 3.00 | 3.50 | 4.07 | 4.73 | 79.25 | | | 87.44 | 87.75 | | 92.12 | 86.99 | 97.22 | | 86.85 | 85.95 |
| LM91 | 3.10 | 4.30 | 3.73 | | 5.23 | 3.90 | 4.40 | 4.65 | 6.27 | 62.56 | | | 66.00 | 57.50 | | 67.75 | 62.93 | 71.06 | | 64.25 | 71.80 |
| LM93 | 2.10 | 4.20 | 2.67 | | 5.19 | 2.60 | 3.70 | 4.33 | 4.29 | 68.51 | | | 77.56 | 81.69 | | 75.37 | 82.74 | 97.07 | | 84.60 | 86.60 |
| LM94 | 2.40 | 3.50 | 2.93 | | 4.42 | 3.50 | 3.80 | 5.55 | 6.04 | 68.50 | | | 75.25 | 73.37 | | 79.50 | 75.22 | 81.62 | | 71.20 | 80.70 |
| LM95 | 1.70 | 2.20 | 2.00 | | 2.67 | 2.80 | 2.50 | 3.77 | 3.87 | 55.75 | | | 60.72 | 64.12 | | 59.37 | 64.84 | 69.41 | | 58.90 | 60.65 |
| LM96 | 3.90 | 4.10 | 4.80 | | 4.97 | 2.60 | 3.70 | 5.03 | 5.83 | 78.56 | | | 83.37 | 90.50 | | 93.87 | 79.24 | 86.09 | | 84.82 | 89.75 |
| LM97 | 2.78 | 3.00 | 3.50 | | 4.11 | 2.30 | 3.30 | 3.20 | 4.79 | 69.62 | | | 78.44 | 87.69 | | 82.87 | 72.37 | 85.91 | | 70.10 | 84.45 |
| LM98 | 2.53 | 4.10 | 3.08 | | 4.87 | 2.40 | 3.40 | 4.99 | 5.36 | 72.00 | | | 78.62 | 83.94 | | 87.50 | 72.56 | 88.31 | | 77.50 | 85.30 |
| LM99 | 3.40 | 5.40 | 4.03 | | 6.60 | 2.70 | 5.30 | 4.87 | 5.20 | 68.31 | | | 75.81 | 79.37 | | 76.25 | 77.56 | 76.62 | | 74.30 | 78.70 |
| LM100 | 3.23 | 3.60 | 3.98 | | 4.70 | 2.80 | 4.10 | 3.90 | 3.73 | 72.88 | | | 86.00 | 87.06 | | 96.75 | 76.44 | 91.14 | | 81.60 | 86.35 |
| Mean | 3.02 | 4.03 | 3.68 | | 4.92 | 2.88 | 3.80 | 3.86 | 5.06 | 67.57 | | | 70.48 | 77.66 | | 78.90 | 74.02 | 83.00 | | 74.85 | 79.73 |
| Entry | Spike length (cm) | | | | | | | | | Number of spikelets per spike | | | | | | | | | | | |
|  | E1 | | E2 | | | E3 | | E4 | | E1 | | | | E2 | | | E3 | | | E4 | |
|  | WR1 | WR2 | WR1 | | WR2 | WR1 | WR2 | WR1 | WR2 | WR1 | | WR2 | | WR1 | WR2 | | WR1 | | WR2 | WR1 | WR2 |
| LM01 | 6.71 | 7.11 | 9.23 | | 9.13 | 8.42 | 8.00 | 9.30 | 9.64 | 10.90 | | 10.90 | | 14.60 | 13.60 | | 14.50 | | 14.20 | 16.40 | 17.40 |
| LM02 | 7.30 | 8.55 | 9.71 | | 9.45 | 9.48 | 8.89 | 10.62 | 10.28 | 10.40 | | 13.70 | | 14.40 | 13.70 | | 15.10 | | 14.60 | 19.00 | 17.50 |
| LM03 | 7.44 | 7.89 | 10.03 | | 10.02 | 9.69 | 9.42 | 10.85 | 10.81 | 11.30 | | 12.10 | | 16.20 | 16.00 | | 15.70 | | 16.40 | 20.30 | 19.00 |
| LM04 | 7.86 | 8.05 | 10.85 | | 10.78 | 11.20 | 9.84 | 11.71 | 11.48 | 11.50 | | 12.50 | | 17.30 | 16.10 | | 17.40 | | 15.90 | 21.20 | 20.80 |
| LM05 | 7.47 | 7.63 | 9.35 | | 9.35 | 9.28 | 9.60 | 9.52 | 9.23 | 12.10 | | 13.10 | | 16.70 | 15.80 | | 15.80 | | 18.40 | 17.10 | 18.30 |
| LM06 | 7.51 | 8.19 | 9.92 | | 10.12 | 10.85 | 10.43 | 11.12 | 10.87 | 10.40 | | 11.90 | | 15.10 | 15.70 | | 16.80 | | 16.40 | 18.30 | 18.00 |
| LM07 | 6.73 | 7.25 | 8.65 | | 8.87 | 8.01 | 7.65 | 8.49 | 8.51 | 13.20 | | 13.50 | | 16.80 | 16.50 | | 13.80 | | 17.76 | 17.40 | 16.80 |
| LM08 | 6.69 | 7.00 | 8.36 | | 7.92 | 8.47 | 8.43 | 8.54 | 9.17 | 11.00 | | 11.50 | | 13.66 | 13.40 | | 15.20 | | 14.80 | 16.10 | 17.70 |
| LM09 | 7.39 | 7.74 | 9.57 | | 8.88 | 8.68 | 8.96 | 9.81 | 9.34 | 11.90 | | 11.60 | | 15.40 | 14.10 | | 13.80 | | 15.60 | 17.40 | 16.30 |
| LM10 | 8.09 | 8.75 | 10.66 | | 11.22 | 10.64 | 10.73 | 11.65 | 10.97 | 12.30 | | 12.50 | | 16.30 | 16.50 | | 15.20 | | 18.40 | 19.70 | 17.10 |
| LM11 | 7.71 | 8.46 | 9.91 | | 10.20 | 9.11 | 9.49 | 9.60 | 9.83 | 11.20 | | 13.40 | | 15.60 | 16.70 | | 14.90 | | 16.70 | 17.60 | 19.20 |
| LM12 | 6.68 | 7.51 | 8.95 | | 8.55 | 8.37 | 8.51 | 9.00 | 9.50 | 9.90 | | 11.30 | | 15.60 | 14.30 | | 14.10 | | 15.00 | 17.80 | 18.20 |
| LM13 | 7.02 | 7.70 | 9.55 | | 9.30 | 7.81 | 8.92 | 9.73 | 9.82 | 12.10 | | 12.90 | | 16.80 | 16.30 | | 14.10 | | 16.00 | 18.50 | 18.50 |
| LM14 | 6.45 | 7.22 | 8.95 | | 8.43 | 7.06 | 7.66 | 8.48 | 8.35 | 9.30 | | 11.40 | | 15.10 | 13.20 | | 11.30 | | 13.70 | 15.50 | 14.87 |
| LM15 | 8.08 | 9.33 | 10.01 | | 10.06 | 9.79 | 10.03 | 10.57 | 10.26 | 12.20 | | 16.00 | | 16.40 | 16.20 | | 14.50 | | 16.90 | 19.20 | 18.80 |
| LM16 | 7.85 | 8.47 | 9.48 | | 10.44 | 8.55 | 9.76 | 10.79 | 10.57 | 11.40 | | 12.80 | | 13.50 | 15.00 | | 13.00 | | 15.80 | 17.90 | 17.80 |
| LM17 | 7.72 | 8.16 | 9.28 | | 9.47 | 8.62 | 9.18 | 9.48 | 10.00 | 11.20 | | 12.30 | | 14.70 | 14.60 | | 13.90 | | 15.40 | 16.20 | 16.40 |
| LM18 | 6.96 | 6.75 | 8.70 | | 8.23 | 8.27 | 8.32 | 8.53 | 8.86 | 10.80 | | 10.45 | | 14.10 | 13.30 | | 15.10 | | 13.30 | 16.40 | 17.30 |
| LM19 | 7.12 | 7.74 | 8.76 | | 9.57 | 8.33 | 7.71 | 9.25 | 8.17 | 12.10 | | 13.70 | | 15.50 | 15.90 | | 14.90 | | 14.70 | 17.80 | 14.80 |
| LM20 | 8.08 | 8.41 | 9.74 | | 9.40 | 8.91 | 8.17 | 9.84 | 9.81 | 13.30 | | 13.70 | | 16.80 | 16.10 | | 14.80 | | 13.50 | 18.10 | 19.40 |
| LM21 | 7.16 | 7.55 | 8.70 | | 9.23 | 8.15 | 8.62 | 9.35 | 9.08 | 12.40 | | 13.20 | | 15.50 | 15.80 | | 14.30 | | 16.10 | 18.50 | 17.80 |
| LM22 | 7.13 | 7.35 | 9.26 | | 8.91 | 8.01 | 9.07 | 9.94 | 9.54 | 11.80 | | 12.20 | | 15.30 | 15.00 | | 12.90 | | 15.60 | 18.70 | 18.60 |
| LM23 | 8.40 | 9.47 | 10.05 | | 10.41 | 8.83 | 9.64 | 10.89 | 11.09 | 11.40 | | 13.90 | | 14.80 | 15.20 | | 13.80 | | 16.50 | 19.10 | 19.30 |
| LM24 | 9.15 | 9.03 | 11.12 | | 11.07 | 10.19 | 10.92 | 11.77 | 10.48 | 13.20 | | 14.00 | | 18.50 | 18.00 | | 17.60 | | 19.10 | 20.60 | 19.50 |
| LM25 | 6.88 | 7.35 | 9.20 | | 8.74 | 8.44 | 8.85 | 9.43 | 9.06 | 11.60 | | 12.90 | | 16.40 | 15.20 | | 16.10 | | 17.70 | 17.70 | 18.60 |

| **Table S2 \| (Continued)** | | | | | | | | | | | | | | | | |
| --- | --- | --- | --- | --- | --- | --- | --- | --- | --- | --- | --- | --- | --- | --- | --- | --- |
| **Entry** | **Traits, water regime and environments** | | | | | | | | | | | | | | | |
|  | **Spike length (cm)** | | | | | | | | **Number of spikelets per spike** | | | | | | | |
|  | **E1** | | **E2** | | **E3** | | **E4** | | **E1** | | **E2** | | **E3** | | **E4** | |
|  | **WR1** | **WR2** | **WR1** | **WR2** | **WR1** | **WR2** | **WR1** | **WR2** | **WR1** | **WR2** | **WR1** | **WR2** | **WR1** | **WR2** | **WR1** | **WR2** |
| LM26 | 6.89 | 7.50 | 8.81 | 8.97 | 8.60 | 8.14 | 9.14 | 9.15 | 10.80 | 11.80 | 14.70 | 14.70 | 15.10 | 13.90 | 17.10 | 17.90 |
| LM27 | 6.74 | 6.82 | 8.94 | 8.66 | 7.76 | 8.64 | 10.04 | 8.69 | 12.20 | 12.20 | 16.20 | 15.50 | 14.60 | 17.20 | 20.10 | 17.50 |
| LM28 | 7.87 | 8.52 | 9.82 | 9.52 | 9.50 | 9.15 | 9.80 | 9.80 | 11.30 | 13.40 | 16.20 | 16.70 | 14.40 | 15.50 | 16.90 | 18.50 |
| LM29 | 7.11 | 7.50 | 9.13 | 9.14 | 7.96 | 9.04 | 9.35 | 9.38 | 12.50 | 12.90 | 16.30 | 15.30 | 13.80 | 16.50 | 17.50 | 17.40 |
| LM30 | 7.95 | 8.02 | 10.50 | 9.94 | 9.89 | 10.18 | 10.98 | 10.71 | 13.70 | 13.80 | 17.20 | 16.60 | 16.40 | 19.50 | 19.80 | 20.50 |
| LM31 | 8.14 | 8.10 | 9.99 | 9.47 | 9.93 | 8.84 | 10.61 | 10.53 | 10.50 | 11.28 | 14.20 | 12.80 | 14.50 | 12.40 | 17.30 | 16.70 |
| LM32 | 7.26 | 7.84 | 8.80 | 9.10 | 9.07 | 9.48 | 9.84 | 9.80 | 11.90 | 13.10 | 14.50 | 14.90 | 15.50 | 16.70 | 19.10 | 18.00 |
| LM33 | 7.16 | 7.05 | 8.98 | 9.34 | 8.21 | 8.07 | 9.25 | 9.09 | 12.80 | 12.20 | 19.90 | 17.70 | 15.50 | 16.00 | 19.00 | 18.50 |
| LM34 | 7.01 | 7.10 | 8.86 | 8.39 | 8.57 | 8.61 | 9.50 | 9.17 | 10.30 | 11.52 | 15.40 | 14.20 | 14.70 | 17.10 | 18.20 | 17.80 |
| LM35 | 6.96 | 7.31 | 8.75 | 9.29 | 8.41 | 8.43 | 9.26 | 9.80 | 11.90 | 12.60 | 14.90 | 15.60 | 15.30 | 16.00 | 19.10 | 19.20 |
| LM36 | 7.53 | 7.76 | 9.20 | 9.72 | 8.97 | 10.00 | 10.50 | 10.18 | 10.90 | 12.00 | 15.63 | 15.50 | 14.40 | 16.70 | 17.00 | 17.80 |
| LM37 | 7.66 | 7.83 | 8.71 | 9.09 | 8.85 | 9.05 | 9.93 | 9.23 | 12.50 | 13.20 | 14.70 | 15.30 | 15.90 | 16.90 | 18.10 | 18.50 |
| LM38 | 7.73 | 7.73 | 9.12 | 10.46 | 8.81 | 9.72 | 10.10 | 10.36 | 12.60 | 11.90 | 19.80 | 18.20 | 16.50 | 19.30 | 20.20 | 22.20 |
| LM39 | 7.04 | 7.87 | 8.59 | 9.73 | 8.19 | 8.85 | 9.47 | 9.87 | 11.80 | 11.70 | 15.00 | 17.00 | 13.70 | 16.70 | 18.20 | 19.10 |
| LM40 | 7.72 | 8.62 | 10.04 | 9.47 | 9.35 | 9.84 | 10.51 | 10.11 | 12.30 | 14.30 | 16.10 | 16.40 | 16.70 | 17.40 | 18.70 | 19.10 |
| LM41 | 8.34 | 9.62 | 10.68 | 10.58 | 9.55 | 9.88 | 10.98 | 10.98 | 12.60 | 14.80 | 16.30 | 17.00 | 14.80 | 16.50 | 21.00 | 20.60 |
| LM42 | 7.72 | 8.42 | 9.64 | 9.84 | 8.12 | 9.21 | 9.99 | 9.81 | 13.90 | 15.20 | 18.00 | 18.70 | 15.80 | 17.90 | 19.80 | 20.10 |
| LM43 | 8.40 | 9.37 | 10.95 | 11.00 | 9.71 | 10.20 | 11.53 | 11.31 | 11.30 | 13.30 | 17.30 | 16.50 | 14.30 | 16.90 | 17.90 | 18.20 |
| LM44 | 8.16 | 8.75 | 10.66 | 10.09 | 9.72 | 10.26 | 10.72 | 10.92 | 12.70 | 12.60 | 16.60 | 15.50 | 15.10 | 17.40 | 18.50 | 18.50 |
| LM45 | 8.40 | 8.79 | 10.47 | 10.90 | 10.14 | 9.74 | 11.55 | 11.52 | 12.40 | 12.80 | 15.10 | 16.30 | 15.60 | 16.80 | 18.70 | 19.00 |
| LM46 | 7.76 | 7.80 | 10.24 | 10.45 | 10.24 | 10.10 | 10.26 | 10.49 | 11.60 | 12.00 | 16.20 | 16.70 | 17.00 | 17.60 | 18.00 | 19.10 |
| LM47 | 8.30 | 8.70 | 10.74 | 10.15 | 9.75 | 8.60 | 10.17 | 10.24 | 11.70 | 12.00 | 17.30 | 16.90 | 14.50 | 12.90 | 16.50 | 17.80 |
| LM48 | 8.57 | 8.97 | 11.09 | 10.39 | 10.48 | 9.96 | 11.54 | 11.65 | 11.80 | 12.00 | 16.30 | 14.80 | 14.60 | 14.90 | 19.70 | 19.90 |
| LM49 | 7.69 | 9.14 | 9.77 | 9.97 | 8.91 | 10.01 | 10.76 | 10.01 | 10.60 | 14.40 | 16.90 | 15.70 | 13.40 | 17.30 | 19.70 | 17.50 |
| LM50 | 5.03 | 6.14 | 6.30 | 5.77 | 5.70 | 6.25 | 6.47 | 6.47 | 8.90 | 11.40 | 11.70 | 11.10 | 11.20 | 13.40 | 14.80 | 14.10 |
| LM51 | 5.74 | 5.80 | 7.02 | 6.92 | 6.32 | 7.05 | 7.35 | 7.56 | 9.50 | 9.30 | 11.90 | 11.90 | 11.90 | 13.60 | 14.50 | 15.00 |
| LM52 | 5.55 | 5.91 | 6.81 | 7.14 | 6.32 | 7.15 | 6.98 | 7.77 | 10.00 | 10.58 | 13.10 | 12.40 | 12.00 | 14.80 | 14.20 | 16.00 |
| LM53 | 5.72 | 5.99 | 7.33 | 6.64 | 7.23 | 8.54 | 8.14 | 7.80 | 9.40 | 9.10 | 13.50 | 10.90 | 12.60 | 15.90 | 15.20 | 15.00 |
| LM54 | 5.80 | 6.32 | 7.90 | 7.76 | 6.74 | 8.25 | 7.63 | 7.44 | 12.30 | 14.70 | 18.60 | 18.80 | 15.20 | 19.60 | 17.40 | 19.30 |
| LM55 | 6.20 | 6.86 | 8.68 | 7.88 | 7.18 | 8.04 | 8.39 | 8.81 | 11.10 | 12.60 | 16.00 | 14.70 | 13.40 | 16.30 | 18.10 | 19.20 |
| LM56 | 7.07 | 6.47 | 8.15 | 8.80 | 7.57 | 8.03 | 8.88 | 8.25 | 13.00 | 11.50 | 15.90 | 16.90 | 14.60 | 17.50 | 19.90 | 19.50 |
| LM57 | 7.26 | 7.70 | 9.25 | 8.84 | 8.67 | 9.05 | 10.19 | 9.60 | 12.60 | 12.60 | 16.50 | 15.50 | 15.10 | 18.20 | 20.00 | 19.30 |
| LM58 | 7.85 | 7.84 | 9.39 | 9.18 | 8.86 | 7.60 | 9.32 | 9.89 | 12.50 | 13.20 | 15.30 | 14.80 | 16.10 | 13.10 | 18.00 | 19.90 |
| LM59 | 9.35 | 10.01 | 11.98 | 12.03 | 11.54 | 11.15 | 12.23 | 12.14 | 12.80 | 13.10 | 17.60 | 17.70 | 15.20 | 16.70 | 19.00 | 20.10 |
| LM60 | 7.52 | 8.01 | 8.84 | 8.58 | 9.20 | 8.80 | 9.13 | 9.05 | 11.00 | 12.30 | 13.60 | 14.00 | 13.80 | 14.70 | 16.20 | 16.60 |
| LM61 | 4.37 | 3.95 | 6.42 | 6.23 | 6.25 | 6.85 | 6.85 | 6.79 | 6.60 | 5.30 | 11.10 | 10.70 | 11.90 | 12.60 | 14.30 | 13.90 |
| LM62 | 8.15 | 8.44 | 10.76 | 10.50 | 10.59 | 10.26 | 9.63 | 11.65 | 11.40 | 12.50 | 19.10 | 17.90 | 15.70 | 17.10 | 16.90 | 21.07 |
| LM64 | 4.57 | 4.38 | 6.94 | 7.03 | 6.79 | 6.82 | 7.01 | 7.61 | 5.82 | 5.90 | 10.50 | 11.10 | 11.80 | 11.10 | 12.70 | 13.90 |
| LM65 | 6.34 | 5.82 | 8.40 | 8.92 | 7.19 | 8.05 | 8.98 | 8.42 | 8.20 | 7.70 | 13.40 | 13.80 | 11.60 | 13.90 | 15.80 | 17.00 |
| LM66 | 4.49 | 5.05 | 6.86 | 7.23 | 6.47 | 5.88 | 6.79 | 7.00 | 7.80 | 8.20 | 13.40 | 14.70 | 12.10 | 11.20 | 13.80 | 14.20 |
| LM67 | 4.54 | 5.44 | 6.25 | 6.88 | 7.84 | 5.81 | 8.11 | 9.93 | 7.70 | 8.20 | 12.20 | 10.50 | 14.60 | 11.70 | 15.80 | 17.80 |
| LM68 | 4.98 | 5.66 | 6.81 | 7.85 | 7.85 | 7.23 | 7.05 | 7.51 | 7.50 | 9.10 | 12.30 | 15.20 | 15.80 | 14.70 | 16.67 | 19.70 |
| LM70 | 6.65 | 6.69 | 8.57 | 8.51 | 8.83 | 9.00 | 9.51 | 9.85 | 10.00 | 8.90 | 13.40 | 13.50 | 14.60 | 15.50 | 15.50 | 17.40 |
| LM71 | 9.10 | 9.49 | 9.48 | 9.84 | 8.66 | 8.58 | 9.93 | 9.92 | 12.80 | 14.60 | 15.20 | 13.90 | 12.50 | 14.00 | 17.20 | 17.70 |
| LM72 | 7.99 | 8.69 | 8.86 | 8.74 | 8.88 | 7.95 | 9.86 | 9.08 | 11.60 | 12.80 | 13.70 | 13.40 | 15.20 | 13.20 | 17.90 | 15.80 |
| LM73 | 7.86 | 7.42 | 8.57 | 9.26 | 8.19 | 8.86 | 9.35 | 9.18 | 12.50 | 11.00 | 13.80 | 14.40 | 13.60 | 15.80 | 18.10 | 18.30 |

| **Table S2 \| (Continued)** | | | | | | | | | | | | | | | | | | | | | | | | | | | | | |
| --- | --- | --- | --- | --- | --- | --- | --- | --- | --- | --- | --- | --- | --- | --- | --- | --- | --- | --- | --- | --- | --- | --- | --- | --- | --- | --- | --- | --- | --- |
| **Entry** | **Traits, water regime and environments** | | | | | | | | | | | | | | | | | | | | | | | | | | | | |
|  | **Spike length (cm)** | | | | | | | | | | | | | | **Number of spikelets per spike** | | | | | | | | | | | | | | |
|  | **E1** | | **E2** | | | | **E3** | | | **E4** | | | | | **E1** | | | **E2** | | | | **E3** | | | | **E4** | | | |
|  | **WR1** | **WR2** | **WR1** | | **WR2** | | **WR1** | | **WR2** | **WR1** | | **WR2** | | | **WR1** | **WR2** | | **WR1** | | **WR2** | | **WR1** | | **WR2** | | **WR1** | | | **WR2** |
| LM75 | 7.89 | 8.58 | 9.23 | | 8.98 | | 8.86 | | 9.06 | 9.83 | | 9.19 | | | 12.50 | 13.70 | | 15.30 | | 14.00 | | 14.40 | | 16.70 | | 18.70 | | | 16.40 |
| LM76 | 7.26 | 7.73 | 9.52 | | 9.38 | | 8.19 | | 9.23 | 9.70 | | 9.66 | | | 9.88 | 11.30 | | 14.90 | | 13.10 | | 12.80 | | 15.60 | | 16.10 | | | 17.80 |
| LM77 | 8.14 | 8.59 | 9.63 | | 9.36 | | 8.76 | | 8.13 | 9.85 | | 10.30 | | | 12.00 | 13.90 | | 15.40 | | 13.70 | | 13.90 | | 12.40 | | 16.50 | | | 17.70 |
| LM78 | 6.04 | 6.94 | 7.00 | | 7.47 | | 6.43 | | 6.60 | 7.60 | | 7.68 | | | 11.80 | 13.00 | | 14.60 | | 14.80 | | 13.60 | | 13.40 | | 18.90 | | | 17.40 |
| LM79 | 8.00 | 8.49 | 9.30 | | 9.46 | | 8.53 | | 8.03 | 10.29 | | 10.31 | | | 11.50 | 13.50 | | 14.80 | | 14.10 | | 13.80 | | 12.80 | | 18.40 | | | 17.50 |
| LM80 | 7.88 | 8.83 | 8.48 | | 8.77 | | 8.21 | | 8.61 | 8.76 | | 9.09 | | | 13.70 | 17.30 | | 15.40 | | 15.20 | | 15.40 | | 17.00 | | 16.60 | | | 17.70 |
| LM81 | 8.49 | 8.96 | 10.48 | | 10.12 | | 9.54 | | 9.92 | 10.64 | | 10.47 | | | 13.10 | 14.10 | | 16.20 | | 15.90 | | 15.00 | | 17.20 | | 20.00 | | | 18.30 |
| LM82 | 8.11 | 8.35 | 9.25 | | 8.93 | | 8.29 | | 8.56 | 9.73 | | 9.00 | | | 13.30 | 15.20 | | 15.80 | | 15.00 | | 14.90 | | 16.70 | | 18.50 | | | 17.30 |
| LM83 | 8.56 | 8.84 | 9.30 | | 9.67 | | 8.20 | | 9.41 | 10.17 | | 9.22 | | | 14.20 | 14.90 | | 15.70 | | 16.00 | | 14.70 | | 17.20 | | 19.60 | | | 17.50 |
| LM84 | 8.49 | 9.01 | 9.32 | | 9.72 | | 9.25 | | 9.16 | 9.61 | | 9.87 | | | 13.80 | 15.30 | | 15.50 | | 15.80 | | 14.60 | | 17.30 | | 17.70 | | | 18.40 |
| LM85 | 7.95 | 7.94 | 8.68 | | 9.53 | | 8.01 | | 8.43 | 9.74 | | 9.77 | | | 13.40 | 13.48 | | 14.80 | | 16.30 | | 13.00 | | 14.60 | | 18.90 | | | 18.30 |
| LM86 | 7.44 | 7.24 | 9.38 | | 9.01 | | 7.77 | | 8.23 | 9.61 | | 9.87 | | | 11.40 | 11.10 | | 14.10 | | 14.30 | | 13.60 | | 14.40 | | 17.40 | | | 18.60 |
| LM87 | 6.90 | 7.51 | 9.20 | | 8.15 | | 8.13 | | 7.45 | 9.32 | | 8.79 | | | 10.10 | 11.20 | | 15.00 | | 13.50 | | 14.90 | | 10.70 | | 18.10 | | | 16.27 |
| LM88 | 7.33 | 6.89 | 8.57 | | 8.24 | | 7.91 | | 7.34 | 9.38 | | 9.20 | | | 11.10 | 10.20 | | 13.80 | | 13.80 | | 14.10 | | 11.70 | | 17.60 | | | 17.40 |
| LM89 | 7.28 | 8.29 | 9.35 | | 9.21 | | 7.98 | | 8.46 | 9.24 | | 8.79 | | | 12.20 | 13.90 | | 15.70 | | 15.00 | | 12.50 | | 15.30 | | 18.00 | | | 17.40 |
| LM90 | 8.29 | 8.56 | 9.74 | | 9.93 | | 9.05 | | 8.78 | 10.23 | | 10.11 | | | 10.60 | 12.60 | | 15.30 | | 14.80 | | 14.30 | | 14.30 | | 17.00 | | | 17.00 |
| LM91 | 6.56 | 6.84 | 7.08 | | 7.70 | | 6.57 | | 7.46 | 8.07 | | 7.65 | | | 9.70 | 11.10 | | 12.00 | | 13.20 | | 11.00 | | 13.60 | | 17.50 | | | 16.00 |
| LM93 | 8.62 | 8.57 | 9.76 | | 9.50 | | 10.00 | | 10.51 | 10.65 | | 10.83 | | | 11.60 | 11.40 | | 14.50 | | 14.00 | | 14.50 | | 16.90 | | 17.30 | | | 17.50 |
| LM94 | 5.95 | 6.24 | 7.10 | | 7.55 | | 6.52 | | 6.82 | 7.75 | | 7.96 | | | 12.40 | 11.68 | | 14.40 | | 12.90 | | 13.40 | | 14.50 | | 16.70 | | | 17.00 |
| LM95 | 5.80 | 6.27 | 8.39 | | 7.78 | | 6.84 | | 6.50 | 7.27 | | 7.34 | | | 8.90 | 10.20 | | 14.40 | | 13.40 | | 11.60 | | 12.50 | | 14.40 | | | 14.30 |
| LM96 | 7.03 | 8.09 | 8.31 | | 8.00 | | 7.98 | | 7.11 | 8.24 | | 8.77 | | | 12.00 | 14.00 | | 15.30 | | 15.00 | | 16.10 | | 13.00 | | 17.50 | | | 18.50 |
| LM97 | 9.13 | 9.28 | 10.59 | | 11.03 | | 8.91 | | 9.83 | 10.70 | | 10.88 | | | 13.80 | 15.30 | | 17.60 | | 16.60 | | 15.20 | | 16.70 | | 17.50 | | | 19.30 |
| LM98 | 8.86 | 9.07 | 10.11 | | 10.47 | | 9.58 | | 9.30 | 11.13 | | 10.95 | | | 13.10 | 14.30 | | 14.90 | | 14.70 | | 14.40 | | 15.40 | | 18.10 | | | 18.50 |
| LM99 | 7.28 | 7.78 | 8.99 | | 8.47 | | 7.81 | | 7.59 | 9.28 | | 8.77 | | | 11.40 | 12.80 | | 14.80 | | 14.40 | | 12.20 | | 12.50 | | 17.10 | | | 16.10 |
| LM100 | 8.74 | 9.58 | 10.49 | | 9.93 | | 8.92 | | 9.93 | 10.92 | | 10.72 | | | 11.20 | 14.90 | | 15.80 | | 15.80 | | 13.90 | | 15.10 | | 18.00 | | | 17.40 |
| **Mean** | **7.33** | **7.75** | **9.14** | | **9.15** | | **8.55** | | **8.71** | **9.58** | | **9.53** | | | **11.45** | **12.39** | | **15.31** | | **15.00** | | **14.36** | | **15.49** | | **17.74** | | | **17.85** |
| **Entry** | **Number of kernels per spike** | | | | | | | | | | | | | **Thousand seed weight (grams/1000 seeds)** | | | | | | | | | | | | | | | |
|  | **E1** | | **E2** | | | **E3** | | | | | **E4** | | | **E1** | | | | | **E2** | | | | **E3** | | | | **E4** | | |
|  | **WR1** | **WR2** | **WR1** | **WR2** | | **WR1** | | **WR2** | | | **WR1** | | **WR2** | **WR1** | | | **WR2** | | **WR1** | | **WR2** | | **WR1** | | **WR2** | | **WR1** | **WR2** | |
| LM01 | 22.80 | 26.20 | 41.00 | 44.40 | | 28.20 | | 31.00 | | | 44.00 | | 49.90 | 27.82 | | | 25.24 | | 38.00 | | 37.00 | | 26.33 | | 33.67 | | 30.33 | 35.83 | |
| LM02 | 23.90 | 37.70 | 42.10 | 55.90 | | 33.70 | | 33.60 | | | 52.00 | | 46.00 | 33.40 | | | 33.43 | | 34.00 | | 42.00 | | 13.75 | | 34.58 | | 31.75 | 37.50 | |
| LM03 | 24.40 | 27.70 | 42.60 | 45.90 | | 28.40 | | 33.40 | | | 48.90 | | 45.60 | 24.29 | | | 37.67 | | 40.00 | | 43.00 | | 21.50 | | 42.83 | | 26.50 | 36.42 | |
| LM04 | 29.00 | 29.00 | 47.20 | 47.20 | | 35.20 | | 36.70 | | | 46.20 | | 51.30 | 30.23 | | | 34.86 | | 35.00 | | 38.00 | | 35.08 | | 41.33 | | 33.42 | 31.83 | |
| LM05 | 28.80 | 34.70 | 47.00 | 52.90 | | 18.80 | | 45.20 | | | 46.70 | | 52.50 | 26.99 | | | 35.26 | | 34.00 | | 35.00 | | 40.50 | | 37.33 | | 28.50 | 29.75 | |
| LM06 | 22.60 | 26.10 | 40.80 | 44.30 | | 37.90 | | 37.40 | | | 46.40 | | 51.40 | 31.65 | | | 39.93 | | 38.00 | | 33.00 | | 31.83 | | 42.50 | | 27.67 | 36.50 | |
| LM07 | 28.50 | 32.00 | 46.70 | 50.20 | | 19.40 | | 42.28 | | | 39.80 | | 38.30 | 29.45 | | | 33.75 | | 31.00 | | 35.00 | | 31.75 | | 32.23 | | 27.17 | 27.25 | |
| LM08 | 24.10 | 24.70 | 42.30 | 42.90 | | 28.30 | | 32.70 | | | 37.90 | | 42.90 | 26.58 | | | 29.63 | | 39.00 | | 41.00 | | 20.92 | | 30.58 | | 24.25 | 34.25 | |
| LM09 | 22.70 | 23.40 | 40.90 | 41.60 | | 18.50 | | 33.90 | | | 35.00 | | 36.40 | 33.74 | | | 34.61 | | 31.00 | | 47.00 | | 34.17 | | 38.08 | | 30.00 | 31.08 | |
| LM10 | 25.70 | 29.10 | 43.90 | 47.30 | | 27.20 | | 41.30 | | | 48.70 | | 43.80 | 31.81 | | | 30.10 | | 35.00 | | 45.00 | | 46.00 | | 34.83 | | 28.17 | 32.33 | |
| LM11 | 24.50 | 34.20 | 42.70 | 52.40 | | 22.40 | | 36.00 | | | 35.90 | | 47.10 | 27.15 | | | 27.94 | | 37.00 | | 34.00 | | 24.75 | | 32.33 | | 26.75 | 29.83 | |
| LM12 | 18.40 | 23.30 | 36.60 | 41.50 | | 24.10 | | 27.40 | | | 30.80 | | 33.60 | 29.57 | | | 37.21 | | 33.00 | | 41.00 | | 21.08 | | 40.75 | | 30.83 | 40.33 | |

| **Table S2 \| (Continued)** | | | | | | | | |  | | | | | | | |
| --- | --- | --- | --- | --- | --- | --- | --- | --- | --- | --- | --- | --- | --- | --- | --- | --- |
| **Entry** | **Number of kernels per spike** | | | | | | | | **Thousand seed weight (grams/1000 seeds)** | | | | | | | |
|  | **E1** | | **E2** | | **E3** | | **E4** | | **E1** | | **E2** | | **E3** | | **E4** | |
|  | **WR1** | **WR2** | **WR1** | **WR2** | **WR1** | **WR2** | **WR1** | **WR2** | **WR1** | **WR2** | **WR1** | **WR2** | **WR1** | **WR2** | **WR1** | **WR2** |
| LM13 | 26.40 | 31.10 | 44.60 | 49.30 | 31.30 | 35.80 | 38.50 | 48.10 | 27.64 | 28.99 | 31.47 | 38.00 | 28.75 | 32.42 | 29.58 | 31.33 |
| LM14 | 22.30 | 27.40 | 40.50 | 45.60 | 17.00 | 28.30 | 36.90 | 33.92 | 30.04 | 29.40 | 39.00 | 37.00 | 29.17 | 32.67 | 30.08 | 36.30 |
| LM15 | 28.30 | 38.00 | 46.50 | 56.20 | 26.90 | 43.00 | 48.80 | 46.40 | 25.05 | 37.21 | 39.00 | 35.00 | 28.08 | 40.33 | 29.17 | 33.33 |
| LM16 | 22.10 | 27.80 | 40.30 | 46.00 | 25.30 | 35.00 | 45.50 | 40.70 | 29.26 | 32.08 | 43.47 | 38.00 | 32.00 | 32.58 | 28.42 | 33.58 |
| LM17 | 28.50 | 31.30 | 46.70 | 49.50 | 25.10 | 33.00 | 41.00 | 40.30 | 25.92 | 32.03 | 33.00 | 39.00 | 27.08 | 34.58 | 25.67 | 35.50 |
| LM18 | 24.70 | 20.00 | 42.90 | 36.20 | 27.30 | 23.60 | 39.10 | 37.30 | 27.12 | 29.25 | 39.00 | 38.00 | 24.17 | 32.25 | 30.00 | 34.42 |
| LM19 | 19.40 | 23.40 | 37.60 | 41.60 | 12.10 | 22.10 | 30.50 | 27.90 | 39.48 | 36.52 | 40.00 | 43.00 | 43.27 | 44.42 | 30.25 | 36.17 |
| LM20 | 19.50 | 22.20 | 37.70 | 40.40 | 2.00 | 21.47 | 30.70 | 35.20 | 37.17 | 41.12 | 35.00 | 34.00 | 40.89 | 43.17 | 35.33 | 35.08 |
| LM21 | 27.10 | 31.50 | 45.30 | 49.70 | 26.40 | 35.60 | 42.20 | 42.30 | 29.31 | 37.57 | 37.00 | 45.00 | 39.83 | 42.42 | 24.08 | 34.83 |
| LM22 | 21.80 | 27.80 | 42.80 | 46.00 | 23.30 | 35.70 | 40.90 | 45.40 | 28.56 | 35.72 | 37.47 | 37.00 | 37.08 | 41.25 | 26.83 | 31.50 |
| LM23 | 22.20 | 24.50 | 40.40 | 42.70 | 27.60 | 33.30 | 47.90 | 44.00 | 27.31 | 44.75 | 35.00 | 38.00 | 38.42 | 35.42 | 27.92 | 35.83 |
| LM24 | 27.40 | 32.00 | 45.60 | 50.20 | 23.90 | 40.10 | 50.50 | 45.80 | 21.39 | 35.67 | 38.00 | 35.00 | 44.33 | 38.75 | 25.67 | 29.00 |
| LM25 | 21.90 | 32.90 | 40.10 | 51.10 | 25.10 | 35.30 | 40.10 | 57.10 | 30.10 | 36.61 | 31.00 | 33.47 | 32.08 | 38.75 | 25.17 | 29.58 |
| LM26 | 22.40 | 26.30 | 40.60 | 44.50 | 29.60 | 28.20 | 46.00 | 45.10 | 24.44 | 27.81 | 30.00 | 37.00 | 19.58 | 33.17 | 29.08 | 33.25 |
| LM27 | 29.80 | 29.30 | 48.00 | 47.50 | 29.30 | 39.40 | 43.90 | 40.90 | 23.01 | 31.01 | 38.00 | 41.00 | 36.58 | 32.00 | 30.25 | 30.25 |
| LM28 | 23.50 | 30.20 | 41.70 | 48.40 | 20.00 | 29.40 | 35.60 | 45.10 | 30.95 | 34.62 | 42.00 | 40.00 | 38.94 | 43.00 | 36.53 | 34.92 |
| LM29 | 27.90 | 29.30 | 46.10 | 47.50 | 30.80 | 36.60 | 44.70 | 42.00 | 30.80 | 38.79 | 38.00 | 39.00 | 41.00 | 38.17 | 29.00 | 32.83 |
| LM30 | 39.40 | 32.90 | 57.60 | 51.10 | 21.10 | 42.90 | 43.90 | 59.20 | 24.46 | 33.97 | 35.00 | 39.00 | 29.33 | 41.08 | 29.92 | 37.33 |
| LM31 | 22.60 | 26.35 | 40.80 | 31.00 | 29.80 | 25.50 | 37.70 | 40.30 | 33.03 | 40.88 | 46.00 | 43.00 | 22.17 | 50.56 | 31.25 | 36.83 |
| LM32 | 24.00 | 32.10 | 42.20 | 50.30 | 35.40 | 40.60 | 47.70 | 46.50 | 34.27 | 37.98 | 35.00 | 32.00 | 24.50 | 34.00 | 31.83 | 34.58 |
| LM33 | 25.00 | 25.60 | 43.20 | 43.80 | 27.20 | 31.50 | 41.20 | 47.10 | 25.06 | 33.28 | 40.53 | 36.00 | 25.75 | 25.58 | 25.50 | 31.00 |
| LM34 | 21.00 | 21.45 | 39.20 | 29.30 | 27.00 | 33.00 | 41.60 | 39.40 | 28.84 | 30.80 | 33.00 | 35.00 | 26.17 | 40.33 | 25.67 | 33.17 |
| LM35 | 26.40 | 28.70 | 44.60 | 46.90 | 33.00 | 35.40 | 45.80 | 47.70 | 28.26 | 29.92 | 36.00 | 38.00 | 32.33 | 34.67 | 27.25 | 34.67 |
| LM36 | 24.20 | 28.80 | 42.40 | 47.00 | 32.80 | 37.00 | 39.60 | 41.70 | 30.13 | 35.40 | 37.00 | 40.00 | 35.67 | 38.92 | 30.67 | 33.08 |
| LM37 | 24.80 | 28.45 | 43.00 | 43.30 | 29.60 | 35.10 | 38.50 | 38.30 | 33.30 | 34.96 | 38.00 | 40.00 | 34.00 | 38.00 | 32.50 | 38.58 |
| LM38 | 34.70 | 32.20 | 52.90 | 41.40 | 36.50 | 44.90 | 52.90 | 56.00 | 26.61 | 30.97 | 34.00 | 39.00 | 32.58 | 39.25 | 33.58 | 37.00 |
| LM39 | 25.10 | 27.20 | 43.30 | 45.40 | 27.90 | 38.20 | 47.10 | 46.40 | 22.74 | 31.87 | 31.00 | 27.47 | 34.58 | 37.33 | 30.17 | 36.17 |
| LM40 | 27.20 | 29.10 | 45.40 | 47.30 | 32.70 | 36.20 | 39.70 | 47.50 | 24.90 | 34.46 | 35.00 | 43.00 | 21.27 | 30.08 | 27.33 | 35.08 |
| LM41 | 29.80 | 31.60 | 48.00 | 49.80 | 21.20 | 38.00 | 54.70 | 52.80 | 25.88 | 35.31 | 35.00 | 37.00 | 20.75 | 34.08 | 28.67 | 34.08 |
| LM42 | 23.70 | 28.20 | 41.90 | 55.00 | 17.00 | 35.80 | 45.00 | 45.60 | 23.77 | 28.03 | 30.00 | 32.53 | 23.67 | 37.25 | 26.00 | 23.83 |
| LM43 | 20.00 | 26.50 | 38.20 | 44.70 | 12.80 | 31.50 | 36.10 | 39.90 | 33.88 | 38.57 | 38.00 | 42.00 | 39.17 | 45.67 | 30.83 | 34.83 |
| LM44 | 33.60 | 35.30 | 51.80 | 53.50 | 35.00 | 41.60 | 47.70 | 51.50 | 21.56 | 24.13 | 35.00 | 36.00 | 23.50 | 27.25 | 27.42 | 28.33 |
| LM45 | 24.40 | 27.60 | 42.60 | 45.80 | 25.70 | 36.60 | 42.20 | 42.00 | 37.50 | 41.58 | 36.00 | 46.00 | 36.67 | 42.67 | 29.83 | 36.83 |
| LM46 | 24.30 | 27.60 | 42.50 | 45.80 | 23.20 | 37.20 | 35.70 | 41.40 | 31.68 | 38.34 | 43.00 | 44.00 | 40.08 | 35.42 | 29.17 | 29.75 |
| LM47 | 24.70 | 24.50 | 42.90 | 42.70 | 24.40 | 22.80 | 36.20 | 36.00 | 29.16 | 38.97 | 35.00 | 36.00 | 40.17 | 41.83 | 32.58 | 36.25 |
| LM48 | 23.80 | 24.60 | 42.00 | 42.80 | 26.90 | 23.50 | 41.80 | 49.40 | 32.64 | 35.88 | 37.00 | 38.53 | 36.58 | 41.50 | 29.83 | 29.75 |
| LM49 | 23.10 | 24.30 | 41.30 | 42.50 | 19.20 | 33.00 | 39.00 | 35.30 | 27.59 | 35.93 | 37.00 | 38.00 | 40.17 | 45.08 | 31.00 | 27.83 |
| LM50 | 16.40 | 25.80 | 34.60 | 44.00 | 20.70 | 28.00 | 36.90 | 33.10 | 25.04 | 31.04 | 33.00 | 36.00 | 31.67 | 30.92 | 31.42 | 31.75 |
| LM51 | 19.20 | 21.70 | 37.40 | 39.90 | 22.30 | 24.70 | 32.30 | 31.00 | 24.83 | 27.83 | 34.00 | 41.00 | 28.00 | 39.42 | 33.25 | 35.25 |
| LM52 | 19.40 | 25.60 | 37.60 | 43.80 | 17.80 | 29.00 | 28.20 | 25.70 | 21.22 | 30.80 | 36.00 | 44.00 | 32.08 | 40.17 | 30.58 | 37.58 |
| LM53 | 18.60 | 19.70 | 36.80 | 37.90 | 19.05 | 31.40 | 34.10 | 29.30 | 25.53 | 27.75 | 39.00 | 32.53 | 28.67 | 33.50 | 30.42 | 36.67 |
| LM54 | 23.10 | 29.60 | 41.30 | 47.80 | 29.90 | 35.30 | 34.90 | 37.10 | 34.94 | 39.54 | 35.00 | 36.00 | 41.67 | 40.92 | 31.25 | 36.58 |
| LM55 | 25.40 | 29.20 | 43.60 | 47.40 | 25.40 | 35.40 | 47.40 | 50.80 | 28.96 | 27.01 | 37.47 | 35.00 | 33.75 | 32.67 | 28.67 | 29.33 |
| LM56 | 27.60 | 25.30 | 45.80 | 43.50 | 30.50 | 36.90 | 41.60 | 42.90 | 26.22 | 27.54 | 32.00 | 35.00 | 30.33 | 32.58 | 28.92 | 31.17 |
| LM57 | 26.00 | 32.20 | 44.20 | 50.40 | 32.00 | 42.30 | 48.50 | 48.50 | 27.93 | 30.14 | 34.00 | 37.00 | 31.67 | 33.00 | 27.67 | 29.42 |
| LM58 | 25.20 | 29.20 | 43.40 | 47.40 | 33.80 | 29.10 | 43.80 | 46.30 | 25.90 | 30.37 | 33.00 | 35.00 | 24.33 | 32.42 | 27.75 | 30.75 |
| LM59 | 23.10 | 22.70 | 41.30 | 40.90 | 22.10 | 32.70 | 41.40 | 41.20 | 28.27 | 36.38 | 29.47 | 32.00 | 42.58 | 41.17 | 29.00 | 27.17 |

| **Table S2 \| (Continued)** | | | | | | | | |  | | | | | | | |
| --- | --- | --- | --- | --- | --- | --- | --- | --- | --- | --- | --- | --- | --- | --- | --- | --- |
| **Entry** | **Number of kernels per spike** | | | | | | | | **Thousand seed weight (grams/1000 seeds)** | | | | | | | |
|  | **E1** | | **E2** | | **E3** | | **E4** | | **E1** | | **E2** | | **E3** | | **E4** | |
|  | **WR1** | **WR2** | **WR1** | **WR2** | **WR1** | **WR2** | **WR1** | **WR2** | **WR1** | **WR2** | **WR1** | **WR2** | **WR1** | **WR2** | **WR1** | **WR2** |
| LM60 | 26.60 | 30.20 | 44.80 | 48.40 | 28.00 | 28.70 | 34.10 | 35.10 | 22.91 | 29.57 | 38.00 | 40.00 | 32.42 | 40.92 | 32.67 | 34.67 |
| LM61 | 11.30 | 9.10 | 29.50 | 27.30 | 20.60 | 19.40 | 17.48 | 27.40 | 25.37 | 19.46 | 35.00 | 32.00 | 22.75 | 36.50 | 33.25 | 32.75 |
| LM62 | 30.90 | 30.30 | 49.10 | 48.50 | 26.70 | 33.50 | 22.10 | 46.72 | 21.87 | 30.05 | 33.47 | 39.00 | 27.33 | 34.67 | 25.92 | 19.13 |
| LM64 | 13.70 | 12.30 | 31.90 | 27.90 | 19.30 | 16.80 | 28.80 | 29.30 | 28.51 | 23.93 | 38.00 | 38.00 | 28.33 | 27.58 | 27.75 | 37.50 |
| LM65 | 19.80 | 21.20 | 38.00 | 39.40 | 21.80 | 28.20 | 37.40 | 40.90 | 20.44 | 23.60 | 31.00 | 30.00 | 28.25 | 33.75 | 25.00 | 25.42 |
| LM66 | 15.30 | 19.80 | 33.50 | 38.00 | 22.70 | 22.40 | 27.50 | 32.50 | 30.06 | 27.07 | 36.00 | 33.00 | 25.58 | 31.25 | 33.17 | 35.33 |
| LM67 | 15.10 | 19.90 | 33.30 | 41.40 | 22.50 | 14.80 | 40.10 | 46.70 | 28.21 | 28.45 | 34.00 | 37.00 | 35.27 | 34.39 | 27.83 | 30.92 |
| LM68 | 17.70 | 23.40 | 35.90 | 41.60 | 30.80 | 30.80 | 36.52 | 49.90 | 21.24 | 23.48 | 24.00 | 29.00 | 17.50 | 21.50 | 21.47 | 25.58 |
| LM70 | 21.50 | 22.10 | 39.70 | 40.30 | 35.40 | 41.20 | 33.30 | 47.30 | 23.65 | 24.73 | 33.00 | 34.00 | 18.08 | 26.08 | 23.83 | 27.42 |
| LM71 | 26.40 | 29.20 | 44.60 | 47.40 | 24.30 | 29.10 | 41.10 | 41.80 | 23.34 | 27.75 | 38.00 | 38.00 | 28.67 | 36.75 | 27.75 | 28.92 |
| LM72 | 21.70 | 25.00 | 39.90 | 43.20 | 26.50 | 24.70 | 39.70 | 38.90 | 31.21 | 28.14 | 34.00 | 38.00 | 18.83 | 35.23 | 25.08 | 31.50 |
| LM73 | 31.40 | 26.33 | 49.60 | 39.40 | 26.00 | 32.70 | 42.60 | 49.10 | 30.77 | 34.65 | 36.00 | 38.00 | 20.67 | 38.33 | 28.33 | 31.75 |
| LM75 | 23.10 | 27.40 | 41.30 | 45.60 | 29.20 | 34.50 | 44.60 | 35.90 | 37.05 | 40.93 | 33.00 | 40.00 | 21.67 | 30.75 | 26.25 | 34.58 |
| LM76 | 21.55 | 26.00 | 28.60 | 44.20 | 28.40 | 34.70 | 39.20 | 45.40 | 40.01 | 39.37 | 38.00 | 45.00 | 29.75 | 33.00 | 29.50 | 34.83 |
| LM77 | 23.80 | 27.00 | 42.00 | 40.40 | 28.60 | 24.30 | 32.50 | 42.30 | 29.42 | 45.07 | 41.00 | 45.47 | 38.58 | 48.25 | 38.17 | 42.92 |
| LM78 | 13.40 | 14.40 | 31.60 | 30.20 | 22.50 | 20.50 | 37.20 | 37.40 | 33.29 | 34.94 | 39.00 | 42.00 | 32.08 | 41.58 | 33.50 | 34.08 |
| LM79 | 17.90 | 24.10 | 36.10 | 42.30 | 21.30 | 22.20 | 40.20 | 33.40 | 32.14 | 41.73 | 43.00 | 42.00 | 36.58 | 45.58 | 31.37 | 39.67 |
| LM80 | 28.20 | 42.30 | 46.40 | 60.50 | 34.80 | 39.60 | 38.70 | 45.00 | 23.84 | 37.51 | 35.00 | 40.00 | 30.42 | 34.50 | 28.92 | 37.25 |
| LM81 | 23.20 | 26.60 | 41.40 | 44.80 | 22.80 | 36.80 | 44.10 | 42.90 | 31.69 | 32.11 | 39.00 | 43.00 | 42.92 | 48.00 | 31.33 | 31.25 |
| LM82 | 27.50 | 32.30 | 45.70 | 50.50 | 29.40 | 44.70 | 50.60 | 46.10 | 25.48 | 28.82 | 29.00 | 31.00 | 16.42 | 29.67 | 22.58 | 27.17 |
| LM83 | 25.20 | 32.10 | 43.40 | 50.30 | 15.40 | 40.00 | 47.10 | 47.00 | 26.10 | 34.55 | 29.00 | 33.00 | 37.92 | 41.25 | 25.33 | 31.17 |
| LM84 | 26.20 | 28.90 | 44.40 | 47.10 | 25.70 | 35.40 | 38.50 | 42.00 | 29.59 | 34.49 | 28.00 | 27.00 | 29.00 | 31.08 | 23.25 | 22.67 |
| LM85 | 26.50 | 31.95 | 44.70 | 33.80 | 29.30 | 33.60 | 41.40 | 43.00 | 32.26 | 33.33 | 35.00 | 40.53 | 29.92 | 33.33 | 25.83 | 33.17 |
| LM86 | 22.70 | 24.60 | 40.60 | 42.80 | 28.60 | 25.20 | 41.50 | 44.70 | 25.59 | 22.93 | 28.53 | 33.00 | 19.92 | 29.42 | 24.58 | 28.50 |
| LM87 | 17.20 | 22.60 | 35.40 | 40.80 | 29.50 | 17.10 | 47.60 | 43.32 | 32.35 | 30.41 | 34.00 | 34.00 | 23.00 | 32.33 | 28.25 | 33.47 |
| LM88 | 19.90 | 20.80 | 38.10 | 39.00 | 29.80 | 20.20 | 44.40 | 41.60 | 32.39 | 29.26 | 22.00 | 32.00 | 17.17 | 26.42 | 24.67 | 31.92 |
| LM89 | 22.40 | 32.80 | 40.60 | 51.00 | 21.00 | 31.50 | 41.00 | 43.10 | 24.71 | 25.05 | 27.00 | 30.00 | 31.00 | 24.00 | 24.08 | 25.42 |
| LM90 | 17.30 | 23.30 | 35.50 | 41.50 | 28.10 | 25.90 | 40.80 | 38.00 | 40.85 | 37.68 | 47.00 | 43.47 | 26.75 | 53.67 | 31.83 | 45.17 |
| LM91 | 18.60 | 20.70 | 36.80 | 38.90 | 16.80 | 22.00 | 39.70 | 37.40 | 33.87 | 34.46 | 29.00 | 35.00 | 32.42 | 35.75 | 28.50 | 35.25 |
| LM93 | 23.20 | 19.90 | 41.40 | 36.20 | 19.40 | 46.10 | 36.80 | 39.00 | 38.58 | 38.63 | 38.00 | 36.00 | 27.42 | 40.00 | 29.83 | 36.17 |
| LM94 | 18.10 | 11.50 | 36.30 | 22.50 | 19.80 | 20.80 | 31.40 | 32.40 | 24.85 | 39.94 | 37.00 | 43.00 | 43.17 | 42.75 | 26.00 | 36.00 |
| LM95 | 20.30 | 20.00 | 38.50 | 38.20 | 22.10 | 24.30 | 36.30 | 38.90 | 30.30 | 33.23 | 34.00 | 31.00 | 27.33 | 35.17 | 23.08 | 26.42 |
| LM96 | 22.60 | 28.30 | 40.80 | 46.50 | 35.80 | 23.00 | 42.70 | 46.40 | 30.11 | 37.75 | 35.00 | 36.00 | 21.08 | 39.17 | 25.75 | 29.83 |
| LM97 | 24.00 | 29.70 | 42.20 | 47.90 | 35.30 | 40.87 | 41.40 | 52.60 | 39.53 | 40.05 | 33.00 | 31.00 | 25.92 | 32.92 | 27.17 | 30.25 |
| LM98 | 27.70 | 36.50 | 45.90 | 54.70 | 26.00 | 35.50 | 40.40 | 41.30 | 18.32 | 28.27 | 34.00 | 37.00 | 20.67 | 30.83 | 24.33 | 30.25 |
| LM99 | 24.50 | 26.70 | 42.70 | 44.90 | 25.40 | 28.30 | 38.80 | 34.60 | 20.96 | 29.72 | 31.00 | 30.53 | 19.83 | 25.25 | 24.83 | 29.25 |
| LM100 | 22.90 | 32.30 | 41.10 | 50.50 | 25.30 | 38.50 | 45.00 | 43.00 | 32.34 | 35.84 | 35.00 | 41.00 | 46.50 | 45.83 | 32.17 | 32.42 |
| Mean | 23.60 | 26.97 | 41.71 | 44.47 | 25.75 | 32.29 | 40.43 | 42.38 | 28.72 | 33.08 | 35.26 | 37.42 | 30.25 | 36.47 | 28.66 | 32.76 |

| **Table S2 \| (Continued)** | | | | | | | | | | | | | | | | | | | | | | | |
| --- | --- | --- | --- | --- | --- | --- | --- | --- | --- | --- | --- | --- | --- | --- | --- | --- | --- | --- | --- | --- | --- | --- | --- |
| **Entry** | **Traits, water regime and environments** | | | | | | | | | | | | | | | | | | | | | | |
|  | **Grain yield per 30 plant plot (grams)** | | | | | | | | | | | **Proline content (µm/g)** | | | | | | | | | | | |
|  | **E1** | | | **E2** | | | | **E3** | | | | **E4** | | | | **E1** | | | | **E2** | | | |
|  | **WR1** | **WR2** | | **WR1** | | **WR2** | | **WR1** | | **WR2** | | **WR1** | | **WR2** | | **WR1** | | **WR2** | | **WR1** | | **WR2** | |
| LM01 | 59.20 | 67.70 | | 177.60 | | 200.70 | | 61.80 | | 121.80 | | 150.50 | | 290.20 | | 44.61 | | 32.58 | | 262.77 | | 3.57 | |
| LM02 | 87.00 | 136.30 | | 191.70 | | 290.50 | | 34.70 | | 122.30 | | 174.40 | | 230.90 | | 92.31 | | 51.29 | | 608.98 | | 21.72 | |
| LM03 | 58.70 | 124.80 | | 204.50 | | 282.00 | | 57.70 | | 172.80 | | 182.90 | | 261.50 | | 90.86 | | 38.88 | | 227.74 | | 1.56 | |
| LM04 | 91.80 | 124.10 | | 200.80 | | 262.70 | | 127.50 | | 150.40 | | 135.50 | | 231.60 | | 51.94 | | 23.88 | | 135.61 | | 2.29 | |
| LM05 | 70.50 | 194.30 | | 175.10 | | 349.00 | | 99.10 | | 202.50 | | 116.60 | | 213.50 | | 124.84 | | 41.39 | | 222.20 | | 0.25 | |
| LM06 | 64.20 | 134.50 | | 172.40 | | 224.40 | | 96.80 | | 155.90 | | 152.70 | | 290.40 | | 67.09 | | 30.58 | | 552.10 | | 20.93 | |
| LM07 | 78.20 | 132.50 | | 184.00 | | 257.80 | | 55.90 | | 217.80 | | 135.00 | | 178.30 | | 86.07 | | 42.56 | | 282.57 | | 11.54 | |
| LM08 | 65.30 | 79.20 | | 202.90 | | 232.30 | | 62.20 | | 122.60 | | 108.30 | | 225.90 | | 43.94 | | 38.15 | | 358.43 | | 20.70 | |
| LM09 | 86.20 | 146.40 | | 176.60 | | 439.00 | | 51.60 | | 167.70 | | 130.60 | | 185.90 | | 78.88 | | 17.51 | | 150.65 | | 2.13 | |
| LM10 | 73.30 | 119.20 | | 168.00 | | 350.10 | | 106.20 | | 142.80 | | 111.60 | | 180.10 | | 89.17 | | 24.46 | | 272.16 | | 15.10 | |
| LM11 | 65.90 | 94.50 | | 189.20 | | 215.80 | | 37.10 | | 142.00 | | 89.50 | | 232.30 | | 89.41 | | 42.05 | | 304.45 | | 6.06 | |
| LM12 | 54.10 | 96.20 | | 149.40 | | 225.90 | | 45.30 | | 156.70 | | 132.20 | | 237.90 | | 70.81 | | 44.86 | | 174.74 | | 17.68 | |
| LM13 | 72.00 | 138.00 | | 152.90 | | 351.60 | | 86.90 | | 138.90 | | 120.70 | | 222.50 | | 36.54 | | 43.53 | | 60.71 | | 0.00 | |
| LM14 | 70.40 | 90.10 | | 201.60 | | 232.80 | | 45.40 | | 97.40 | | 151.20 | | 133.70 | | 47.03 | | 23.12 | | 244.55 | | 34.10 | |
| LM15 | 70.60 | 182.80 | | 219.90 | | 303.20 | | 68.90 | | 189.10 | | 180.50 | | 251.80 | | 96.28 | | 38.73 | | 519.11 | | 11.11 | |
| LM16 | 42.60 | 103.20 | | 117.60 | | 239.90 | | 69.40 | | 159.80 | | 156.20 | | 194.90 | | 86.77 | | 39.98 | | 168.86 | | 3.96 | |
| LM17 | 73.40 | 146.00 | | 183.50 | | 357.80 | | 46.90 | | 129.10 | | 110.70 | | 274.90 | | 70.59 | | 28.60 | | 374.88 | | 47.63 | |
| LM18 | 65.70 | 52.60 | | 198.20 | | 142.00 | | 83.20 | | 80.40 | | 136.00 | | 188.80 | | 27.00 | | 25.10 | | 244.15 | | 40.33 | |
| LM19 | 80.60 | 122.60 | | 191.70 | | 288.50 | | 54.20 | | 109.10 | | 139.60 | | 135.60 | | 76.13 | | 51.83 | | 222.09 | | 8.98 | |
| LM20 | 55.10 | 114.60 | | 126.30 | | 221.40 | | 4.60 | | 80.30 | | 93.40 | | 188.90 | | 78.08 | | 34.32 | | 303.27 | | 16.46 | |
| LM21 | 73.80 | 167.90 | | 187.50 | | 381.10 | | 79.10 | | 163.60 | | 97.60 | | 219.40 | | 51.11 | | 60.26 | | 129.12 | | 17.58 | |
| LM22 | 73.80 | 121.90 | | 277.70 | | 266.10 | | 78.00 | | 213.20 | | 143.80 | | 203.20 | | 110.33 | | 42.66 | | 319.45 | | 9.34 | |
| LM23 | 68.10 | | 229.40 | | 197.20 | | 416.90 | | 98.50 | | 170.30 | | 151.50 | | 216.90 | | 115.83 | | 21.86 | | 318.36 | | 17.29 |
| LM24 | 54.50 | | 116.10 | | 192.30 | | 216.40 | | 95.10 | | 141.40 | | 132.60 | | 164.60 | | 80.73 | | 35.45 | | 121.59 | | 16.06 |
| LM25 | 45.60 | | 166.40 | | 113.60 | | 288.60 | | 54.70 | | 170.00 | | 102.30 | | 228.90 | | 14.66 | | 38.17 | | 58.08 | | 16.14 |
| LM26 | 54.10 | | 102.70 | | 143.30 | | 277.70 | | 47.60 | | 124.70 | | 153.50 | | 237.30 | | 67.42 | | 62.57 | | 173.84 | | 9.23 |
| LM27 | 66.80 | | 81.40 | | 213.20 | | 216.20 | | 76.80 | | 152.60 | | 150.80 | | 199.00 | | 81.45 | | 29.25 | | 84.39 | | 22.42 |
| LM28 | 48.00 | | 114.60 | | 141.20 | | 251.20 | | 65.40 | | 149.40 | | 119.30 | | 239.90 | | 70.09 | | 29.52 | | 136.13 | | 8.79 |
| LM29 | 97.90 | | 144.90 | | 239.70 | | 284.50 | | 116.50 | | 160.60 | | 144.70 | | 230.00 | | 53.46 | | 71.36 | | 82.32 | | 22.07 |
| LM30 | 82.40 | | 117.20 | | 209.10 | | 251.40 | | 52.70 | | 169.00 | | 106.60 | | 309.20 | | 104.42 | | 35.11 | | 375.13 | | 0.00 |
| LM31 | 78.10 | | 130.30 | | 238.10 | | 199.20 | | 53.60 | | 108.80 | | 132.30 | | 261.60 | | 79.40 | | 28.00 | | 305.78 | | 29.83 |
| LM32 | 61.10 | | 158.00 | | 125.20 | | 287.80 | | 56.80 | | 157.70 | | 150.90 | | 200.30 | | 73.08 | | 36.04 | | 101.27 | | 8.00 |
| LM33 | 62.60 | | 84.30 | | 249.10 | | 187.00 | | 67.50 | | 69.70 | | 98.90 | | 163.00 | | 76.44 | | 35.86 | | 259.32 | | 16.63 |
| LM34 | 48.90 | | 83.60 | | 124.30 | | 140.40 | | 59.00 | | 172.30 | | 123.90 | | 171.80 | | 59.60 | | 40.41 | | 224.68 | | 13.11 |
| LM35 | 66.90 | | 139.20 | | 176.10 | | 346.60 | | 117.20 | | 128.90 | | 141.10 | | 290.10 | | 92.64 | | 40.88 | | 261.16 | | 0.00 |
| LM36 | 53.10 | | 132.30 | | 140.80 | | 295.00 | | 81.00 | | 182.80 | | 151.90 | | 220.50 | | 57.99 | | 37.78 | | 113.28 | | 7.87 |
| LM37 | 47.90 | | 93.40 | | 129.60 | | 195.90 | | 72.50 | | 120.20 | | 106.90 | | 169.50 | | 74.37 | | 24.37 | | 308.19 | | 0.00 |
| LM38 | 70.80 | | 67.50 | | 176.30 | | 135.90 | | 84.70 | | 168.90 | | 150.60 | | 262.10 | | 71.05 | | 27.29 | | 187.25 | | 19.26 |
| LM39 | 54.50 | | 99.30 | | 152.60 | | 171.60 | | 71.50 | | 216.90 | | 128.30 | | 232.20 | | 76.59 | | 22.58 | | 90.76 | | 29.04 |
| LM40 | 56.90 | | 128.30 | | 158.70 | | 302.00 | | 67.30 | | 98.70 | | 124.70 | | 344.10 | | 73.45 | | 26.04 | | 258.92 | | 13.98 |
| LM41 | 58.90 | | 140.10 | | 157.80 | | 270.10 | | 26.60 | | 160.20 | | 134.80 | | 266.90 | | 72.64 | | 34.66 | | 689.73 | | 29.87 |
| LM42 | 51.60 | | 96.30 | | 141.90 | | 298.60 | | 33.40 | | 156.20 | | 83.90 | | 122.40 | | 38.41 | | 41.28 | | 171.91 | | 9.84 |
| LM43 | 57.70 | | 134.80 | | 164.50 | | 296.50 | | 33.70 | | 216.90 | | 91.30 | | 217.10 | | 82.70 | | 23.47 | | 171.99 | | 7.28 |
| LM44 | 64.00 | | 76.50 | | 193.00 | | 203.20 | | 68.80 | | 141.20 | | 169.40 | | 229.10 | | 70.14 | | 32.15 | | 125.78 | | 6.96 |
| LM45 | 84.10 | | 140.40 | | 183.10 | | 308.00 | | 66.70 | | 167.30 | | 128.00 | | 258.60 | | 37.57 | | 27.73 | | 220.73 | | 8.82 |
| LM46 | 69.60 | | 133.30 | | 203.40 | | 302.10 | | 77.20 | | 180.50 | | 102.90 | | 152.00 | | 61.18 | | 29.88 | | 248.93 | | 0.00 |
| LM47 | 67.50 | | 125.00 | | 160.70 | | 238.30 | | 82.80 | | 87.50 | | 124.90 | | 209.70 | | 91.20 | | 50.56 | | 270.36 | | 28.87 |
| LM48 | 56.70 | | 120.50 | | 139.20 | | 297.40 | | 56.60 | | 85.30 | | 114.90 | | 166.30 | | 57.62 | | 31.41 | | 94.17 | | 4.10 |
| LM49 | 53.50 | | 112.90 | | 151.20 | | 251.80 | | 56.50 | | 152.30 | | 97.90 | | 116.00 | | 36.48 | | 36.79 | | 86.98 | | 27.57 |

| **Table S2 \| (Continued)** | | | | | | | | | | | | | | |
| --- | --- | --- | --- | --- | --- | --- | --- | --- | --- | --- | --- | --- | --- | --- |
| **Entry** | **Traits, water regime and environments** | | | | | | | | | | | | | |
|  | **Grain yield per 30 plant plot (grams)** | | | | | | **Proline content (µm/g)** | | | | | | | |
|  | **E1** | | **E2** | | **E3** | | **E4** | | **E1** | | | **E2** | | |
|  | **WR1** | **WR2** | **WR1** | **WR2** | **WR1** | **WR2** | **WR1** | **WR2** | **WR1** | **WR2** | | **WR1** | | **WR2** |
| LM50 | 36.60 | 121.40 | 122.70 | 287.80 | 68.40 | 68.30 | 114.70 | 137.10 | 26.44 | 42.19 | | 132.70 | | 17.50 |
| LM51 | 54.30 | 77.40 | 175.50 | 246.10 | 52.50 | 119.40 | 140.20 | 151.20 | 42.31 | 29.30 | | 213.56 | | 28.22 |
| LM52 | 48.00 | 104.00 | 191.80 | 314.20 | 41.90 | 173.00 | 94.80 | 201.40 | 78.65 | 37.71 | | 207.52 | | 17.79 |
| LM53 | 52.30 | 91.90 | 192.00 | 255.60 | 43.00 | 182.60 | 112.50 | 191.70 | 51.39 | 23.13 | | 139.66 | | 13.01 |
| LM54 | 62.40 | 148.60 | 137.10 | 259.90 | 101.50 | 172.70 | 79.70 | 173.90 | 59.17 | 41.97 | | 208.10 | | 11.89 |
| LM55 | 67.70 | 94.10 | 176.40 | 240.90 | 54.50 | 135.80 | 168.50 | 267.50 | 36.59 | 25.43 | | 148.47 | | 23.02 |
| LM56 | 69.60 | 58.40 | 187.70 | 154.60 | 95.30 | 129.90 | 113.30 | 215.40 | 64.55 | 26.99 | | 148.72 | | 19.25 |
| LM57 | 61.70 | 103.40 | 156.90 | 234.90 | 101.20 | 150.50 | 117.30 | 221.80 | 74.25 | 22.80 | | 155.53 | | 4.10 |
| LM58 | 51.80 | 111.40 | 139.10 | 244.80 | 74.00 | 88.60 | 124.30 | 176.90 | 84.35 | 44.30 | | 288.69 | | 4.79 |
| LM59 | 53.40 | 109.50 | 137.80 | 226.30 | 81.20 | 174.80 | 160.50 | 179.10 | 76.99 | 54.52 | | 362.27 | | 13.99 |
| LM60 | 58.20 | 96.50 | 196.80 | 247.40 | 76.20 | 129.60 | 91.60 | 151.70 | 69.19 | 35.44 | | 191.41 | | 18.19 |
| LM61 | 24.40 | 12.90 | 99.20 | 79.20 | 38.00 | 81.50 | 39.50 | 126.40 | 81.45 | 30.15 | | 90.52 | | 2.90 |
| LM62 | 42.90 | 76.30 | 140.00 | 193.10 | 26.40 | 130.30 | 25.60 | 98.10 | 74.03 | 37.29 | | 256.11 | | 16.00 |
| LM64 | 58.40 | 28.50 | 222.10 | 124.00 | 62.10 | 44.20 | 101.00 | 191.20 | 83.41 | 29.49 | | 362.18 | | 7.62 |
| LM65 | 34.20 | 44.30 | 118.50 | 156.70 | 62.50 | 125.30 | 136.90 | 214.00 | 90.02 | 28.15 | | 102.22 | | 19.00 |
| LM66 | 62.50 | 67.20 | 197.20 | 180.30 | 61.90 | 80.60 | 153.60 | 209.10 | 108.22 | 26.15 | | 313.91 | | 5.86 |
| LM67 | 39.20 | 51.70 | 134.50 | 148.50 | 100.60 | 61.20 | 108.10 | 230.90 | 30.98 | 24.42 | | 184.23 | | 4.46 |
| LM68 | 31.90 | 47.60 | 79.40 | 126.60 | 56.10 | 73.40 | 76.30 | 275.80 | 81.94 | 19.92 | | 387.82 | | 10.20 |
| LM70 | 42.10 | 57.20 | 127.40 | 162.00 | 66.30 | 96.30 | 93.20 | 196.90 | 57.14 | 38.76 | | 411.59 | | 2.06 |
| LM71 | 55.40 | 99.10 | 205.60 | 261.40 | 58.70 | 130.10 | 206.30 | 237.10 | 105.74 | 61.32 | | 89.00 | | 24.49 |
| LM72 | 47.70 | 59.80 | 121.90 | 181.00 | 45.40 | 84.60 | 114.30 | 154.10 | 72.18 | 44.04 | | 360.11 | | 12.94 |
| LM73 | 75.60 | 137.00 | 174.40 | 296.50 | 38.80 | 180.30 | 158.40 | 269.10 | 60.51 | 33.47 | | 214.26 | | 34.44 |
| LM75 | 77.00 | 106.30 | 158.70 | 214.90 | 54.40 | 128.10 | 187.20 | 214.00 | 57.85 | 47.98 | | 379.90 | | 11.50 |
| LM76 | 58.40 | 108.40 | 95.20 | 254.20 | 63.40 | 165.30 | 165.30 | 260.20 | 83.13 | 23.15 | | 467.53 | | 2.47 |
| LM77 | 63.20 | 148.40 | 190.30 | 272.70 | 100.00 | 107.20 | 191.10 | 247.80 | 75.61 | 38.08 | | 97.43 | | 10.16 |
| LM78 | 40.40 | 69.30 | 136.40 | 196.20 | 73.50 | 93.40 | 207.50 | 265.90 | 77.15 | 35.74 | | 208.57 | | 7.20 |
| LM79 | 42.80 | 136.70 | 156.00 | 296.90 | 68.80 | 106.60 | 114.10 | 202.50 | 73.65 | 48.04 | | 149.87 | | 11.26 |
| LM80 | 54.40 | 205.70 | 179.80 | 402.20 | 112.20 | 139.00 | 111.40 | 258.50 | 50.28 | 43.17 | | 281.41 | | 28.51 |
| LM81 | 51.80 | 107.00 | 140.30 | 308.00 | 69.10 | 205.70 | 184.20 | 212.00 | 63.85 | 44.18 | | 373.91 | | 2.67 |
| LM82 | 61.90 | 134.20 | 143.20 | 270.90 | 44.30 | 162.20 | 185.80 | 162.80 | 109.97 | 53.90 | | 524.83 | | 4.74 |
| LM83 | 43.40 | 128.70 | 99.40 | 271.70 | 38.00 | 287.40 | 165.10 | 178.80 | 92.00 | 27.65 | | 254.76 | | 27.30 |
| LM84 | 69.60 | 176.70 | 141.80 | 268.80 | 51.00 | 207.80 | 100.50 | 170.90 | 87.71 | 53.87 | | 149.29 | | 10.93 |
| LM85 | 89.60 | 167.00 | 196.40 | 362.30 | 59.30 | 155.70 | 160.70 | 217.00 | 29.29 | 57.92 | | 127.82 | | 0.00 |
| LM86 | 55.70 | 69.80 | 132.60 | 208.80 | 60.10 | 111.60 | 138.90 | 191.20 | 111.78 | 16.08 | | 283.56 | | 21.72 |
| LM87 | 34.90 | 86.50 | 97.70 | 211.50 | 63.30 | 47.10 | 145.00 | 186.00 | 37.80 | 17.59 | | 172.64 | | 0.00 |
| LM88 | 45.90 | 63.70 | 69.90 | 156.50 | 30.40 | 17.40 | 164.70 | 212.60 | 90.99 | 29.63 | | 481.61 | | 6.72 |
| LM89 | 42.30 | 148.00 | 99.50 | 349.10 | 55.80 | 84.60 | 118.30 | 174.30 | 109.74 | 40.24 | | 140.76 | | 30.90 |
| LM90 | 65.70 | 96.90 | 186.80 | 271.00 | 70.70 | 145.40 | 159.50 | 242.80 | 56.46 | 47.83 | | 56.45 | | 0.00 |
| LM91 | 58.40 | 92.00 | 116.70 | 214.40 | 64.00 | 112.10 | 155.70 | 247.10 | 87.88 | 42.98 | | 175.13 | | 10.78 |
| LM93 | 56.70 | 96.20 | 130.40 | 201.60 | 44.50 | 204.90 | 144.00 | 177.10 | 118.80 | 24.85 | | 471.08 | | 4.94 |
| LM94 | 31.40 | 53.50 | 118.70 | 130.90 | 91.10 | 101.50 | 136.10 | 214.80 | 82.48 | 25.57 | | 172.51 | | 20.86 |
| LM95 | 31.80 | 43.50 | 79.60 | 94.80 | 50.30 | 62.40 | 95.30 | 118.80 | 43.10 | 32.05 | | 107.78 | | 0.00 |
| LM96 | 77.90 | 129.90 | 206.40 | 244.10 | 58.10 | 98.10 | 162.70 | 244.70 | 134.28 | 46.72 | | 511.55 | | 0.00 |
| LM97 | 79.40 | 105.10 | 147.90 | 185.50 | 61.50 | 134.40 | 106.20 | 227.30 | 50.93 | 37.54 | | 86.28 | | 0.00 |
| LM98 | 38.00 | 126.50 | 144.20 | 294.50 | 39.60 | 112.90 | 150.50 | 200.30 | 89.08 | 24.81 | | 264.91 | | 16.22 |
| LM99 | 51.40 | 127.70 | 162.70 | 312.60 | 37.30 | 124.20 | 140.90 | 155.90 | 30.58 | 42.63 | 159.85 | | 5.66 | |
| LM100 | 71.60 | 123.90 | 171.30 | 291.90 | 96.70 | 217.70 | 169.00 | 159.00 | 165.46 | 44.70 | 243.96 | | 1.46 | |
| Mean | 59.77 | 110.50 | 162.50 | 249.51 | 65.18 | 137.42 | 131.86 | 209.00 | 72.91 | 36.15 | 239.50 | | 12.85 | |
| E1, test environment 1 (greenhouse 2014/15); E2, test environment 2 (field 2014/15); E3, test environment 3 (greenhouse 2015/16); E4, test environment 4 (field 2015/16); WR1, water regime 1 (water stressed); WR2, water regime 2 (control). | | | | | | | | | | | | | | |

| **Table S3 \| Means of agronomic traits, proline content and stress tolerance index (STI) of 96 wheat genotypes when evaluated under stressed and non-stressed conditions across the four testing environments.** | | | | | | | | | | | | | | | | | | | | | |
| --- | --- | --- | --- | --- | --- | --- | --- | --- | --- | --- | --- | --- | --- | --- | --- | --- | --- | --- | --- | --- | --- |
| **Entry** | **DTH** | | **DTM** | | **TN** | | **PH** | | **HL** | | **SPS** | | **KPS** | | **TSW** | | **GY** | |  | **PC1** | |
|  | **WR1** | **WR2** | **WR1** | **WR2** | **WR1** | **WR2** | **WR1** | **WR2** | **WR1** | **WR2** | **WR1** | **WR2** | **WR1** | **WR2** | **WR1** | **WR2** | **WR1** | **WR2** | **STI** | **WR1** | **WR2** |
| LM01 | 44.75 | 44.25 | 96.25 | 97.37 | 3.34 | 4.20 | 68.76 | 73.54 | 8.42 | 8.47 | 14.10 | 14.03 | 34.00 | 37.87 | 30.62 | 32.94 | 112.30 | 170.10 | 0.61 | 153.69 | 18.07 |
| **LM02** | **50.38** | **49.00** | **92.75** | **100.62** | **3.51** | **3.89** | **79.61** | **81.98** | **9.28** | **9.29** | **14.73** | **14.88** | **37.92** | **43.30** | **28.23** | **36.88** | **122.00** | **195.00** | **0.76** | **350.65** | **36.51** |
| **LM03** | **51.50** | **51.88** | **91.75** | **100.00** | **3.79** | **4.53** | **83.16** | **86.58** | **9.50** | **9.54** | **15.88** | **15.88** | **36.07** | **38.15** | **28.07** | **39.98** | **126.00** | **210.30** | **0.85** | **159.30** | **20.22** |
| **LM04** | **56.25** | **56.88** | **101.87** | **105.00** | **3.48** | **4.19** | **76.93** | **77.68** | **10.41** | **10.04** | **16.85** | **16.33** | **39.40** | **41.05** | **33.43** | **36.51** | **138.90** | **192.20** | **0.86** | **93.77** | **13.08** |
| **LM05** | **58.88** | **60.00** | **106.62** | **108.87** | **3.42** | **5.06** | **71.78** | **80.88** | **8.91** | **8.95** | **15.43** | **16.40** | **35.32** | **46.32** | **32.50** | **34.34** | **115.30** | **239.80** | **0.89** | **173.52** | **20.82** |
| LM06 | 52.63 | 53.75 | 98.75 | 104.75 | 3.35 | 4.47 | 77.66 | 82.54 | 9.85 | 9.90 | 15.15 | 15.50 | 36.92 | 39.80 | 32.29 | 37.98 | 121.50 | 201.30 | 0.78 | 309.59 | 25.75 |
| LM07 | 56.88 | 58.13 | 103.25 | 106.00 | 3.69 | 4.85 | 79.38 | 82.53 | 7.97 | 8.07 | 15.30 | 16.14 | 33.60 | 40.69 | 29.84 | 32.06 | 113.30 | 196.60 | 0.71 | 184.32 | 27.05 |
| LM08 | 50.25 | 49.63 | 93.62 | 94.37 | 3.71 | 4.34 | 68.47 | 74.13 | 8.02 | 8.13 | 13.99 | 14.35 | 33.15 | 35.80 | 27.69 | 33.87 | 109.60 | 165.00 | 0.58 | 201.19 | 29.42 |
| **LM09** | **59.38** | **56.88** | **101.62** | **106.87** | **3.84** | **5.83** | **69.35** | **83.55** | **8.86** | **8.73** | **14.63** | **14.40** | **29.27** | **33.82** | **32.23** | **37.69** | **111.30** | **234.80** | **0.84** | **114.76** | **9.82** |
| LM10 | 57.50 | 58.75 | 102.50 | 106.62 | 3.09 | 4.41 | 72.59 | 81.22 | 10.26 | 10.42 | 15.88 | 16.13 | 36.37 | 40.37 | 35.24 | 35.57 | 114.80 | 198.00 | 0.73 | 180.66 | 19.78 |
| LM11 | 52.50 | 51.38 | 97.75 | 97.87 | 3.21 | 4.25 | 73.84 | 79.93 | 9.08 | 9.50 | 14.83 | 16.50 | 31.37 | 42.42 | 28.91 | 31.03 | 95.40 | 171.10 | 0.52 | 196.93 | 24.05 |
| **LM12** | **52.00** | **51.50** | **94.87** | **96.37** | **3.80** | **4.67** | **75.18** | **79.93** | **8.25** | **8.52** | **14.35** | **14.70** | **27.47** | **31.45** | **28.62** | **39.82** | **95.20** | **179.20** | **0.55** | **122.78** | **31.27** |
| LM13 | 55.63 | 53.13 | 98.87 | 99.87 | 3.51 | 5.05 | 80.07 | 83.98 | 8.53 | 8.94 | 15.38 | 15.93 | 35.20 | 41.07 | 29.36 | 32.69 | 108.10 | 212.70 | 0.74 | 48.62 | 21.77 |
| LM14 | 44.25 | 44.25 | 98.75 | 98.87 | 3.84 | 3.83 | 68.22 | 72.00 | 7.74 | 7.92 | 12.80 | 13.29 | 29.17 | 33.81 | 32.07 | 33.84 | 117.20 | 138.50 | 0.52 | 145.79 | 28.61 |
| LM15 | 57.50 | 56.88 | 103.12 | 107.75 | 3.59 | 4.61 | 75.95 | 85.81 | 9.61 | 9.92 | 15.58 | 16.98 | 37.62 | 45.90 | 30.33 | 36.47 | 135.00 | 231.70 | 1.00 | 307.70 | 24.92 |
| LM16 | 53.25 | 53.75 | 96.62 | 98.37 | 2.93 | 4.44 | 77.07 | 82.72 | 9.17 | 9.81 | 13.95 | 15.35 | 33.30 | 37.37 | 33.28 | 34.06 | 96.40 | 174.50 | 0.54 | 127.82 | 21.97 |
| **LM17** | **50.25** | **50.25** | **95.12** | **100.25** | **3.29** | **5.32** | **71.87** | **78.11** | **8.78** | **9.20** | **14.00** | **14.68** | **35.32** | **38.52** | **27.92** | **35.28** | **103.60** | **226.90** | **0.75** | **222.73** | **38.11** |
| LM18 | 47.88 | 47.38 | 97.25 | 100.37 | 3.80 | 3.70 | 69.90 | 68.37 | 8.12 | 8.04 | 14.10 | 13.59 | 33.50 | 29.27 | 30.07 | 33.48 | 120.80 | 116.00 | 0.45 | 135.57 | 32.72 |
| LM19 | 55.63 | 56.25 | 104.25 | 108.87 | 4.10 | 4.52 | 76.88 | 81.59 | 8.37 | 8.30 | 15.08 | 14.78 | 24.90 | 28.75 | 38.25 | 40.03 | 116.50 | 164.00 | 0.61 | 149.11 | 30.41 |
| LM20 | 61.25 | 60.00 | 103.72 | 106.00 | 2.86 | 4.32 | 69.93 | 72.73 | 9.14 | 8.95 | 15.75 | 15.68 | 22.47 | 29.82 | 37.10 | 38.34 | 69.90 | 151.30 | 0.34 | 190.68 | 25.39 |
| **LM21** | **56.25** | **56.88** | **102.00** | **107.75** | **3.15** | **4.73** | **71.33** | **76.85** | **8.34** | **8.62** | **15.18** | **15.73** | **35.25** | **39.77** | **32.56** | **39.95** | **109.50** | **233.00** | **0.82** | **90.11** | **38.92** |
| **LM22** | **56.24** | **57.50** | **101.12** | **104.12** | **4.09** | **4.73** | **74.61** | **79.06** | **8.59** | **8.72** | **14.68** | **15.35** | **32.20** | **38.72** | **32.49** | **36.37** | **143.30** | **201.10** | **0.92** | **214.89** | **26.00** |
| **LM23** | **56.25** | **57.50** | **98.87** | **103.50** | **3.95** | **6.08** | **80.68** | **88.64** | **9.54** | **10.15** | **14.78** | **16.23** | **34.52** | **36.12** | **32.16** | **38.50** | **128.80** | **258.40** | **1.07** | **217.09** | **19.58** |
| LM24 | 58.75 | 58.13 | 101.00 | 104.87 | 3.30 | 3.68 | 76.97 | 80.64 | 10.56 | 10.38 | 17.48 | 17.65 | 36.85 | 42.02 | 32.35 | 34.61 | 118.60 | 159.60 | 0.61 | 101.16 | 25.75 |
| LM25 | 56.25 | 56.88 | 100.12 | 105.37 | 2.95 | 4.69 | 69.96 | 76.22 | 8.49 | 8.50 | 15.45 | 16.10 | 31.80 | 44.10 | 29.59 | 34.60 | 79.00 | 213.40 | 0.54 | 36.37 | 27.16 |
| LM26 | 45.50 | 43.63 | 92.87 | 98.25 | 3.43 | 4.87 | 68.74 | 68.92 | 8.36 | 8.44 | 14.43 | 14.58 | 34.65 | 36.02 | 25.78 | 32.81 | 99.60 | 185.60 | 0.59 | 120.63 | 35.90 |
| LM27 | 54.38 | 56.25 | 95.50 | 98.25 | 3.41 | 3.97 | 76.60 | 75.03 | 8.37 | 8.20 | 15.78 | 15.60 | 37.75 | 39.27 | 31.96 | 33.56 | 126.90 | 162.30 | 0.66 | 82.92 | 25.84 |
| LM28 | 56.88 | 55.63 | 100.37 | 105.12 | 3.01 | 4.20 | 73.01 | 78.82 | 9.25 | 9.25 | 14.70 | 16.03 | 30.20 | 38.27 | 37.11 | 38.13 | 93.50 | 188.80 | 0.57 | 103.11 | 19.16 |
| **LM29** | **55.63** | **58.13** | **103.12** | **107.25** | **3.80** | **4.66** | **76.28** | **79.20** | **8.39** | **8.77** | **15.03** | **15.53** | **37.37** | **38.85** | **34.70** | **37.20** | **149.70** | **205.00** | **0.98** | **67.89** | **46.72** |
| LM30 | 55.63 | 56.25 | 98.87 | 106.12 | 2.91 | 3.90 | 73.87 | 82.06 | 9.83 | 9.71 | 16.78 | 17.60 | 40.50 | 46.52 | 29.68 | 37.85 | 112.70 | 211.70 | 0.77 | 239.78 | 17.56 |
| LM31 | 52.00 | 54.38 | 96.50 | 101.87 | 3.52 | 4.24 | 78.32 | 78.34 | 9.67 | 9.24 | 14.13 | 13.30 | 32.72 | 30.79 | 33.11 | 42.82 | 125.50 | 175.00 | 0.70 | 192.59 | 28.92 |
| LM32 | 54.50 | 54.38 | 99.12 | 106.50 | 2.71 | 4.62 | 80.02 | 87.17 | 8.74 | 9.06 | 15.25 | 15.68 | 37.32 | 42.37 | 31.40 | 34.64 | 98.50 | 200.90 | 0.63 | 87.17 | 22.02 |
| LM33 | 53.75 | 55.63 | 102.37 | 108.25 | 3.43 | 3.40 | 73.97 | 76.86 | 8.40 | 8.39 | 16.80 | 16.10 | 34.15 | 37.00 | 29.21 | 31.47 | 119.50 | 126.00 | 0.48 | 167.88 | 26.24 |
| LM34 | 48.25 | 49.50 | 94.75 | 98.50 | 3.17 | 3.98 | 73.15 | 77.58 | 8.49 | 8.32 | 14.65 | 15.15 | 32.20 | 30.79 | 28.42 | 34.82 | 89.00 | 142.00 | 0.41 | 142.14 | 26.76 |
| LM35 | 52.00 | 50.38 | 97.87 | 101.62 | 3.52 | 5.30 | 76.85 | 82.63 | 8.35 | 8.71 | 15.30 | 15.85 | 37.45 | 39.67 | 30.96 | 34.31 | 125.30 | 226.20 | 0.91 | 176.90 | 20.44 |
| LM36 | 55.63 | 57.50 | 101.37 | 106.12 | 3.02 | 4.80 | 76.56 | 82.93 | 9.05 | 9.42 | 14.48 | 15.50 | 34.75 | 38.62 | 33.37 | 36.85 | 106.70 | 207.70 | 0.71 | 85.64 | 22.83 |
| LM37 | 53.25 | 52.50 | 99.50 | 105.25 | 2.44 | 3.42 | 69.98 | 72.72 | 8.79 | 8.80 | 15.30 | 15.98 | 33.97 | 36.29 | 34.45 | 37.89 | 89.20 | 144.70 | 0.41 | 191.28 | 12.18 |
| LM38 | 55.00 | 53.13 | 100.00 | 107.75 | 2.78 | 3.08 | 76.32 | 78.90 | 8.94 | 9.57 | 17.28 | 17.90 | 44.25 | 43.62 | 31.69 | 36.56 | 120.60 | 158.60 | 0.61 | 129.15 | 23.28 |
| LM39 | 50.75 | 50.88 | 98.00 | 103.00 | 3.11 | 4.58 | 74.65 | 81.41 | 8.32 | 9.08 | 14.68 | 16.13 | 35.85 | 39.30 | 29.62 | 33.21 | 101.70 | 180.00 | 0.59 | 83.67 | 25.81 |
| LM40 | 50.75 | 51.38 | 93.12 | 95.37 | 3.30 | 4.68 | 75.32 | 78.58 | 9.41 | 9.51 | 15.95 | 16.80 | 36.25 | 40.02 | 27.13 | 35.66 | 101.90 | 218.30 | 0.71 | 166.19 | 20.01 |
| LM41 | 54.38 | 55.75 | 100.12 | 107.25 | 2.67 | 4.45 | 75.09 | 80.53 | 9.89 | 10.26 | 16.18 | 17.23 | 38.42 | 43.05 | 27.57 | 35.12 | 94.50 | 209.30 | 0.63 | 381.18 | 32.27 |
| LM42 | 57.50 | 59.39 | 102.00 | 107.65 | 3.02 | 4.24 | 77.63 | 79.09 | 8.87 | 9.32 | 16.88 | 17.98 | 31.90 | 41.15 | 25.86 | 30.41 | 77.70 | 168.40 | 0.42 | 105.16 | 25.56 |
| LM43 | 60.63 | 59.38 | 103.62 | 108.87 | 2.99 | 5.00 | 77.02 | 87.65 | 10.15 | 10.47 | 15.20 | 16.23 | 26.77 | 35.65 | 35.47 | 40.27 | 86.80 | 216.30 | 0.60 | 127.35 | 15.38 |
| LM44 | 52.63 | 54.38 | 94.75 | 98.12 | 3.43 | 3.95 | 83.02 | 85.77 | 9.82 | 10.00 | 15.73 | 16.00 | 42.02 | 45.47 | 26.87 | 28.93 | 123.80 | 162.50 | 0.65 | 97.96 | 19.56 |
| **LM45** | **59.38** | **56.25** | **103.62** | **107.75** | **3.31** | **4.56** | **73.86** | **81.48** | **10.14** | **10.24** | **15.45** | **16.23** | **33.72** | **38.00** | **35.00** | **41.77** | **115.50** | **218.60** | **0.81** | **129.15** | **18.27** |
| LM46 | 53.25 | 55.63 | 100.00 | 105.12 | 3.21 | 4.47 | 72.02 | 79.04 | 9.63 | 9.71 | 15.70 | 16.35 | 31.42 | 38.00 | 35.98 | 36.88 | 113.30 | 192.00 | 0.70 | 155.05 | 14.94 |
| LM47 | 55.00 | 56.25 | 100.12 | 105.00 | 3.18 | 4.44 | 75.97 | 78.06 | 9.74 | 9.42 | 15.00 | 14.90 | 32.05 | 31.50 | 34.23 | 38.26 | 109.00 | 165.10 | 0.58 | 180.78 | 39.72 |
| LM48 | 55.63 | 58.13 | 97.87 | 103.62 | 2.59 | 4.20 | 79.30 | 84.30 | 10.42 | 10.24 | 15.60 | 15.40 | 33.62 | 35.07 | 34.01 | 36.42 | 91.80 | 167.30 | 0.49 | 75.89 | 17.76 |
| LM49 | 57.50 | 60.63 | 103.25 | 109.87 | 2.76 | 4.19 | 77.72 | 86.30 | 9.28 | 9.78 | 15.15 | 16.23 | 30.65 | 33.77 | 33.94 | 36.71 | 89.80 | 158.20 | 0.46 | 61.73 | 32.18 |
| LM50 | 45.50 | 44.88 | 94.25 | 98.12 | 3.30 | 4.54 | 63.59 | 65.10 | 5.88 | 6.16 | 11.65 | 12.50 | 27.15 | 32.72 | 30.28 | 32.43 | 85.60 | 153.60 | 0.42 | 79.57 | 29.84 |
| LM51 | 44.75 | 45.25 | 93.25 | 97.25 | 3.86 | 4.52 | 60.62 | 65.37 | 6.61 | 6.83 | 11.95 | 12.45 | 27.80 | 29.32 | 30.02 | 35.87 | 105.60 | 148.50 | 0.50 | 127.93 | 28.76 |

| **Table S3 \| (continued)** | | | | | | | | | | | | | | | | | | | | | |
| --- | --- | --- | --- | --- | --- | --- | --- | --- | --- | --- | --- | --- | --- | --- | --- | --- | --- | --- | --- | --- | --- |
| Entry | **DTH** | | **DTM** | | **TN** | | **PH** | | **HL** | | **SPS** | | **KPS** | | **TSW** | | **GY** | |  | **PC1** | |
|  | **WR1** | **WR2** | **WR1** | **WR2** | **WR1** | **WR2** | **WR1** | **WR2** | **WR1** | **WR2** | **WR1** | **WR2** | **WR1** | **WR2** | **WR1** | **WR2** | **WR1** | **WR2** | **STI** | **WR1** | **WR2** |
| LM52 | 50.75 | 50.63 | 97.00 | 99.12 | 3.73 | 5.37 | 65.30 | 69.57 | 6.42 | 6.99 | 12.33 | 13.44 | 25.75 | 31.02 | 29.97 | 38.14 | 94.10 | 198.10 | 0.60 | 143.08 | 27.75 |
| LM53 | 44.88 | 44.25 | 96.25 | 96.25 | 3.68 | 5.86 | 60.42 | 61.18 | 7.10 | 7.24 | 12.68 | 12.73 | 27.14 | 29.57 | 30.90 | 32.61 | 100.00 | 180.50 | 0.58 | 95.53 | 18.07 |
| LM54 | 58.75 | 58.75 | 102.25 | 105.62 | 2.72 | 4.40 | 73.52 | 76.40 | 7.02 | 7.44 | 15.88 | 18.10 | 32.30 | 37.45 | 35.71 | 38.26 | 95.20 | 188.80 | 0.58 | 133.63 | 26.93 |
| LM55 | 52.13 | 50.75 | 97.00 | 101.37 | 3.27 | 4.64 | 71.65 | 73.55 | 7.61 | 7.90 | 14.65 | 15.70 | 35.45 | 40.70 | 32.21 | 31.00 | 116.80 | 184.60 | 0.69 | 92.53 | 24.22 |
| LM56 | 53.75 | 53.75 | 99.00 | 102.25 | 3.52 | 3.72 | 72.46 | 77.41 | 7.92 | 7.89 | 15.85 | 16.35 | 36.37 | 37.15 | 29.37 | 31.57 | 116.50 | 139.60 | 0.52 | 106.63 | 23.12 |
| LM57 | 52.50 | 55.63 | 96.87 | 100.87 | 3.16 | 4.13 | 73.20 | 75.58 | 8.84 | 8.80 | 16.05 | 16.40 | 37.67 | 43.35 | 30.32 | 32.39 | 109.30 | 177.60 | 0.62 | 114.89 | 13.45 |
| LM58 | 55.00 | 57.50 | 101.87 | 106.62 | 3.04 | 4.03 | 76.19 | 82.82 | 8.86 | 8.63 | 15.48 | 15.25 | 36.55 | 38.00 | 27.75 | 32.14 | 97.30 | 155.40 | 0.48 | 186.52 | 24.54 |
| LM59 | 60.00 | 61.25 | 104.25 | 107.62 | 3.49 | 4.84 | 74.60 | 80.34 | 11.28 | 11.33 | 16.15 | 16.90 | 31.97 | 34.37 | 32.33 | 34.18 | 108.20 | 172.40 | 0.60 | 219.63 | 34.25 |
| LM60 | 54.38 | 56.25 | 101.12 | 104.12 | 3.16 | 3.93 | 78.03 | 80.11 | 8.67 | 8.61 | 13.65 | 14.40 | 33.37 | 35.60 | 31.50 | 36.29 | 105.70 | 156.30 | 0.53 | 130.30 | 26.81 |
| LM61 | 45.38 | 44.75 | 93.12 | 96.62 | 3.32 | 3.52 | 59.92 | 62.26 | 5.97 | 5.96 | 10.98 | 10.63 | 19.72 | 20.80 | 29.09 | 30.18 | 50.30 | 75.00 | 0.12 | 85.99 | 16.53 |
| LM62 | 60.63 | 58.13 | 104.87 | 106.87 | 1.99 | 3.33 | 64.06 | 65.64 | 9.78 | 10.21 | 15.78 | 17.14 | 32.20 | 39.75 | 27.15 | 30.71 | 58.70 | 124.40 | 0.23 | 165.07 | 26.65 |
| LM64 | 44.63 | 43.63 | 99.37 | 98.75 | 4.74 | 4.00 | 60.20 | 62.80 | 6.33 | 6.46 | 10.21 | 10.50 | 23.42 | 21.57 | 30.65 | 31.75 | 110.90 | 97.00 | 0.34 | 222.79 | 18.56 |
| LM65 | 55.00 | 53.13 | 105.12 | 105.25 | 3.54 | 4.67 | 62.27 | 63.80 | 7.73 | 7.80 | 12.25 | 13.10 | 29.25 | 32.42 | 26.17 | 28.19 | 88.10 | 135.10 | 0.38 | 96.12 | 23.57 |
| LM66 | 42.38 | 43.63 | 97.62 | 100.62 | 4.73 | 4.58 | 62.36 | 63.23 | 6.15 | 6.29 | 11.78 | 12.08 | 24.75 | 28.17 | 31.20 | 31.66 | 118.80 | 134.30 | 0.51 | 211.07 | 16.00 |
| LM67 | 43.63 | 43.63 | 96.62 | 100.12 | 3.28 | 3.64 | 58.51 | 62.89 | 6.69 | 7.01 | 12.58 | 12.05 | 27.75 | 30.70 | 31.33 | 32.69 | 95.60 | 123.10 | 0.38 | 107.60 | 14.44 |
| LM68 | 48.13 | 50.63 | 96.25 | 101.75 | 3.39 | 4.28 | 59.05 | 64.64 | 6.67 | 7.06 | 13.07 | 14.68 | 30.23 | 36.42 | 21.05 | 24.89 | 60.90 | 130.80 | 0.26 | 234.88 | 15.06 |
| LM70 | 51.25 | 49.38 | 94.60 | 99.62 | 3.29 | 3.69 | 65.27 | 71.03 | 8.39 | 8.51 | 13.38 | 13.83 | 32.47 | 37.72 | 24.64 | 28.06 | 82.20 | 128.10 | 0.34 | 234.36 | 20.41 |
| LM71 | 53.88 | 53.88 | 94.87 | 101.87 | 4.05 | 4.86 | 75.31 | 80.01 | 9.29 | 9.46 | 14.43 | 15.05 | 34.10 | 36.87 | 29.44 | 32.86 | 131.50 | 181.90 | 0.77 | 97.37 | 42.91 |
| LM72 | 49.25 | 49.13 | 94.00 | 102.75 | 3.10 | 3.43 | 77.74 | 80.85 | 8.90 | 8.62 | 14.60 | 13.80 | 31.95 | 32.95 | 27.28 | 33.22 | 82.30 | 119.90 | 0.32 | 216.15 | 28.49 |
| LM73 | 52.13 | 55.75 | 98.87 | 103.87 | 3.18 | 5.59 | 80.18 | 83.00 | 8.49 | 8.68 | 14.50 | 14.88 | 37.40 | 36.88 | 28.94 | 35.68 | 111.80 | 220.70 | 0.79 | 137.39 | 33.95 |
| LM75 | 55.25 | 56.38 | 97.25 | 103.62 | 3.83 | 4.14 | 77.65 | 80.25 | 8.95 | 8.95 | 15.23 | 15.20 | 34.55 | 35.85 | 29.49 | 36.57 | 119.30 | 165.80 | 0.63 | 218.87 | 29.74 |
| LM76 | 48.63 | 49.25 | 98.00 | 101.50 | 3.29 | 4.49 | 70.78 | 74.16 | 8.67 | 9.00 | 13.42 | 14.45 | 29.44 | 37.57 | 34.32 | 38.05 | 95.60 | 197.00 | 0.60 | 275.33 | 12.81 |
| LM77 | 55.00 | 55.00 | 99.75 | 103.00 | 3.70 | 4.14 | 87.90 | 89.71 | 9.10 | 9.10 | 14.45 | 14.43 | 31.72 | 33.50 | 36.79 | 45.43 | 136.20 | 194.00 | 0.85 | 86.52 | 24.12 |
| LM78 | 53.88 | 55.13 | 100.25 | 102.62 | 3.98 | 5.07 | 72.46 | 80.24 | 6.77 | 7.17 | 14.73 | 14.65 | 26.17 | 25.62 | 34.47 | 38.15 | 114.40 | 156.20 | 0.57 | 142.86 | 21.47 |
| LM79 | 54.50 | 53.25 | 99.37 | 100.87 | 3.13 | 4.73 | 85.91 | 86.33 | 9.03 | 9.07 | 14.63 | 14.48 | 28.87 | 30.50 | 35.77 | 42.24 | 95.40 | 185.70 | 0.57 | 111.76 | 29.65 |
| LM80 | 59.38 | 63.99 | 100.78 | 106.75 | 3.34 | 4.57 | 85.98 | 87.19 | 8.33 | 8.83 | 15.28 | 16.80 | 37.02 | 46.85 | 29.54 | 37.31 | 114.40 | 251.30 | 0.92 | 165.84 | 35.84 |
| LM81 | 58.75 | 58.75 | 102.25 | 106.50 | 3.02 | 4.73 | 75.30 | 89.07 | 9.79 | 9.87 | 16.08 | 16.38 | 32.87 | 37.77 | 36.24 | 38.59 | 111.40 | 208.20 | 0.74 | 218.88 | 23.42 |
| LM82 | 56.38 | 56.88 | 97.25 | 106.37 | 3.77 | 4.74 | 71.16 | 75.72 | 8.85 | 8.71 | 15.63 | 16.05 | 38.30 | 43.40 | 23.37 | 29.16 | 108.80 | 182.50 | 0.64 | 317.40 | 29.32 |
| LM83 | 60.00 | 58.75 | 102.12 | 107.62 | 3.03 | 4.76 | 74.15 | 80.15 | 9.06 | 9.29 | 16.05 | 16.40 | 32.77 | 42.35 | 29.59 | 34.99 | 86.50 | 216.60 | 0.60 | 173.38 | 27.48 |
| LM84 | 60.00 | 62.50 | 105.00 | 109.62 | 3.22 | 6.33 | 79.10 | 90.55 | 9.17 | 9.44 | 15.40 | 16.70 | 33.70 | 38.35 | 27.46 | 28.81 | 90.70 | 206.10 | 0.60 | 118.50 | 32.40 |
| **LM85** | **56.88** | **56.25** | **100.25** | **104.00** | **3.74** | **4.86** | **72.83** | **79.82** | **8.60** | **8.92** | **15.03** | **15.67** | **35.47** | **35.59** | **30.75** | **35.09** | **126.50** | **225.50** | **0.91** | **78.56** | **28.96** |
| LM86 | 50.25 | 50.63 | 99.12 | 101.62 | 3.77 | 4.82 | 66.61 | 64.33 | 8.55 | 8.59 | 14.13 | 14.60 | 33.35 | 34.32 | 24.66 | 28.46 | 96.80 | 145.40 | 0.45 | 197.67 | 18.90 |
| LM87 | 48.00 | 47.25 | 94.62 | 102.62 | 2.93 | 4.03 | 71.85 | 69.59 | 8.39 | 7.98 | 14.53 | 12.92 | 32.42 | 30.95 | 29.40 | 32.55 | 85.20 | 132.80 | 0.36 | 105.22 | 8.79 |
| LM88 | 49.25 | 49.75 | 93.75 | 100.87 | 3.08 | 3.68 | 71.44 | 70.85 | 8.30 | 7.92 | 14.15 | 13.28 | 33.05 | 30.40 | 24.06 | 29.90 | 77.70 | 112.50 | 0.28 | 286.30 | 18.17 |
| LM89 | 57.50 | 55.63 | 106.25 | 105.37 | 3.16 | 5.72 | 76.84 | 81.84 | 8.46 | 8.69 | 14.60 | 15.40 | 31.25 | 39.60 | 26.70 | 26.12 | 79.00 | 189.00 | 0.48 | 125.25 | 35.57 |
| LM90 | 53.38 | 55.00 | 96.75 | 101.75 | 3.48 | 4.11 | 85.21 | 90.68 | 9.33 | 9.34 | 14.30 | 14.68 | 30.42 | 32.17 | 36.61 | 45.00 | 120.70 | 189.00 | 0.73 | 56.46 | 23.92 |
| LM91 | 46.13 | 46.75 | 98.12 | 100.50 | 3.85 | 5.05 | 61.81 | 69.15 | 7.07 | 7.41 | 12.55 | 13.48 | 27.97 | 29.75 | 30.95 | 35.11 | 98.70 | 166.40 | 0.53 | 131.50 | 26.88 |
| LM93 | 55.13 | 55.00 | 95.50 | 102.87 | 2.93 | 4.34 | 79.39 | 84.15 | 9.76 | 9.85 | 14.48 | 14.95 | 30.20 | 35.30 | 33.46 | 37.70 | 93.90 | 170.00 | 0.51 | 294.94 | 14.90 |
| LM94 | 55.00 | 53.75 | 98.25 | 103.87 | 3.60 | 4.44 | 72.07 | 79.27 | 6.83 | 7.14 | 14.23 | 14.02 | 26.40 | 21.80 | 32.75 | 40.42 | 94.30 | 125.10 | 0.38 | 127.50 | 23.21 |
| LM95 | 45.50 | 48.00 | 94.50 | 97.37 | 2.57 | 2.81 | 60.90 | 62.54 | 7.08 | 6.97 | 12.33 | 12.60 | 29.30 | 30.35 | 28.68 | 31.45 | 64.30 | 79.90 | 0.16 | 75.44 | 16.02 |
| LM96 | 55.63 | 55.63 | 102.75 | 106.25 | 4.08 | 4.65 | 83.28 | 88.27 | 7.89 | 7.99 | 15.23 | 15.13 | 35.47 | 36.05 | 27.99 | 35.69 | 126.30 | 179.20 | 0.73 | 322.91 | 23.36 |
| LM97 | 56.25 | 59.38 | 103.87 | 105.87 | 2.94 | 3.80 | 74.95 | 82.92 | 9.83 | 10.26 | 16.03 | 16.98 | 35.72 | 42.77 | 31.40 | 33.55 | 98.80 | 163.10 | 0.52 | 68.60 | 18.77 |
| LM98 | 55.63 | 56.25 | 96.00 | 101.25 | 3.25 | 4.43 | 76.50 | 84.93 | 9.92 | 9.95 | 15.13 | 15.73 | 35.00 | 42.00 | 24.33 | 31.59 | 93.10 | 183.50 | 0.55 | 176.99 | 20.51 |
| LM99 | 52.13 | 53.25 | 93.75 | 99.37 | 3.75 | 5.63 | 74.89 | 76.85 | 8.34 | 8.15 | 13.88 | 13.95 | 32.85 | 33.62 | 24.16 | 28.69 | 98.10 | 180.10 | 0.57 | 95.21 | 24.15 |
| LM100 | 61.88 | 61.88 | 106.00 | 108.00 | 3.48 | 4.03 | 79.49 | 90.06 | 9.77 | 10.04 | 14.73 | 15.80 | 33.57 | 41.07 | 36.50 | 38.77 | 127.20 | 198.10 | 0.81 | 204.71 | 23.08 |
| **mean** | **53.45** | **53.80** | **98.97** | **103.13** | **3.36** | **4.45** | **73.52** | **78.03** | **8.65** | **8.79** | **14.71** | **15.18** | **32.87** | **36.52** | **30.72** | **34.93** | **104.83** | **176.60** | **0.60** | **156.20** | **24.50** |
| DTH, days to 50% heading; DTM, days to maturity; GY, grain yield per plot; SL, spike length; KPS, number of kennels per plant; PC, Proline content; PH, plant height; TN, number of productive tillers; SPS, number of spikelets per spike; TSW, thousand kernel weight; WR1, water regime 1 (water stress); WR2, water regime 2 (control); STI, stress tolerance index, genotypes highlighted in bold were selected for breeding. | | | | | | | | | | | | | | | | | | | | | |
